# An Activity‐Based Probe Targeting Non‐Catalytic, Highly Conserved Amino Acid Residues within Bromodomains

**DOI:** 10.1002/anie.201807825

**Published:** 2018-12-27

**Authors:** Melissa D'Ascenzio, Kathryn M. Pugh, Rebecca Konietzny, Georgina Berridge, Cynthia Tallant, Shaima Hashem, Octovia Monteiro, Jason R. Thomas, Markus Schirle, Stefan Knapp, Brian Marsden, Oleg Fedorov, Chas Bountra, Benedikt M. Kessler, Paul E. Brennan

**Affiliations:** ^1^ Structural Genomic Consortium (SGC) University of Oxford Oxford OX3 7DQ UK; ^2^ Target Discovery Institute (TDI) University of Oxford Oxford OX3 7FZ UK; ^3^ Novartis Institute for BioMedical Research (NIBR) 180 Massachusetts Ave Cambridge MA 02139 USA; ^4^ Institute for Pharmaceutical Chemistry and Buchmann Institute for Life Sciences Johann Wolfgang Goethe-University 60438 Frankfurt am Main Germany

**Keywords:** activity-based protein profiling, bromodomain, chemical proteomics, covalent probes, epigenetics

## Abstract

Bromodomain‐containing proteins are epigenetic modulators involved in a wide range of cellular processes, from recruitment of transcription factors to pathological disruption of gene regulation and cancer development. Since the druggability of these acetyl‐lysine reader domains was established, efforts were made to develop potent and selective inhibitors across the entire family. Here we report the development of a small molecule‐based approach to covalently modify recombinant and endogenous bromodomain‐containing proteins by targeting a conserved lysine and a tyrosine residue in the variable ZA or BC loops. Moreover, the addition of a reporter tag allowed in‐gel visualization and pull‐down of the desired bromodomains.

Chemical proteomics methodologies such as activity‐based protein profiling (ABPP) have been developed to complement genetic manipulation and interrogate the proteome to capture the functional state of proteins in cells and tissues.^1^ Through the use of covalent inhibitors, ABPP has provided quantitative readouts of the functional state of individual or multiple enzymes, taking into account allosteric, intrasteric, and post‐translational control.^2^ Furthermore, thanks to their broad spectrum of selectivity, ABPP probes have been used to screen prospective inhibitors against a wide panel of proteins and show target engagement in the cellular environment.^3^, ^4^ Despite its widespread application to several different enzymatic families including kinases,^5^ serine hydrolases,^6^ and cysteine/threonine proteases,^7^ only minor progress has been made with reference to epigenetic proteins. The value of developing activity‐based probes directed against epigenetic modulators finds its rationale in the emerging potential of epigenetic proteins as therapeutic targets,^8^ and it has been emphasized by recently reported successful attempts to use similar strategies in order to profile lysine methyl transferases,^9^, ^10^, ^11^ or histone deacetylases.^12^, ^13^

However, a significant amount of the afore mentioned studies rely on photo‐affinity labelling techniques for the development of efficient probes, mostly because of the lack of activated or easily targetable nucleophilic amino acid residues in the active sites of these proteins. While there are no reports of effective ABPP probes against native bromodomains (BRDs), Daguer et al. recently reported on the discovery of small molecules that are able to engage with a conserved cysteine residue in the binding pocket when incubated with truncated bromodomains.^14^ These small reader domains that specifically recognize the *ϵ*‐*N*‐acetylated (KAc) lysine mark on histone tails, have been identified as functional modules within at least 46 human proteins that are known to be involved in the regulation of a wide range of cellular events, from transcription factors recruitment to chromatin remodeling, and in the occurrence of several types of cancer.^15^, ^16^ Bromodomain‐containing proteins constitute a class of epigenetic modulators that are particularly challenging to profile by ABPP, since they lack enzymatic activity and are deficient of activated amino acid residues in their ligand binding sites. For this challenging task to be achieved, new activity‐based probe should be endowed with widespread activity across the BRD family, in order to target most of the 61 bromodomains encoded by the human proteome. Bromosporine (BSP) is currently the most broad spectrum bromodomain inhibitor; it is characterized by in vitro K_D_ values in the nanomolar/low micromolar range, especially against the BET family, and sub‐micromoal cellular activity against the first bromodomain of BRD4, BRD7, BRD9, CREBBP, and CECR2.^17^ Therefore, its scaffold was considered as a perfect starting point for the rational design of a novel broad spectrum activity‐based BRD probe. Through a series of preliminary sequence alignment and docking studies, we observed that the methyl sulfonamide group of BSP pointed towards one of three lysine residues that are fairly conserved in the BC loop of BRDs belonging to different sub‐families, and corresponded to residue 85, 90 or 91 in the first BRD of BRD4 (BRD4(1)) (Figure 1).

**Figure 1:**
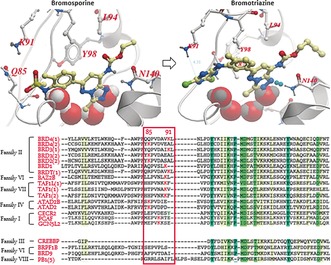
Crystal structure of bromosporine and docking of bromotriazine in BRD4(1), and sequence alignment of BRDs belonging to different families. The methylsulfonamide moiety of BSP was replaced by a dichlorotriazine warhead in order to selectively target one of three lysines (red K in red box) present in the variable BC loop of bromodomains belonging to different families. The amino acid sequences were aligned using highly conserved residues (columns highlighted in green).

As lysines are potentially nucleophilic amino acids that have been reported to be targetable by covalent labelling,^18^ we believed it would be possible to develop a novel BRD covalent probe by replacing the sulfonamide scaffold of BSP with a reactive warhead, and subsequently introduce a clickable alkyne handle on the solvent exposed ethyl carbamate moiety to allow tagged protein pull‐down and in‐gel visualization (Figure 2).

**Figure 2:**
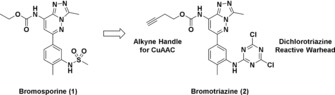
Design of bromosporine derived covalent probe bromotriazine (BTZ). A clickable alkyne handle was added to the ethyl carbamate moiety of bromosporine, while a dichlorotriazine electrophile was introduced to replace the methylsulfonamide scaffold and generate bromotriazine **2**.

In the search for the most appropriate electrophile to use as a warhead for our probe, we found that Shannon et al. had reported the discovery of a dichlorotriazine reactive moiety that was capable of selectively labelling a range of lysine residues within the proteome of HeLa cells.^19^ A series of docking experiments suggested that the dichlorotriazine moiety could be well tolerated by the target proteins (Figure 1), and the synthesis of bromosporine was re‐designed in order to include the selected warhead and the clickable handle (see Supporting Information).

The ability of bromotriazine **2** (BTZ), to covalently modify BRDs characterized by the presence of a lysine residues on the variable BC loop, was tested by incubating the probe with recombinant BRDs in buffer at 37 °C. The formation of covalent adducts was detected by mass spectrometry (Figure 3). Different BRD sub‐families were used in a preliminary screen with the aim to define the covalent labelling potential of the newly synthesized probe: BRD4(1), BRD4(2), BRD3(2), BRD2(1), BRD2(2), TAF1(1), TAF1(2), TAF1L(1), BRD1, ATAD2 (Figure 3).

**Figure 3:**
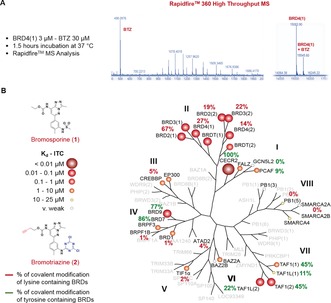
Bromotriazine covalent labelling across the bromodomain family tree. A) Bromotriazine **2**, a covalent probe inspired by the structure of bromosporine, a pan‐bromodomain inhibitor, was incubated with truncated BRDs belonging to different subfamilies. The degree of covalent modification was measured by RapidFire mass spectrometry, as shown for BRD4(1); B) Bromotriazine efficiently labels a wide range of BRDs belonging to different subfamilies by covalently reacting with non‐activated nucleophilic lysine and tyrosine residues on the rim of their binding site.

Moreover, a series of BRDs that did not contain a nucleophilic amino acid within reach were included in the study as controls: PB1(5), SMARCA2B, TIF1α, BRPF1B, and CREBBP. An accurate analysis of the data originated in this assay highlighted that the reactivity profile of BTZ (**2**) seemed to follow the activity profile observed with bromosporine, where higher affinity of the reversible probe corresponded to higher percentage of covalent modification by the irreversible probe, thus suggesting that the reversible binding played a pivotal role in driving the covalent labelling (Figure 3). In fact, BTZ efficiently labelled all the BETs (14–67 %) and was particularly effective against BRD2(1). At the same time, bromodomains that were weakly interacting with bromosporine (IC_50_≥1 μm) were only slightly modified (1–4 %) by BTZ, despite presenting a targetable lysine residue in the correct position (GCN5L2, ATAD2, BRD1). Negligible levels of modification (0 to 5 %) were detected in BRDs whose sequence suggested they could not be modified by BTZ since they lacked nucleophilic amino acids on the rim of their binding site.

To confirm the correct binding of bromotriazine within the KAc site and provide evidence for the preference of the reactive warhead for lysine residues, recombinant BRDs were incubated with the probe before undergoing tryptic digestion and LC‐MS/MS analysis.^20^, ^21^ In the case of BRD4(1) and BRD3(2), this approach confirmed that the preferred sites of modification were in fact the predicted lysine 91 in BRD4(1) and the corresponding residue, lysine 336, in BRD3(2) (Figure 4; see Supporting Information for complete data). These findings were compatible with the estimated binding mode of BTZ **2** in BRD4(1).

**Figure 4:**
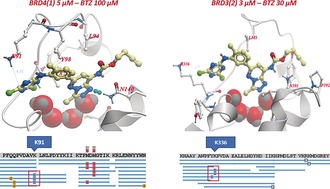
Mapping the sites of covalent modification via LC‐MS/MS. BTZ (**2**) was docked within the binding site of BRD4(1) (left) and then superimposed to the same pocket in BRD3(2) (right). Two lysines, K91 and K336, were found to be near the reactive dichlorotriazine warhead, thus suggesting they would be the expected site of covalent interaction. The predictions made by docking were subsequently confirmed by LC‐MS/MS peptide mapping.

Interestingly, the clearest covalent labelling was observed when BTZ was reacted with CECR2, a sub‐family I BRD characterized by the presence of a tyrosine residue in place of the corresponding lysine 91 in BRD4(1). Surprisingly, BRD9, a IV BRD sub‐family member, was covalently modified up to 77 % after 1.5 hours incubation at 37 °C, despite that it lacked a lysine near the KAc binding site. Since repeated attempts to modify BRD9 resulted in consistently high percentages of labelling but no reliable data could be produced by LC‐MS/MS peptide mapping, a crystallization experiment was set up in order to determine the exact site of interaction. The crystals obtained by seeding the purified fraction containing the modified protein showed a peculiar interlocked structure, in which one BTZ probe was located inside the binding site of one BRD, yet it was covalently linked to another tyrosine residue just outside of the binding pocket of another BRD in the crystallographic unit cell. (Figure 5—Top and bottom left) The resulting interlocked dimer is compatible with the previously reported 2:1 protein‐peptide stoichiometry of BRD9 functional complexes.^22^ Moreover, the superimposition of the crystal structures of BTZ and bromosporine in the binding pocket of BRD9 highlighted the high similarity of the two binding modes (Figure 5—Bottom right).

**Figure 5:**
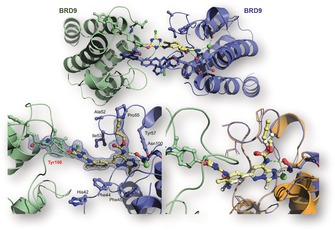
Mapping the sites of covalent modification via crystallographic techniques and crystal structure of the covalent complex formed by BTZ (**2**) and BRD9. The site of modification in this case was found to be a tyrosine (Tyr106) at the end of the AZ loop. The probe was reported to reversibly interact with the binding site of one BRD and form a covalent bond with the nucleophilic residue on an adjacent BRD, thus creating an interlocked dimer.

Based on this, we analysed all the BRDs amino acid sequences in order to identify those containing a tyrosine residue at the same position of the one targeted by BTZ **2** in BRD9 (Tyr106). Seven BRDs including PCAF, GCN5L2, TAF1 and TAF1L complied with this requirement, and they also contain a targetable lysine in the BC loop.

The reactivity of BTZ against the former two BRDs was limited (9 % and 0 %, respectively), consistent with the low affinity of the non‐covalent BSP for family I BRDs. However, labelling increased to 45 % against TAF1(1) and TAF1(2), reaching 86 % in the case of BRD7, a protein with high structural similarity to BRD9. These findings suggested that the presence of a tyrosine residue in proximity to the binding site could endow the corresponding BRD with higher reactivity against our probe when compared with lysine bearing BRDs.

To confirm this, we determined the kinetics of the covalent reaction between BTZ and BRDs, characterized by the presence of a targetable lysine (BRD4(1), BRD4(2), BRD3(2), BRD2(1)) or tyrosine (BRD9, CECR2) residue on the rim of the binding site.

The reaction between a protein and its irreversible inhibitor should follow a second order kinetic model.^23^ However, the order of the reaction was reduced by applying the isolation method,^24^ where each BRD (1 μm) was incubated with a high concentration of BTZ (100 μm) and the decrease of unmodified protein was monitored over time with the RapidFire MS system. Each experiment was conducted in triplicate, as reported in Figure 6 a for BRD4(1), and the average of the experimental values plotted as linear trends as shown in Figure 6 b (for additional data see Supporting Information—Section 5). Since the experimental conditions did not allow the discrimination between reversible and irreversible binding to the kinetics of the process, a cumulative constant (*k*_app_) was calculated for each reaction. Comparing the different *k*_app_ values confirmed the dramatic effect that changing the reactive amino acid had on the overall reaction rate (Figure 6 b). It should be highlighted that CECR2 (*k*_app_=12.6×10^−4^ s^−1^) is characterized by the presence of two tyrosine residues, one in a position corresponding to Lys91 in BRD4(1) and the other one in a position corresponding to Tyr106 in BRD9.

**Figure 6:**
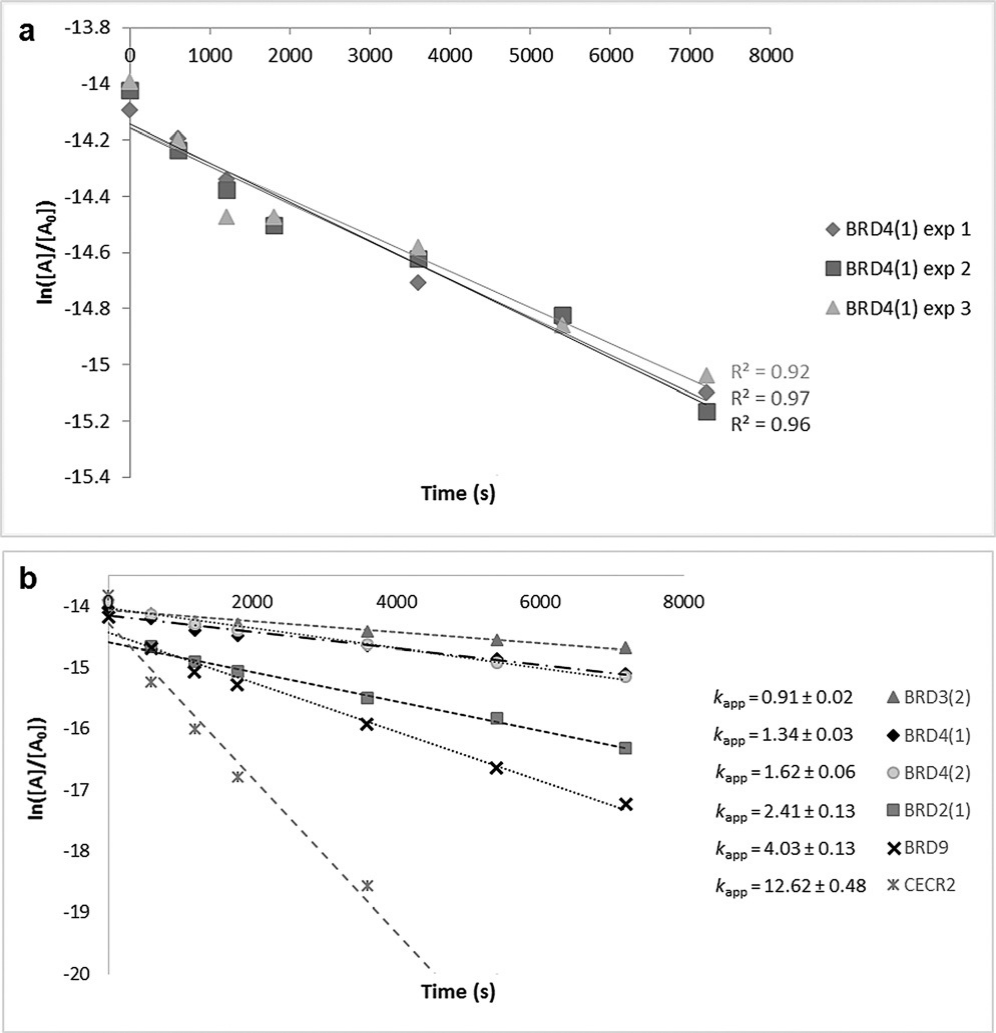
BTZ binding kinetics against BRDs belonging to different sub‐families a) BRD4(1) (1 μm) was incubated with BTZ (100 μm) and the amount of unmodified protein monitored over time as a function of the area (A) of the peak registered using a RapidFire low‐injection QToF mass spectrometer. The experiment was run in triplicate in order to determine the kinetic rate constant of the overall process. b) Comparing the linear graphs obtained in the previous kinetic experiments allowed us to rank the relative reactivity of lysine (BRD4(1), BRD4(2), BRD3(2) and BRD2(1)) and tyrosine (BRD9 and CECR2) containing BRDs, indicating preferential binding of BTZ to CECR2.

Taken together, we describe a broad‐spectrum probe that could covalently modify BRDs belonging to different sub‐families.

In addition, insightful information on the amino acid involved in the binding could be derived by co‐crystallization or LC‐MS/MS mapping.

To validate the applicability of BTZ (**2**) as a chemical proteomics tool, recombinant BRD4(1) was incubated with the covalent probe and the obtained covalent complex subjected to a copper‐catalyzed click chemistry reaction.^25^ When an azido‐dye (Cyanine 5.5‐IR dye) was used as the cycloaddition partner, the truncated protein appeared as a bright spot on polyacrylamide gel. When biotin azide was used in place of the azido‐dye, BRD4(1) was successfully immobilized on Streptavidin‐coated beads and then eluted from the resin by thermal denaturation. A covalent non‐clickable analogue of bromotriazine (**2 b**) was used in both experiments as a control since it lacked the alkyne functional handle necessary to react with the azide moiety on the dye or biotin tag (Figure 7).

**Figure 7:**
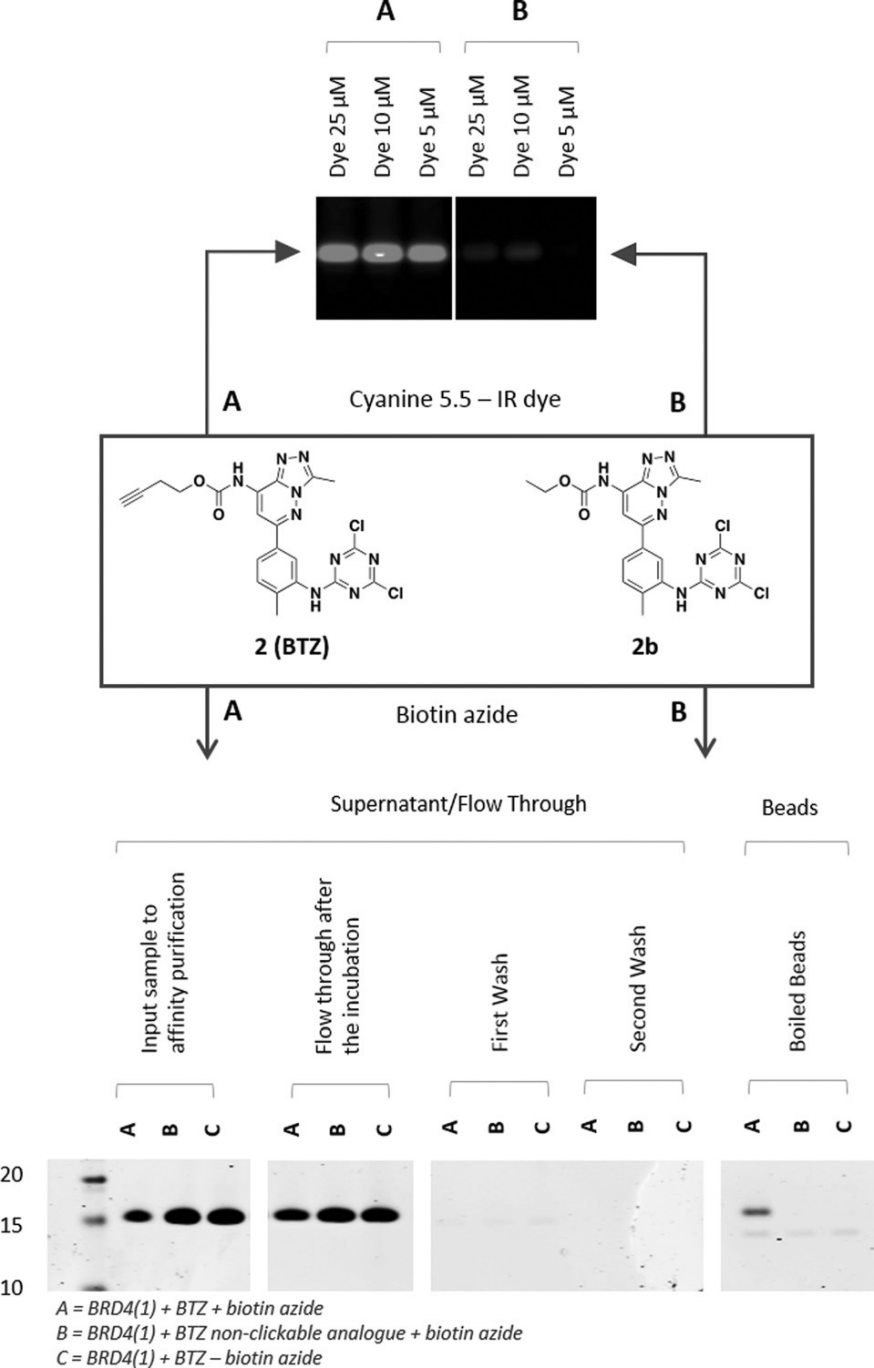
Fluorescent labelling and enrichment experiment conducted on recombinant BRD4(1) using BTZ (**2**) and its non‐clickable analogue (**2 b**) Recombinant BRD4(1) (1 mg mL^−1^) was incubated with BTZ (100 μm) or its non‐clickable analogue **2 b** (100 μm) for 1.5 hours at 37 °C. The obtained samples were reacted with an azido‐dye (Cyanine 5.5) (top) or biotin‐azide (bottom) under copper‐catalyzed alkyne azide cycloaddition (CuAAC) conditions, in order to selectively visualize the formed BRD4(1)‐BTZ (**2**) covalent adduct under IR irradiation (top) or to pull down the obtained complex on Streptavidin beads (bottom).

Encouraged by these in vitro results, chemical proteomics experiments were conducted in K‐562 chronic myelogenous leukemia cell line and in THP‐1 monocytic cell line, as they both express BRD4 and BRD2 at a high endogenous level.^26^ Promising results were obtained in particular after pull down experiments were conducted on the THP‐1 cell line, where whole cell lysates were incubated with increasing concentrations of probe **2** (10, 25 and 100 μm) at 4 °C and samples were collected at 1 h, 4 h, 8 h, and 24 h time points; an additional time point was taken after 5 minutes of incubation to highlight any unspecific pull down that could be ascribed to the probe's intrinsic reactivity (see Supporting Information).

The analysis of the resulting protein pull‐down on NeutrAvidin beads by silver staining and Western blotting highlighted a direct correlation between the total amount of pull down, the incubation time, and the concentration of the probes.

In a series of subsequent pull‐down experiments, the formation of BRD‐BTZ (**2**) covalent adducts was competed by preincubation of THP‐1 whole cell lysates with 100 μm BTZ non‐clickable analogue (**2 b**) or 100 μm JQ1(+), a known BET bromodomains inhibitor.^27^ Although the MS/MS analysis of the competition experiments did not show a significant enrichment of BRDs pull down in the non‐competed samples, possibly because of the generally low levels of BRD pull down at low temperatures, these experiments highlighted a series of potentially interesting BTZ off‐targets. In particular, Thymosin beta‐4 (TMSB4X), a protein involved in cell proliferation and migration identified as a biomarker of malignancy in solid tumors,^28^ was enriched by treatment with BTZ (**2**) and selectively competed by pre‐treatment with both **2 b** and JQ1(+), thus suggesting that BTZ (**2**) selectively bound and labeled this protein (see Supporting Information).

The initial attempts to selectively visualize the covalent BRD‐BTZ adducts formed after incubation of K‐562 cell lysates with BTZ (**2**) using Cy 5.5 dye proved to be extremely challenging, possibly due to the promiscuity of the probe (see Supporting Information).

However, the MS/MS analysis conducted on pull‐down samples obtained by incubating non‐transfected K‐562 cell lysates for 1 hour at 37 °C with 5, 50, and 100 μm BTZ (**2**), respectively (see Supporting Information for complete data), and then appending a biotin handle on the formed covalent complexes by CuAAC, showed a significant enrichment of BRD4, BRD8 and BRD associated proteins (SMARCA4, SUPT16H) in the higher concentration samples, compared to the lowest concentration one (Figure 8). The identification of BRD8 is of particular note as BRD8 has never been produced recombinantly and although BTZ (**2**) was not tested for binding BRD8 in vitro, its enrichment in cell lysate shows that BTZ is potentially a BRD8 inhibitor.

**Figure 8:**
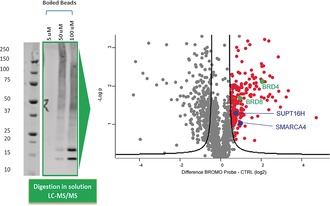
MS/MS proteomics analysis of enrichment experiments in non‐transfected K‐562 cells using BTZ (**2**) Extracts prepared from K‐562 cells were incubated with BTZ (**2**) at 5, 50, and 100 μm BTZ (**2**), respectively, for 1 h at 37 °C, followed by in solution trypsin digestion, and analysis by LC‐MS/MS. A volcano plot analysis shows the enrichment of proteins by the BTZ (**2**) probe (marked as red dots) that includes BRD4 (Uniprot nr 060885) and BRD8 (Uniprot nr H7C127), both indicated in green, and known BRD interactors (blue).

In conclusion, we designed and synthesized a covalent probe, BTZ (**2**), against bromodomain containing proteins. Our results highlight the feasibility of selectively targeting non‐catalytic amino acids on the rim of the ligand binding site by correctly orienting the chosen electrophilic moiety towards the desired nucleophilic residue, as recently reported by Zhao et al. in the case of protein kinases.^29^ In particular, an unprecedented selective labelling of non‐catalytic and highly conserved lysines of purified bromodomains was reported. We also discovered the previously unreported ability of a 2,4‐dichlorotriazine electrophile to label tyrosine residues and the surprising dimerization process induced by such covalent interaction with BRD9 that broadens the prospective for future design of covalent and selective inhibitors. This may apply not only to the bromodomain family, but to alternative cellular targets characterized by the presence of a lysine and/or tyrosine residue in the proximity of the binding site.

## Conflict of interest

The authors declare no conflict of interest.

## Supporting information

As a service to our authors and readers, this journal provides supporting information supplied by the authors. Such materials are peer reviewed and may be re‐organized for online delivery, but are not copy‐edited or typeset. Technical support issues arising from supporting information (other than missing files) should be addressed to the authors.

Supplementary
