# Supplementary material for: An Activity‐Based Probe Targeting Non‐Catalytic, Highly Conserved Amino Acid Residues within Bromodomains
Source: Angew Chem Int Ed Engl. 2018 Dec 27;58(4):1007–12. doi: 10.1002/anie.201807825 (PMC6492141; doi:10.1002/anie.201807825)

Angewandte Chemie
Eine Zeitschrift der Gesellschaft Deutscher Chemiker

Supporting Information

An Activity-Based Probe Targeting Non-Catalytic, Highly Conserved Amino Acid Residues within Bromodomains

Melissa D'Ascenzio, Kathryn M. Pugh, Rebecca Konietzny, Georgina Berridge, Cynthia Tallant, Shaima Hashem, Octovia Monteiro, Jason R. Thomas, Markus Schirle, Stefan Knapp, Brian Marsden, Oleg Fedorov, Chas Bountra, Benedikt M. Kessler, and Paul E. Brennan**

anie_201807825_sm_miscellaneous_information.pdf

Supporting Information

1. Chemical Proteomics Experiments 2
1.1 *In gel* visualization of recombinant BRD4(1) using Cy 5.5 IR dye. 2
1.2 Pull down of recombinant BRD4(1) using Biotin-azide. 3
1.3 Attempted *in gel* visualization of BRD4 in non transfected K-562 cell lysates using Cy 5.5 IR dye. 4
1.4 Pull down of BRD4 from non-transfected K-562 cell lysates 5
Western Blot – Anti-BRD4 and Anti-Biotin monoclonal antibodies *6*
Sample Preparation for mass spectrometry analysis *7*
LC-MS/MS analysis of enriched K-562 cell lysates *7*
1.5 Pull down of BRDs from non-transfected THP-1 cell lysates 8
Optimised procedure for chemical proteomics experiments: *8*
Time and Concentration Screening – WB BRD4 .... *9*
Time and Concentration Screening – WB BRD2 .... *9*
1.6 Quantitative Chemical Proteomics on THP-1 cell lysates 10

2. Crystallographic Data 12
2.1 Materials and Methods 12
Cloning, protein expression and purification of bromodomains *12*
2.2 Crystallographic data collection and refinement statistics 13

3. Docking Data 14
3.1 BTZ (2) and BRD4(1) 14

4. Synthetic Procedures and NMR Spectra 15
Synthetic Scheme A 15
Synthetic Scheme B 16
3,6-Dichloropyridazin-4-amine (4) 17
6-Chloro-3-hydrazinylpyridazin-4-amine (5) 17
6-Chloro-3-methyl-[1,2,4]triazolo[4,3-*b*]pyridazin-8-amine (6) 17
Tert-butyl (6-chloro-3-methyl-[1,2,4]triazolo[4,3-*b*]pyridazin-8-yl)carbamate (7) 17
Tert-butyl (3-methyl-6-(4-methyl-3-nitrophenyl)-[1,2,4]triazolo[4,3-*b*]pyridazin-8-yl) carbamate (9) 18
But-3-yn-1-yl carbonochloridate (11) 18
But-3-yn-1-yl (6-(3-nitro-4-methylphenyl)-3-methyl-[1,2,4]triazolo[4,3-*b*]pyridazin-8-yl)carbamate (12) 19
But-3-yn-1-yl (6-(3-amino-4-methylphenyl)-3-methyl-[1,2,4]triazolo[4,3-*b*]pyridazin-8-yl)carbamate (13) 19
But-3-yn-1-yl (6-(3-((4,6-dichloro-1,3,5-triazin-2-yl) amino)-4-methylphenyl)-3-methyl-[1,2,4] triazolo [4,3-*b*]pyridazin-8-yl) carbamate hydrochloride (BTZ-2) 20
Ethyl (6-(3-nitro-4-methylphenyl)-3-methyl-[1,2,4]triazolo[4,3-*b*]pyridazin-8-yl)carbamate (15) 20
Ethyl (6-(3-amino-4-methylphenyl)-3-methyl-[1,2,4]triazolo[4,3-*b*]pyridazin-8-yl)carbamate (16) 21
Ethyl (6-(3-((4,6-dichloro-1,3,5-triazin-2-yl)amino)-4-methylphenyl)-3-methyl-[1,2,4]triazolo[4,3-*b*]pyridazin-8-yl)carbamate hydrochloride (2b) 21

NMR Spectra 22

5. Rapidfire Mass Spectrometry 48
5.1 Recombinant Bromodomains Sequences 49
5.2 Kinetic Experiments: 51
5.2.1 BTZ 100 µM and BRD4(1) 1 µM *51*
5.2.2 BTZ 100 µM and BRD4(2) 1 µM *53*
5.2.3 BTZ 100 µM and BRD3(2) 1 µM *55*
5.2.4 BTZ 100 µM and BRD2(1) 1 µM *57*
5.2.5 BTZ 100 µM and BRD9 1 µM *59*
5.2.6 BTZ 100 µM and CECR2 1 µM *61*

6. LC-MS/MS Analysis for Peptide Identification 63
6.1 BTZ & BRD4(1) – Lysine 91 64
6.2 BTZ & BRD3(2) – Lysine 336 65

1. Chemical Proteomics Experiments

Unless otherwise specified, reagents, solvents, buffers and kits were purchased from Bio-Rad®, Sigma-Aldrich®, Thermofischer®, Lumiprobe® and used without any further purification and/or alteration. The reported procedures were adapted from previously published protocols.[1-2]

1.1 *In gel* visualization of recombinant BRD4(1) using Cy 5.5 IR dye.

Sample A: 3.8 µL of a 20 µM solution of Bromotriazine (**2**) in DMSO were added to 146 µL of a solution of the truncated form of BRD4(1) (1.0 mg/mL) in PBS to a final concentration of 0.5 µM.
Sample B: 3.8 µL of a 20 µM solution of BTZ-Non clickable analogue (**2b**) in DMSO were added to 146 µL of a solution of the truncated form of BRD4(1) (1.0 mg/mL) in PBS to a final concentration of 0.5 µM.
Samples A and B were incubated in an Eppendorf Thermomixer™ at 37°C shaking at 300 rpm for 1.5 hours. A 5 µL aliquot was collected from each sample, diluted to 10 µL and stored at -80°C. The remaining samples were then transferred into new tubes in 40 µL aliquots for the click reaction step. 1 µL of a 1.25 mM solution of Cy5.5 azido-dye (a molecule endowed with far red/ near infrared emission) in PBS was added to samples Acetone P, A1 and B1 to a final dye concentration of approximately 25 µM. 1 µL of a 0.5 mM solution of Cy5.5 azido-dye in PBS was added to samples A2 and B2 to a final dye concentration of approximately 10 µM, and 1 µL of a 0.25 mM solution of Cy5.5 azido-dye in PBS was added to samples A3 and B3, to a final dye concentration of approximatively 5 µM. 5 µL of 10% SDS were added and the solution mixed by vortexing before adding 5 µL of the catalyst mix containing 3 parts of TBTA (1.7 mM), 1 part of copper sulfate (50 mM) and 1 part of TCEP (50 mM). The obtained samples were incubated for 1.5 hour at 37°C shaking at 300 rpm. 0.5 µL of a 500 mM solution of EDTA were added and the samples precipitated by the addition of cold acetone (Acetone P) or by chloroform/methanol extraction (A1-A3 and B1-B3). The dried precipitated pellets were resuspended in 30 µL of 10%SDS and run on a polyacrylamide gel. The fluorescent gel was visualized using an Odyssey® CLx IR scanner (**Supplemental Figure 1**) and the protein content confirmed by Coomassie Brilliant Blue staining (**Supplemental Figure 2**).

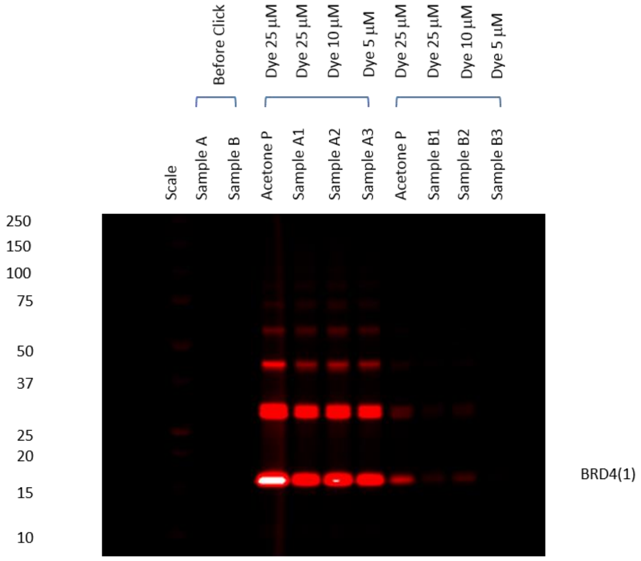

Supplemental Figure 1. LI-COR assisted visualisation of the recombinant BRD4(1)-BTZ(**2**) complex tagged with CY 5.5 IR dye.

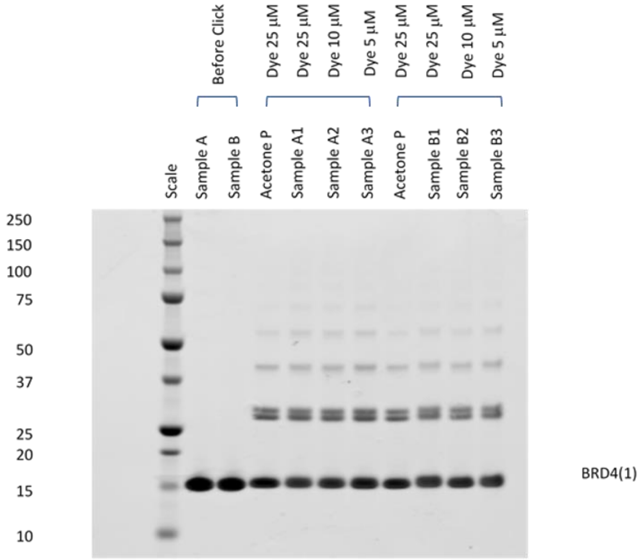

Supplemental Figure 2. Coomassie staining of the gel reported in **Supplemental figure 1**

(1) Mackinnon, A. L. & Taunton, J. Target Identification by Diazirine Photo-Cross-linking and Click Chemistry. *Curr. Protoc. Chem. Biol.* **2009**, *1*, 55-73.
(2) Hela, P. W.; Wright, M. H.; Thinon, E. & Tate E. W. Multifunctional protein labeling via enzymatic N-terminal tagging and elaboration by click chemistry. *Nature Protocols* **2012**, *7*, 105-107

1.2 Pull down of recombinant BRD4(1) using Biotin-azide.

Sample A: 12.5 µL of a 200 µM solution of Bromotriazine (**2**) in DMSO were added to 475 µL of a solution of the truncated form of BRD4(1) (0.5 mg/mL) in PBS to a final concentration of 5 µM.
Sample B: 12.5 µL of a 200 µM solution of BTZ-Non clickable analogue (**2b**) in DMSO were added to 475 µL of a solution of the truncated form of BRD4(1) (0.5 mg/mL) in PBS to a final concentration of 5 µM.
Sample C was treated as sample A until the click chemistry step.
Samples A-C were incubated in an Eppendorf Thermomixer^{TM} at 37°C shaking at 300 rpm for 1.5 hours. The proteins were precipitated by incubating the samples in acetone for 2 hours at -26°C and the obtained pellets re-dissolved in 97.5 µL of PBS and 12.5 µL of 10% SDS *via* sonication. 2.5 µL of a 50 mM solution of Biotin-azide in PBS were added to Samples A and B, while only PBS was added to sample C. 12.5 µL of the catalyst mix containing 3 parts of TBTA (17 mM), 1 part of copper sulfate (500 mM) and 1 part of TCEP (500 mM) was added to each sample and the reaction incubated for 30 minutes at 37°C shaking at 300 rpm. 5 µL aliquot was removed at this point, 1 µL of Laemmli 4x was added and the aliquots were snap frozen and kept at -80°C (**Input Samples to Affinity Precipitation**). The remaining samples were precipitated by adding 1 mL of cold acetone to every sample and incubating the resulting suspension at -26°C overnight. The precipitated sediment was spun at 20000 x g for 10 minutes at 4°C, the supernatant was removed by aspiration and 0.5 mL of cold acetone were added. The samples were sonicated to break up and disperse the precipitated proteins and then returned to -80°C for 10 minutes. The supernatant was removed and the obtained pellets dried at the air before being redissolved in 50 µL of 1% SDS in PBS *via* sonication. The obtained solutions were diluted up by adding 0.5 mL of affinity purification buffer (50 mM HEPES, 100 mM NaCl, 1% NP-40). 20 µL aliquots were removed from each samples, added of 4 µL of Laemmli 4x and kept at -80°C (**Efficiency of Precipitation and Resolubilization**). 50 µL of a slurry of Streptavidin coated magnetic beads was added to each sample and they obtained suspension shaken at 700 rpm at room temperature for 2 hours. The beads were sedimented using a magnetic tube-holders, the supernatant was removed and 20 µL aliquots were kept at -80°C to be run on the gel as **Flow Through After Incubation with Beads**. The beads were washed twice (50 mM HEPES, 500 mM NaCl, 1% NP-40 in PBS) and eluted first using 50 µL of a 2 mM solution of free Biotin in 1 % NP-40 in PBS and then by boiling the streptavidin off at 90°C for 5 minutes. The samples were run on a pre-packed 4-12% polyacrylamide gel and stained using Coomassie Brilliant Blue staining solution.

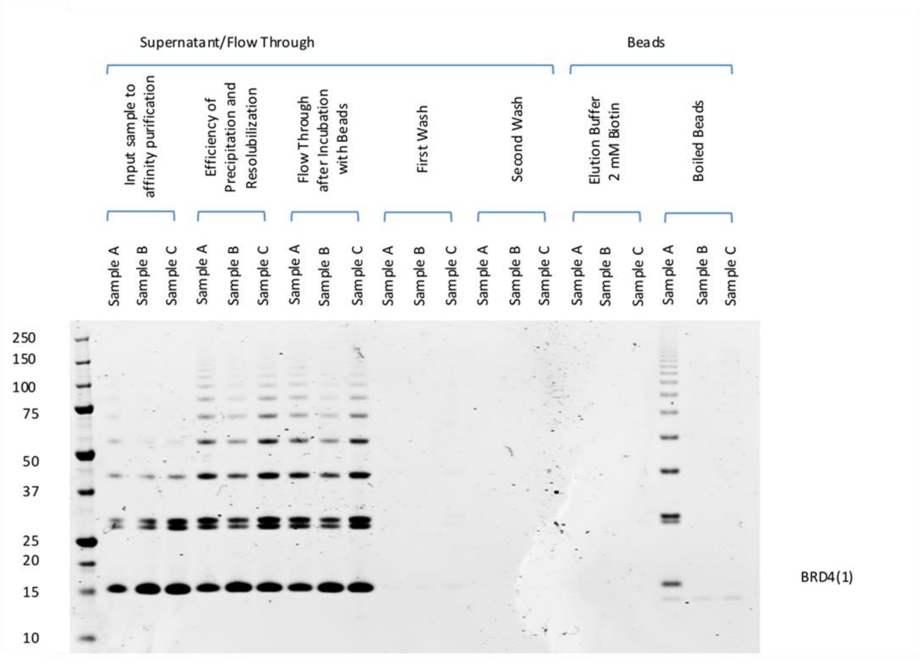

Samples A, B and C were respectively:

A = Incubation with Bromotriazine (BTZ - **2**) and CuAAC with azido-biotin

B = Incubation with Bromotriazine analogue without the clickable handle (**2b**) and CuAAC with azido-biotin

C = Incubation with Bromotriazine (1) and CuAAC without azido-biotin.

Supplemental Figure 3. Coomassie staining of the gel obtained from the pull down of recombinant BRD4 covalently bond to BTZ(**2**) using Streptavidin coated magnetic beads. The extra bands appearing at higher molecular weights than the pure recombinant protein (15083 Da) were due to protein aggregation and precipitation induced after the incubation with the click reagents. In fact, LC-MS/MS analysis confirmed that all bands corresponded to recombinant BRD4(1).

1.3 Attempted *in gel* visualization of BRD4 in non transfected K-562 cell lysates using Cy 5.5 IR dye.

Sample A: 5 µL of a 20 µM solution of Bromotriazine (**1**) in DMSO were added to 195 µL of K-562 cell-lysates (1.0 mg/mL) in LBII (HEPES 50 mM, 10% glycerol, KCl 500 mM, TCEP 1 mM, NP40 1%, Roche® Protease Inhibitor cocktail), to a final concentration of 0.5 µM.
Sample B: 5 µL of a 20 µM solution of BTZ-Non clickable analogue (**2b**) in DMSO were added to 195 µL K-562 cell-lysates (1.0 mg/mL) in LBII, to a final concentration of 0.5 uM.
Samples A and B were incubated in an Eppendorf Thermomixer^{TM} at 37°C shaking at 300 rpm for 1.5 hours. A 5 µL aliquot was collected from each sample, diluted to 20 µL and stored at -80°C (**Protein before Incubation**). 180 µL of both sample A and sample B were then transferred into new tubes for the click reaction step. Addition of 10% SDS was avoided at this point since it induced protein precipitation in LBII. 4 µL of a 0.25 mM solution of Cy5.5 azido-dye in PBS were added to both samples to a final dye concentration of approximately 5 µM. 20 µL of the catalyst mix containing 3 parts of TBTA (1.7 mM), 1 part of copper sulfate (50 mM) and 1 part of TCEP (50 mM) were added and the obtained samples were incubated for 1 hour at 37°C shaking at 300 rpm. 0.9 µL of a 5 mM solution of EDTA were added to half of the samples. Protein precipitation was performed in cold acetone or by chloroform/methanol extraction. The dried precipitated pellets were resuspended in 20 µL of 10%SDS and diluted to 80 µL with PBS. To half of the 15 µL aliquots for the gel, 1 µL of DTT was added and the obtained gel visualized using an Odyssey® CLx IR scanner.

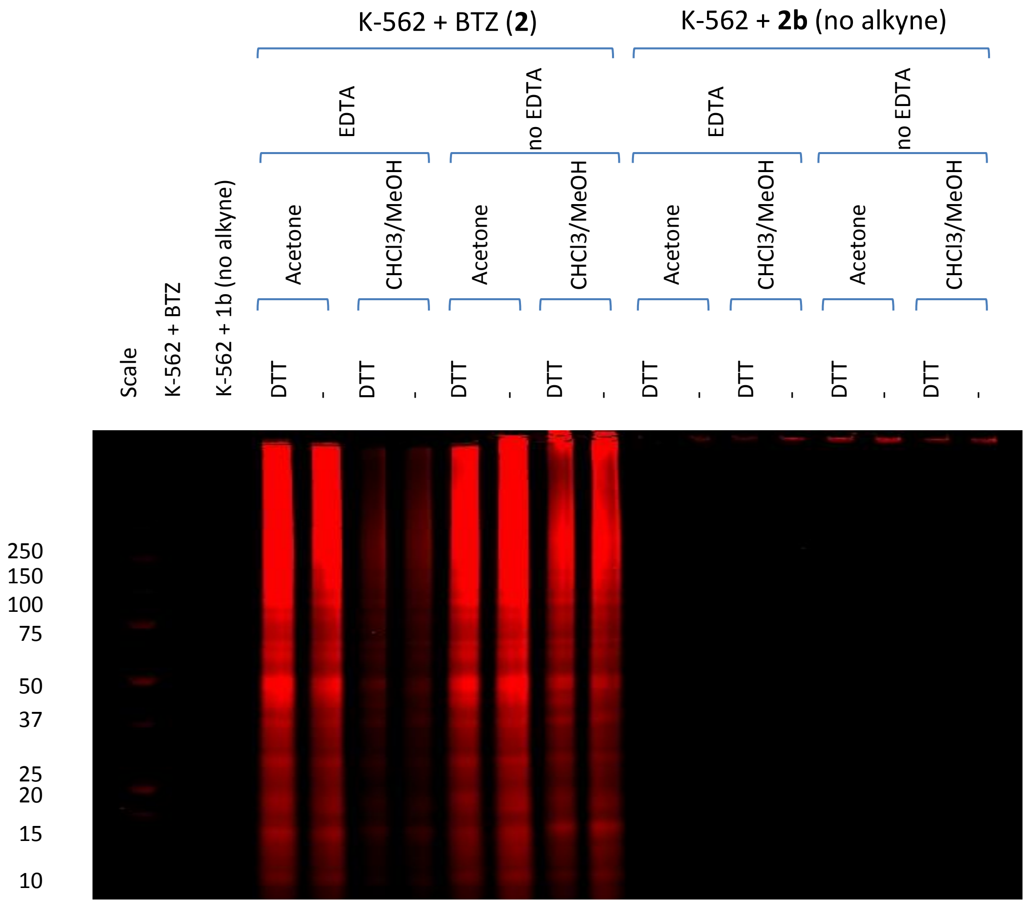

Supplemental Figure 4. LI-COR scan of K-562 cell lysates treated with BTZ (2) and its non clickable analogue **2b**. The gel showed selective labelling of samples incubated with the clickable compound, BTZ (**2**), over those incubated with compound **2b**. However, the formation of smears and the evenly spread IR fluorescence indicated non-selective and unspecific binding of BTZ (**2**) to an extensive portion of the proteome.

1.4 Pull down of BRD4 from non-transfected K-562 cell lysates

Three different concentrations of Bromotriazine (**2**) were incubated with K-562 lysates (5mg/mL) in order to qualitatively and quantitatively study the changes protein pull down with the increasing concentration of probe.
Sample A1: 10 µL of a 100 µM solution of Bromotriazine (**2**) in PBS (0.5% DMSO) were added to 200 µL of K-562 cell lysates (5 mg/mL) in LBII to a final concentration of 5 µM.
Sample A2: 10 µL of a 1 mM solution of Bromotriazine (**2**) in PBS (5% DMSO) were added to 200 µL of K-562 cell lysates (5 mg/mL) in LBII to a final concentration of 50 µM.
Sample A3: 10 µL of a 2 mM solution of Bromotriazine (**2**) in PBS (20% DMSO) were added to 200 µL of K-562 cell lysates (5 mg/mL) in LBII to a final concentration of 100 µM.
Control: 10 µL of a 100 µM solution of Bromotriazine (**2**) in PBS (0.5% DMSO) were added to 200 µL of K-562 cell lysates (5 mg/mL) in LBII to a final concentration of 5 µM.
Samples A1-A3 were incubated in an Eppendorf Thermomixer^{TM} at 37°C shaking at 300 rpm for 1.5 hours. 10 µL aliquots were taken for the final gel run (**Incubation with Bromotriazine**). The lysates were divided into 100 µL aliquots and precipitated by adding cold acetone and incubating the samples for 2 hours at -26°C. The obtained pellets were re-dissolved in 20 µL of 10% SDS *via* sonication and diluted to 110 µL with PBS. 5 µL samples were taken from each tube to give a final volume of 10 µL for each concentration (**Efficiency of Precipitation and Resolubilization**). 2.5 µL of a 50 mM solution of Biotin-azide in PBS and 12.5 µL of the catalyst mix containing 3 parts of TBTA (17 mM), 1 part of copper sulfate (500 mM) and 1 part of TCEP (500 mM) were added to each sample, except from the control sample. The reactions were incubated for 1 hour shaking at 300 rpm, at 37 °C and room temperature respectively. 10 µL aliquots were taken at this point, 1 µL of Laemmli 4x was added and the aliquots snap frozen and kept at -80°C (**Input Samples to Affinity Precipitation**). The remaining samples were precipitated by adding 1 mL of cold acetone to each sample and incubating the resulting suspension at -26°C overnight. The precipitated sediment was spun at 20000 x g for 10 minutes at 4°C, the supernatant was removed by aspiration and 1 mL of cold acetone was added. Samples were sonicated to break up the pellets and disperse the precipitated proteins, they were then returned to -80°C for 10 minutes. The supernatant was removed and the obtained pellets dried at the air before being redissolved in 10 µL of 10% SDS and 10 10 µL of PBS *via* sonication. The obtained solutions were diluted up by adding 0.5 mL in affinity purification buffer (50 mM HEPES, 100 mM NaCl, 1% NP-40). 20 µL aliquots were removed from each samples, 4 µL of Laemmli 4x were added and kept at -80°C (**Efficiency of 2nd Precipitation and Resolubilization**). 175 µL of a slur of Streptavidin coated magnetic beads in affinity purification buffer was added to each sample and the obtained suspension incubated at 4 °C for 2 hours The beads were sedimented using a magnetic tube-holders, the supernatant was removed and 20 µL aliquots were kept at -80°C to be run on the gel as **Flow Through After Incubation with Beads**. The beads were washed twice (50 mM HEPES, 500 mM NaCl, 1% NP-40 in PBS) and eluted by boiling the streptavidin off in Laemmli buffer at 90°C for 5 minutes. The samples were run on a pre-packed 4-12% polyacrylamide gel. The proteins were transferred on a membrane and blocked with 5-6% blocking agent in PBS for 15 minutes. The membrane was then incubated with an in house prepared anti-BRD4 (mouse) antibody at 4 °C (1/500), washed twice with PBST and incubated with a secondary antibody bearing an excitable Li-COR sensitive dye. Unfortunately, no band corresponding to full length BRD4 could be detected and even the low molecular weight bands at around 80 KDa, which could be truncated forms of BRD4, seemed to disappear after the click reaction step (**Supplemental Figure 5**).
However, stripping the original membrane and blotting it against an anti-Biotin antibody demonstrated the efficiency of the click reaction. In this case, the amount of pull down on the beads was proportional to the concentration of Bromotriazine (**2**), despite no characteristic band corresponding to BRD4 could be identified (**Supplemental Figure 5**).

Western Blot – Anti-BRD4 and Anti-Biotin monoclonal antibodies:

- K-562 5 mg/mL (100 uL)

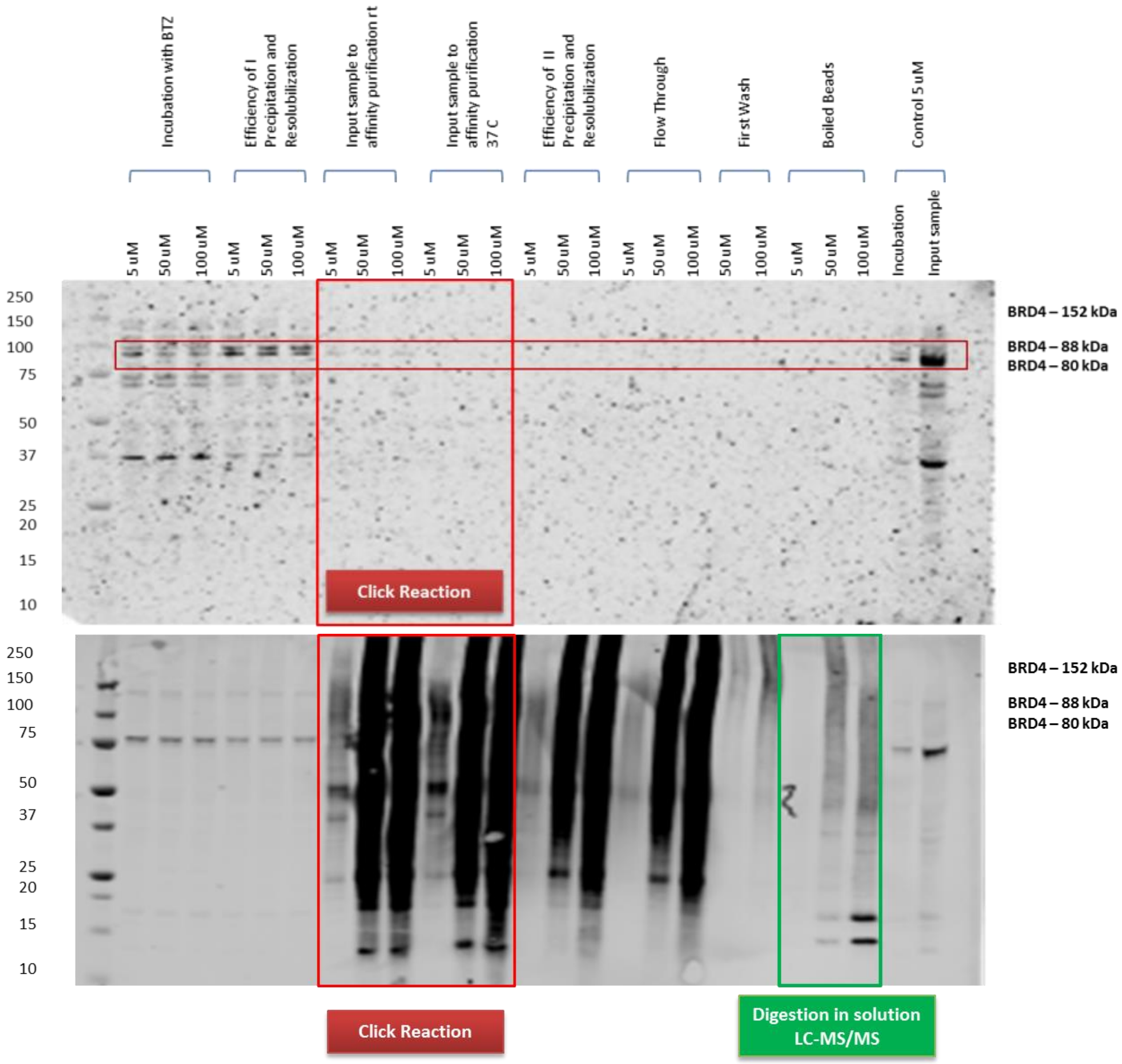

Supplemental Figure 5. First attempt to pull down BRD4 from non-transfected K-562 cell lysates using BTZ(**2**). The top membrane was incubated with anti-BRD4 antibodies and visualised using a secondary antibody bearing an excitable Li-COR sensitive dye. Since no BRD4 could be detected after the click reaction step, the membrane was subsequently stripped and blotted against biotin to investigate the efficiency of the click reaction – bottom membrane.

The proteomics analysis of the protein boiled off the beads, especially at 50 and 100 µM probe (**2**) concentration highlighted that almost 30% of pulled down proteins were nuclear proteins.

Sample Preparation for mass spectrometry analysis

After incubation of K-562 lysates with 100 µM, 50 µM, or 5µM BTZ (**2**), a biotin handle was appended to the covalent BTZ-target proteins adducts by CuAAC. Pull down of the covalent complexes was performed on Streptavidin-coated magnetic beads and the enriched extracts were purified by filtering the sample through a Micro Bio-Spin® column (Bio-Rad). For in-solution digestions, trypsin from bovine pancreas (1 µg/µL in 0.1 HCl, Promega) was reconstituted in 800 µL of 50 mM ammonium bicarbonate, and 4 µL of this solution were added to 100 µL of the 3 µM solution of BRD (+ probe). The solution was incubated overnight at 37 ºC. After the digestion was complete, the obtained peptides were purified by Sep-PAK C-18 solid-phase extraction according to the manufacturer's instructions (Waters) and concentrated under vacuum and kept at -20 ºC until analysis.

LC-MS/MS analysis of enriched K-562 cell lysates

Samples were analysed on a liquid chromatography tandem mass spectrometry (LC-MS/MS) essentially as described previously.[3] In brief, resulting peptides were analysed on an Acquity nano UPLC system (Waters) supplemented with a 25 cm C18 column, 1.7 µm particle size (Waters) online coupled to an LTQ Orbitrap Elite (Thermo Scientific). Peptides were eluted by applying a 60 min linear gradient from 5% buffer A (0.1% formic acid in water) to 40% buffer B (0.1% formic acid in acetonitrile) at a flowrate of 250 nl/min (approx. 6000 psi). Full MS scans were performed at a resolution of 30.000. Collision-induced dissociation was performed on the twenty most abundant ions per full MS scan using an isolation width of 1.0 Da. Fragment ions were acquired at a resolution of 7.500. All fragmented precursor ions were actively excluded from repeated MS/MS analysis for 15 s.

Data processing and analysis

Processing of MS Data RAW files were imported into Progenesis QIP (v 3.0.6039.34628), and MS/MS spectra exported as mascot generic files (.mgf) files using the 200 most intense peaks without deconvolution for searching. Datasets were searched in Mascot (v2.5.1) using the following parameters: 10 ppm precursor mass accuracy, 0.05 Da fragment mass accuracy, Oxidation (M), Deamidation (N, Q) and Propionamide (K) as variable modifications, Propionamide (C) as a fixed modification, and two missed cleavage sites. We applied 1% FDR at peptide level (both search engines use a target-decoy method for FDR estimation) and an additional Mascot ion score cut-off of 20 before importing search results into Progenesis, where protein quantification was calculated using the Top3 method. We used Perseus software (v 1.6.0.2) to identify BTZ2 enriched proteins applying a student t test, and p values were corrected for multiple testing using permutation FDR with default settings in Perseus. The data was plotted as a volcano plot (Figure 8) using 0.05 FDR and 0.1 S0.

(3) Adam, J.; Hatipoglu, E.; O'Flaherty, L.; Ternette, N.; Sahgal, N.; Lockstone, H. *et al.* Renal Cyst Formation in Fh1-Deficient Mice Is Independent of the Hif/Phd Pathway: Roles for Fumarate in KEAP1 Succination and Nrf2 Signaling. *Cancer Cell.* **2011**, *20*(4), 524–537.

1.5 Pull down of BRDs from non-transfected THP-1 cell lysates

Optimised procedure for chemical proteomics experiments:

THP-1 lysates were used in the proteomics experiments, since they constitutively express significant amounts of both BRD4 and BRD2. 30 mg of lysate was diluted to a final concentration of 4 mg/mL in base buffer (50 mM HEPES, 150 mM NaCl, 15 mM MgCl and 5% Glycerol) and spun down at 18000rcf for 20 minutes at 4 °C. The supernatant was separated from the precipitate, Halt^{TM} cocktail inhibitor and 10% NP40 (to final concentration of 0.4%) were added, together with the appropriate amount of compound **2** (in DMSO) to reach a final concentration of 10, 25 and 100 µM, respectively. The obtained samples were incubated at 4 °C up to 16 hours. 1 mL aliquots were taken from each solution at specific time points: 5 min, 1h, 4h, 8h, and 16h, snap frozen and preserved overnight. After the samples were gently thawed, 10% SDS was added to each tube to a final concentration of 1%SDS and the content of each tube vigorously mixed in order to denature the proteins and expose the alkyne moiety of the covalently bound probes. 25 µL of biotin azide 5M stock in DMSO was added, followed by 25 µL of the catalyst mix, which was previously prepared by adding 15 µL TBTA (1.7 mM), 50 µL of TCEP (50 mM), and 50 µL of copper sulfate (50 mM) to 0.135 mL of a 4:1 *t*-Butanol:DMSO solution. The obtained light green solutions were incubated at 37 °C for 2 hours in an Eppendorf ThermoMixer®. The protein content was precipitated on cold acetone overnight, centrifuged at 1250 rcf for 10 min at 4 °C, and the obtained pellets reconstituted in 150 µL of 1% SDS in base buffer. The complete re-dissolution of the proteins contained in the pellets was obtained by probe sonication (20 seconds cycles – 50% amplitude), after which the concentration of each sample was standardised by Pierce^{TM} 660nm Protein Assay. An adequate amount of lysates, calculated in order to keep the total amount of sample submitted to the following step constant, was added to a slurry containing 50 mg of Agarose beads previously reconstituted in base buffer and 1%SDS. Samples were incubated overnight on a rocking platform at room temperature. The beads were then loaded on Mobicols and washed twice with 10% NP40 and 0.1% DTT in base buffer, followed by a single wash with 0.1% DTT. The excess buffer was removed from the columns by gentle centrifuge spin and the pull down proteins eluted in 72 µL of 2x LDS and 10 mM DTT by heating the samples at 55 °C for 30 minutes in an Eppendorf ThermoMixer®. 5 µL of a iodoacetamide (IAA) stock solution (200 mg/mL) was added and each sample run on three separate polyacrylamide gel, which were then submitted to silver staining (**Supplemental Figure 6**), following to the procedure reported in the Pierce^{TM} Silver Stain Kit, and Western Blotting using anti-BRD4 (CSL BRD4 (E2A7X) Rabbit mAb #13440) and anti-BRD2 (CSL BRD2 (D89B4) Rabbit mAb #5848) monoclonal antibodies, respectively. Both WB where developed on photographic film after incubation with HRP-labelled secondary antibody (**Supplemental Figures 7-8**).

Time and Concentration Screening – Silver Staining

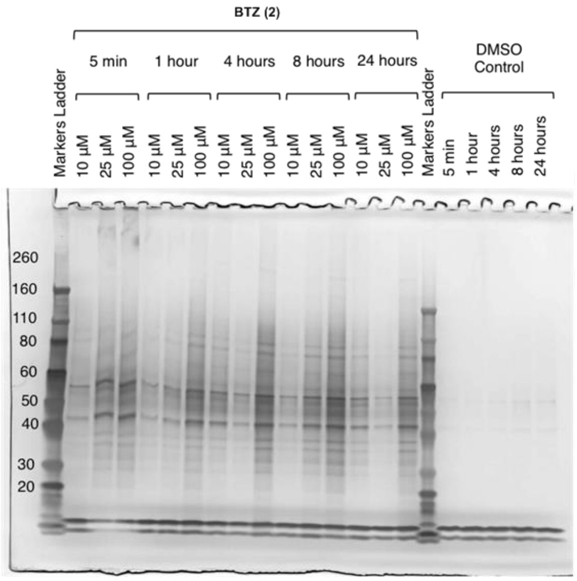

Supplemental Figure 6. Silver staining of the gels obtained by incubating THP-1 cell lysates with cmpd (**2**)

Time and Concentration Screening – WB BRD4

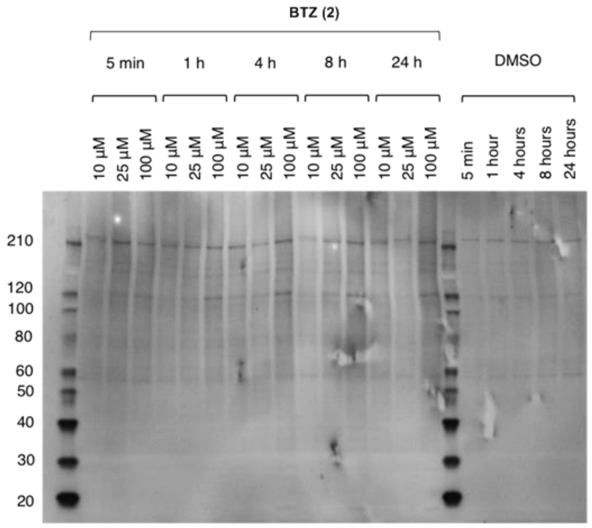

Supplemental Figure 7. WB developed on photographic film of BRD4 pull down from non-transfected THP-1 lysates treated with increasing concentrations of cmpd **2** and **6**.

Time and Concentration Screening – WB BRD2

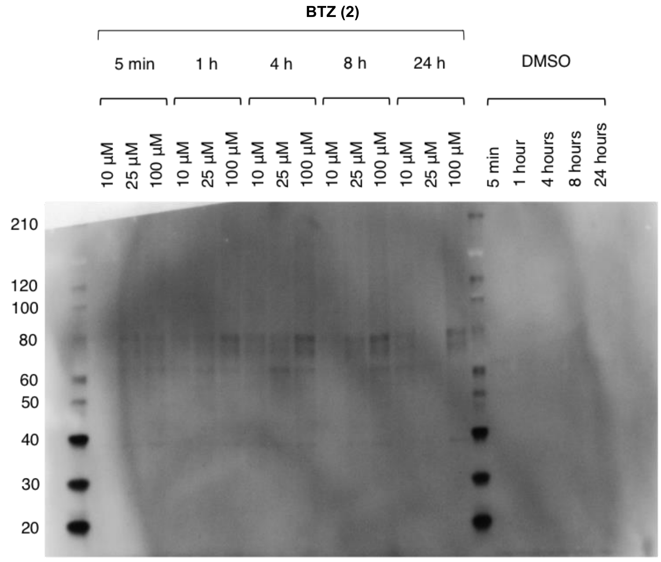

Supplemental Figure 8. WB developed on photographic film of BRD2 pull down from non-transfected THP-1 lysates treated with increasing concentrations of BTZ **2**

1.6 Quantitative Chemical Proteomics on THP-1 cell lysates

THP-1 lysate generation

THP-1 lysates were generated as described previously.[4] In brief, THP-1 cell pellets were thawed on ice and resuspended twice the cell pellet volume with ice cold lysis buffer (50 mM HEPES at pH 7.4), 150 mM NaCl, 1.5 mM $MgCl_2$, 1 mM DTT, 0.8% NP-40, 1X HALT protease inhibitor (Peirce Biotechnology). The resuspended cell pellet subjected to dounce homogenization and was spun at 800 x g for 10 min at 4 ^{0}C. The resulting supernatant (S0.8) was saved, while the pellet was further lysed using via pressure cycling, subjected to salt extraction, and treatment with benzonase. This further processed lysate was combined with the previously saved S0.8 lysate and used as input material for affinity enrichment experiments.

Affinity enrichment and compound competition experiments in THP-1 lysates.

For each affinity enrichment condition, 1 mL of THP-1 lysate (5 mg) was preincubated with either DMSO or 100 µM competition ligand for 16 hr at 4 ^{0}C in binding assay buffer (50 mM HEPES (pH 7.4), 150 mM NaCl, 1.5 mM MgCl2, 0.4% NP-40). The preincubated lysates, were then incubated with either DMSO or 100 µM BTZ for 4 hr at 4 ^{0}C. Probe-modified proteins were enriched, digested, subjected to isobaric labeling using TMT tags and sample analysis using nano-LC-MS/MS as previous described.[5]

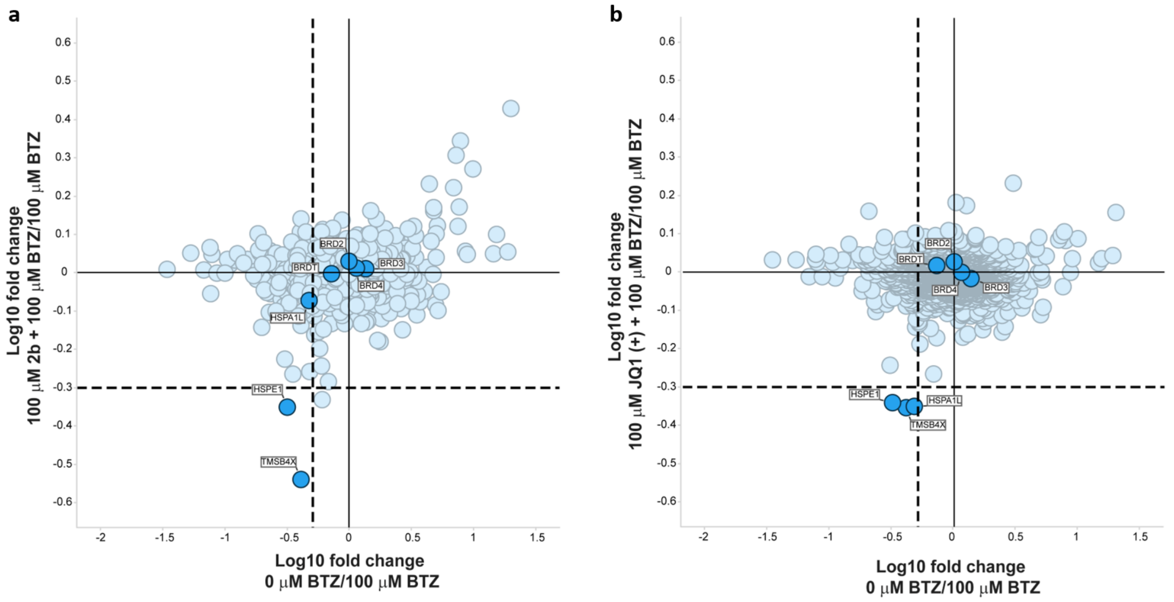

Supplemental Figure 9: Quantitative Proteomics Analysis of Competition Experiments using BTZ (**2**), its non-clickable analogue (**2b**), and a known BRD inhibitor (JQ1(+)). THP-1 cell lysates were incubated for 4 hr with 100 µM BTZ (**2**) at 4°C after preincubation with 100 µM non-clickable analogue (**2b**) or 100 µM JQ1(+), respectively. The control sample was preincubated with DMSO. BRD2, BRD3, BRD4 and BRDT were neither significantly enriched nor competed and could be detected only after performing targeted MS/MS experiments, indicating that the probe-labeled amount of BET family members was very low. Significant amounts of TMSB4X and HSPE1 were enriched and competed by pre-treatment with both **2b** and JQ1(+), thus indicating that these proteins could be potential BTZ (**2**) off-targets. Dashed lines indicate 50% competition (y-axis) or 2 fold enrichment (x-axis), plotted protein fold changes are median renormalized as described in supplementary **Table 1**.

Supplementary Table 1: Legend for BTZ chemoproteomics data.

**PROTEIN_AC**	Representative Uniprot protein accession number
**GENE_NAME**	Entrez gene name associated with Uniprot protein accession number
**DESCRIPTION**	Protein description
**PROTEIN_PROB**	ProteinProphet-derived protein probability
**SPECTRUM_COUNT**	Number of total redundant Mascot-derived peptide spectrum matches for this protein
**Q_PEP**	Number of unique peptide sequences for this protein that were used for protein quantitation (not shared with any other protein sequence in list of identified proteins)
**Q_SPEC**	Number of unique spectra matched to this protein that were used for quantitation (not shared with any other protein sequence in list of identified proteins)
**LOG10FC_JQ1**	Log10 fold change 100 uM JQ1(+) over 1 uM BTZ
**ADJ_PV_JQ1**	Adjusted P-value 100 uM JQ1(+) over 1 uM BTZ, proteins with Adj_Pvalue =1 were excluded from analysis
**LOG10FC_JQ1 MEDIAN normalized**	**as plotted in supplemental Fig 9:** Log10 fold change 100 uM JQ1(+) over 1 uM BTZ, , renormalized using the median log10 fold change for all proteins for (Log10 fold change 100uM JQ1(+) over 1 uM BTZ)
**LOG10FC_CMPD2B**	Log10 fold change 100uM Compound 2b over 1 uM BTZ
**ADJ_PV_CMPD2B**	Adjusted P-value 100uM Compound 2b over 1 uM BTZ, proteins with Adj_Pvalue =1 were excluded from analysis
**LOG10FC_CMPD2B MEDIAN normalized**	**as plotted in supplemental Fig 9:** Log10 fold change 100uM Compound 2b over 1 uM BTZ, renormalized using the median log10 fold change for all proteins for (Log10 fold change 100uM Compound 2b over 1 uM BTZ)
**LOG10FC_NOPROBE**	Log10 fold change 0 uM BTZ over 1 uM BTZ
**ADJ_PV_NOPROBE**	Adjusted P-value 0 uM BTZ over 1 uM BTZ, proteins with Adj_Pvalue =1 were excluded from analysis
**LOG10FC_NOPROBE MEDIAN normalized**	**as plotted in supplemental Fig 9:** Log10 fold change 0 uM BTZ over 1 uM BTZ, renormalized using the median log10 fold change for all proteins for (Log10 fold change 0 uM BTZ over 1 uM BTZ)

(4) Gower, C.; Thomas, J.; Harrington, E.; Murphy, J.; Chang, M.; Cornella-Taracido, I.; Jain, R.; Schirle, M.; Maly, D. Conversion of a Single Polypharmacological Agent into Selective Bivalent Inhibitors of Intracellular Kinase Activity *ACS Chem. Biol.* **2015**, *11*, 121-131.

(5) Thomas, J.; Brittain, S.; Lipps, J.; Llamas, L.; Jain, R.; Schirle, M. A Photoaffinity Labeling-Based Chemoproteomics Strategy for Unbiased Target Deconvolution of Small Molecule Drug Candidates. *Methods Mol Biol.* **2017**, *1647*, 1-18.

2. Crystallographic Data

2.1 Materials and Methods

Cloning, protein expression and purification of bromodomains

cDNA encoding reported human bromodomains were cloned, expressed and purified as previously described.[6]

Co-purification of BRD9 with bromotriazine (2)

Purified BRD9 (UniProt Q9H8M2, residues 14 – 134) was mixed with BTZ (**2**) in a molar ratio of 1:1.5 M and leave the mixture 3 h at room temperature. A further purification step was carried out using a size exclusion chromatography column in 20 mM Hepes pH 7.5, 350 mM NaCl. The co-eluted peak fraction of the complex was pooled, buffer exchanged in 20 mM Hepes pH 7.5, 100 mM and concentrated up to 15.2 mg/ml.

Crystallization

Aliquots of purified BRD9-BTZ (**2**) were set up for crystallization using a mosquito® crystallization robot (TTP Labtech, Royston UK). Coarse screens were typically setup onto Greiner 3-well plates using three different drop ratios of precipitant to protein per condition (200+100 nl, 150+150 nl and 100+200 nl). Initial hits were optimized further scaling up the drop sizes. All crystallizations were carried out using the sitting drop vapor diffusion method at 293.15 K. Crystals were grown by mixing 200 nl of the protein with 100 nl of reservoir solution containing 0.1M ammonium citrate pH7, 30% PEG3350. Diffraction quality crystals grew within a few days and were cryo-protected using the well solution supplemented with additional 20 % ethylene glycol and flash frozen in liquid nitrogen.

Data Collection and Structure solution

Crystallographic data was collected at Diamond beamline I03 at a wavelength of 0.9762 Å. Indexing and integration was carried out using XDS[7] and scaling was performed with AIMLESS[8]. Initial phases were calculated by molecular replacement with PHASER[9] using the apo BRD9 structure (PDB IDs 3HME). Initial models were built by ARP/wARP[10] followed by manual building in COOT[11]. Refinement was carried out in REFMAC5[12]. GRADE (global phasing)[13] was used to generate compound coordinates and cif files. Thermal motions from individual monomers of the octamer within the asymmetric unit were analyzed using TLSIN (REFMAC), and hydrogen atoms were included in late refinement cycles. All model validations were carried out using MolProbity[14]. Data collection and refinement statistics can be found in **Supplemental Table 1**. The model and structure factors have been deposited with PDB accession code: 5O4B.pdb

(6) Filippakopoulos, P.; Picaud, S.; Mangos, M.; Keates, T.; Lambert, J. P.; Barsyte---Lovejoy, D.; Felletar, I.; Volkmer, R.; Muller, S.; Pawson, T.; Gingras, A. C.; Arrowsmith, C. H.; Knapp, S., Histone recognition and large-scale structural analysis of the human bromodomain family. *Cell* **2012,** *149* (1), 214-231.

(7) Kabsch, W. (2010). XDS. *Acta Cryst. D66*, 125-132.

(8) P.R.Evans (2011). An introduction to data reduction: space-group determination, scaling and intensity statistics, Acta Cryst. D67, 282-292

(9) McCoy, A.J.; Grosse-Kunstleve, R.W.; Adams, P.D.; Winn, M.D.; Storoni, L.C.; Read., R.J. Phaser crystallographic software. *J Appl Crystallogr.*, **2007**, *40*, 658-674.

(10) Langer G., Cohen, SX., Lamzin, VS. and Perrakis, A. Automated macromolecular model building for X-ray crystallography using ARP/wARP version 7. *Nat. Protoc.*, **2008**, *3*(7), 1171-9.

(11) Emsley, P. and Cowtan, K. Coot: model-building tools for molecular graphics. *Acta Crystallogr. D Biol. Crystallogr.*, **2004**, *60*, 2126–2132.

(12) Murshudov, G. N.; Vagin, A. A.; Dodson, E. J. Refinement of macromolecular structures by the maximum-likelihood method. *Acta Crystallogr. Sect. D*, **1997**, *53*, 240–255.

(13) Smart, O. S.; Womack, T. O.; Sharff, A.; Flensburg, C.; Keller, P.; Paciorek, W.; Vonrhein, C.; Bricogne. G. (2011) Grade v1.102. Cambridge, United Kingdom, Global Phasing Ltd., http://www.globalphasing.com.

(14) Chen, V.B.; Arendall, W.B. 3rd; Headd, J.J.; Keedy, D.A.; Immormino, R.M.; Kapral, G.J.; Murray, L.W.; Richardson, J.S.; Richardson, D.C. MolProbity: all-atom structure validation for macromolecular crystallography. *Acta Cryst D*, **2010**, *66*, 12-21.

2.2 Crystallographic data collection and refinement statistics

Supplementary Table 2

**PROTEIN ID**	BRD9
**Ligand ID**	BTZ (**2**)
**Ligand Structure**	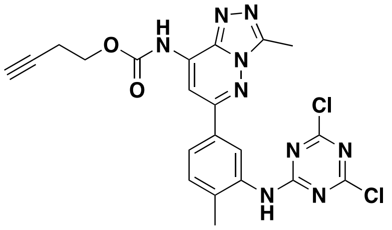
**Data collection**	
Space Group	$P\ 2\ 2_1\ 2_1$
Cell Dimensions	
a,b,c (Å)	60.02 138.40 140.08
α, β, γ (°)	90.00 90.00 90.00
Resolution[a] (Å)	29.55 (1.95)
Unique Observations	85016 (11992)
Completeness (%)	98.7 (96.5)
Redundancy	6.0 (6.0)
R_{sym}[b] or R_{merge}	0.057 (0.682)
$I/\sigma I$	18.2 (2.6)
Wavelength (Å)	0.9762
Phasing	MR
**Refinement**	
R_{work}[c] / R_{free}[d] (%)	18.83 / 22.63
Number of atoms	
protein / other / solvent	7617 / 48 / 285
B-Factors (Å^2)	
protein / other / solvent	39.17 / 53.89 / 39.94
R.M.S.D. Bond (Å)	0.009
R.M.S.D. Angle (°)	1.126
Ramachandran statistics	
Favored (%)	99.54
Outliers (%)	0
PDB ID	5O4B.pdb

[a] Highest resolution outer shell (in Å) shown in parentheses.
[b] $R_{sym} = \Sigma|I - \langle I \rangle|/\Sigma I$
[c] $R_{work} = \Sigma||F_{obs}| - |F_{calc}||/\Sigma|F_{obs}|$, where Fobs and Fcalc are the observed and calculated structure factors, respectively.
[d] R_{free} was calculated with 5% of the data excluded from the refinement calculation.
MR = Molecular Replacement

3. Docking Data

Docking experiments were performed using the Molsoft L.L.C.® ICM-Pro software and the crystal structure of BRD4(1) (PDB code: 4OGI).

3.1 BTZ (2) and BRD4(1)

The poses generated by docking BTZ (**2**) in the binding pocket of BRD4(1) and the corresponding scores are shown in **Supplementary Figure 9** and **Table 3**.

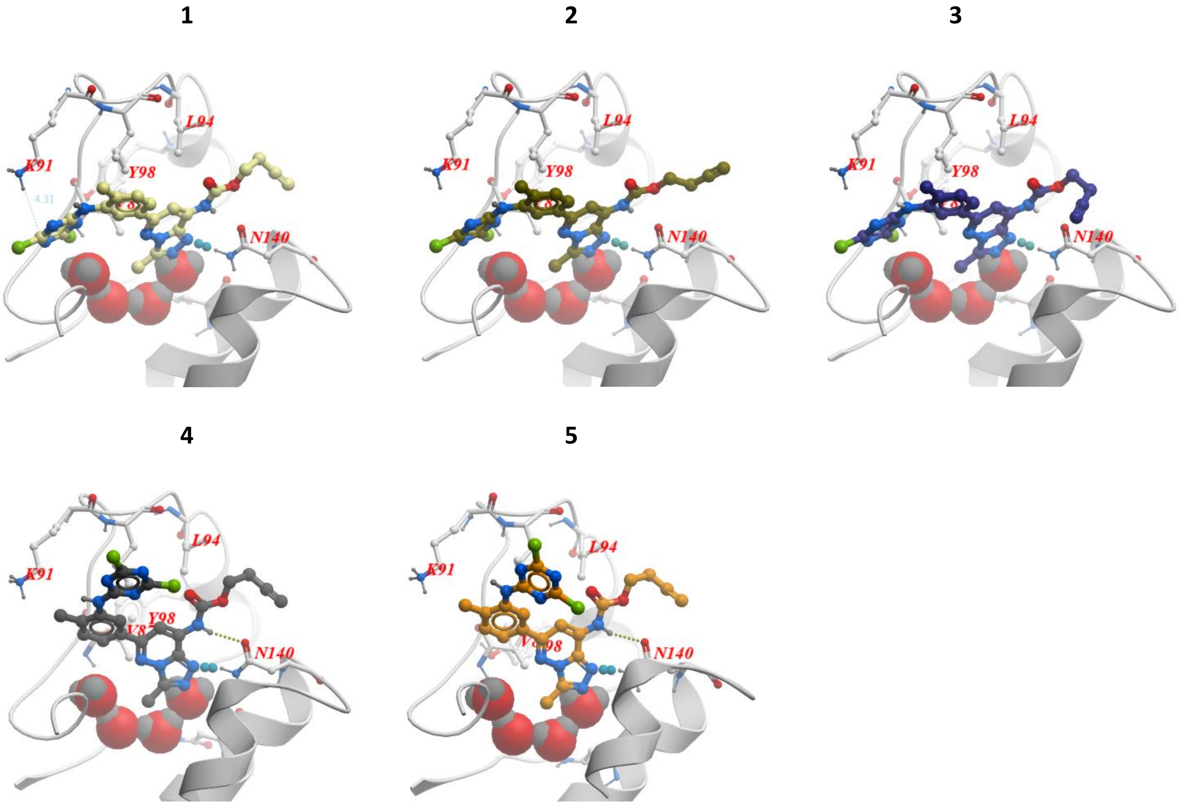

Supplementary Figure 10. Docking poses of BTZ (**2**) in the binding pocket of BRD4(1)

Supplementary Table 3

Conf Num	L	Score2	Score	Strain	RecConf	Steric	Torsion	Electro	Hbond	Hydroph	Surface
4	0	-31.376064	-35.859032	4.482969	BRD4_rec_rec1:1	-29.702127	3	5.99894	-7.193427	-7.504401	15.081486
5	0	-30.698519	-36.719933	6.021414	BRD4_rec_rec1:1	-30.039587	3	6.052549	-7.210052	-8.149517	14.968774
1	-1	-23.394709	-30.569562	7.174852	BRD4_rec_rec1:1	-33.304332	3	9.684781	-5.727765	-7.97593	19.170577
3	0	-23.008527	-29.960853	6.952326	BRD4_rec_rec1:1	-32.900119	3	9.775459	-5.723099	-7.744049	19.140557
2	0	-22.844849	-28.989453	6.144604	BRD4_rec_rec1:1	-32.044745	3	10.3145	-5.670201	-7.756173	18.653289

4. Synthetic Procedures and NMR Spectra

Solvents were used as supplied from Fisher Scientific®, Acros Organics® or Sigma Aldrich® without further purification. Where mixtures of solvents are specified, the stated ratios are volume:volume. Unless otherwise indicated, all aqueous solutions used were saturated. Reagents were used directly as supplied by major chemical suppliers. Column chromatography was carried out using a Biotage Isolera™ Prime and pre-packed columns for flash chromatography. Analytical thin-layer chromatography was carried out on Sigma-Aldrich® silica gel or aluminium oxide on TLA aluminium foils with fluorescent indicator at 254 nm. Visualization was carried out under ultra-violet irradiation (254 nm). NMR spectra were recorded on a Bruker AV400 (1H: 400 MHz, 13C: 101 MHz). Chemical shifts are quoted in ppm, based on appearance rather than interpretation, and are referenced to the residual non-deuterated solvent peak. High resolution mass spectra were recorded on a QTof interfaced with a LC or a Rapidfire™ mass spectrometer.
The previously reported synthesis of bromosporine[15] was optimised and adapted to produce compounds **2** and **2b**, as shown in **Scheme A** and **B**.

Synthetic Scheme A

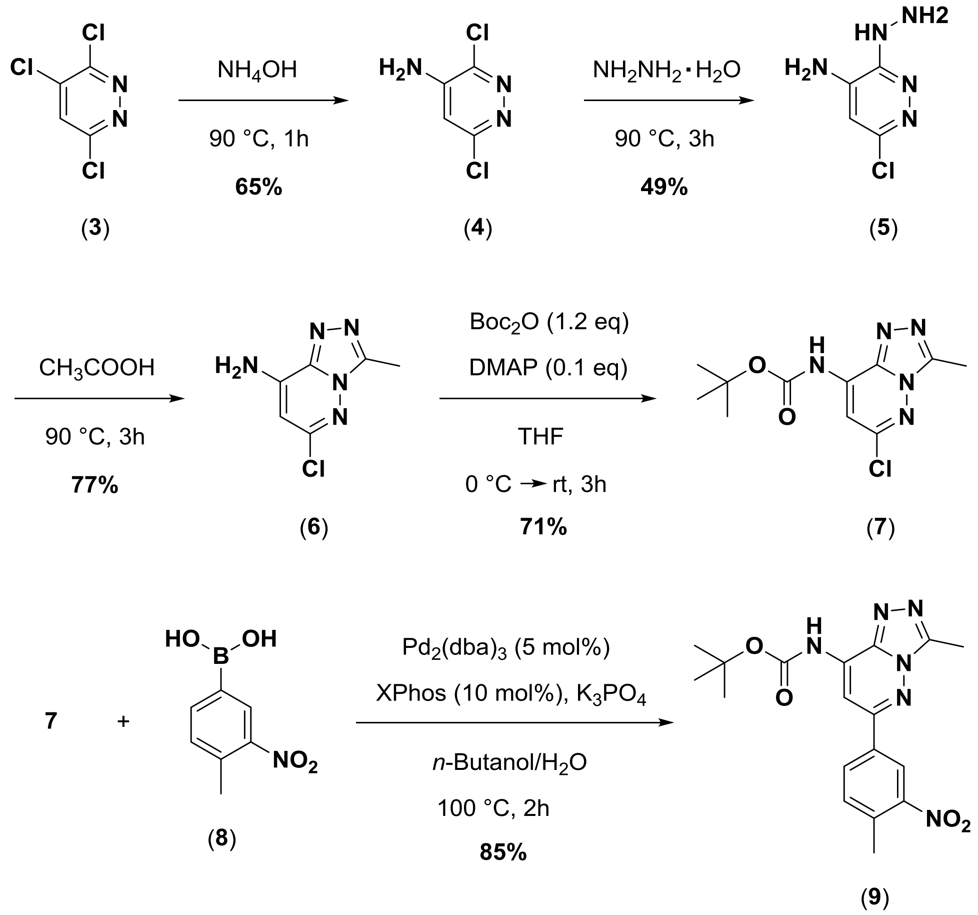

(15) Picaud, S.; Leonards, K.; Lambert, J.-P.; Dovey, O.; Wells, C.; Fedorov, O.; Monteiro, O.; Fujisawa, T.; Wang, C.-Y.; Lingard, H.; Tallant, C.; Nikbin, N.; Guetzoyan, L.; Ingham, R.; Ley, S. V.; Brennan, P.; Muller, S.; Samsonova, A.; Gingras, A.-C.; Schwaller, J.; Vassiliou, G.; Knapp, S. & Filippakopoulos, P. Promiscuous targeting of bromodomains by bromosporine identifies BET proteins as master regulators of primary transcription response in leukemia. *Sci. Adv.* **2016**, *2*, e1600760.

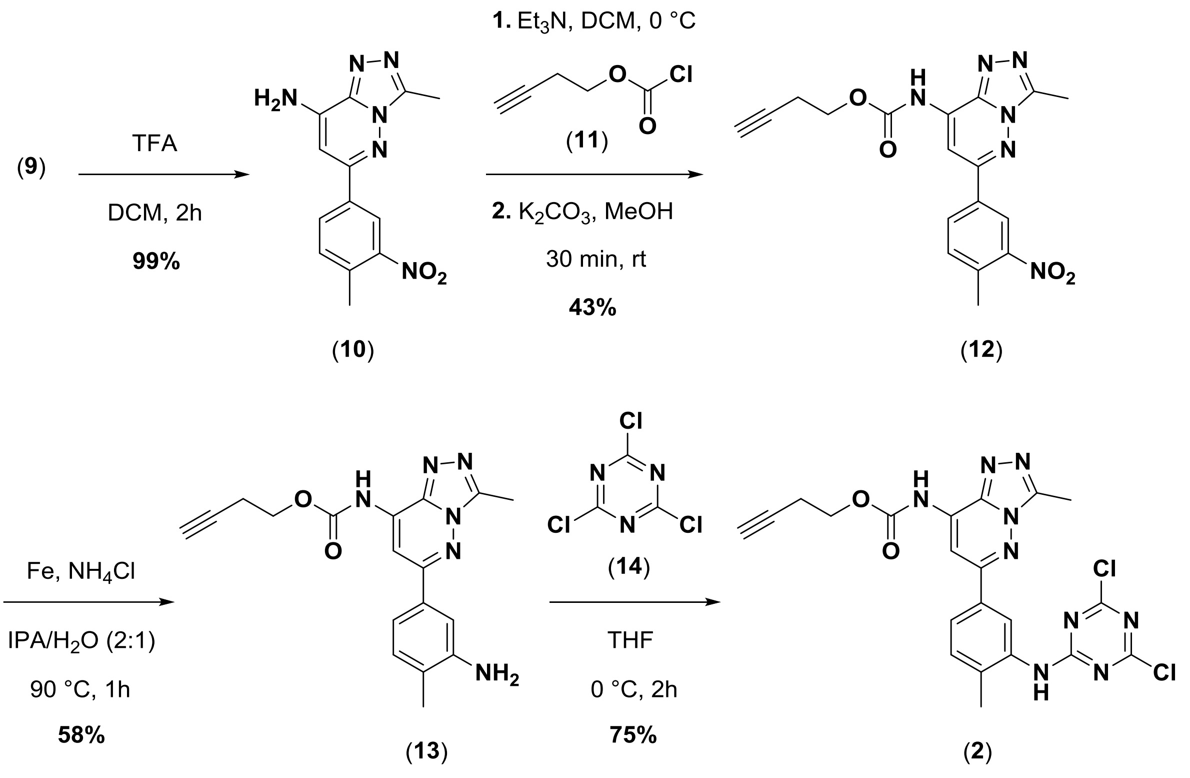

Synthetic Scheme B

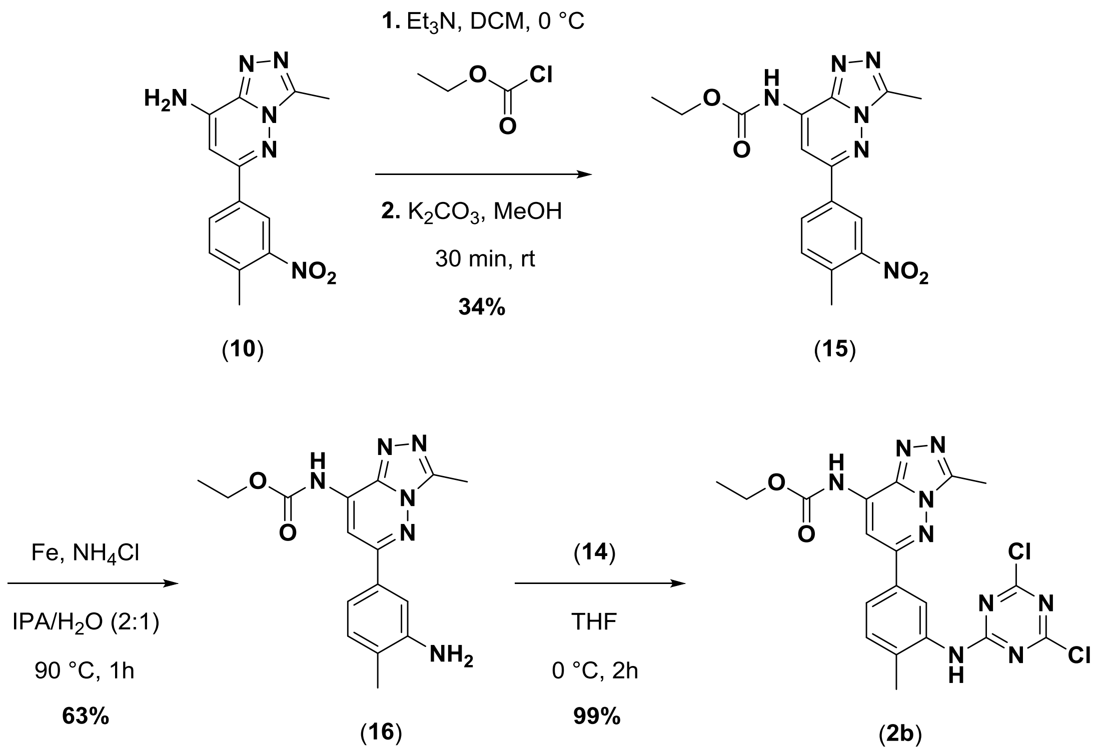

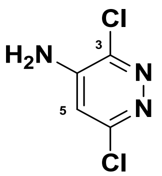

3,6-Dichloropyridazin-4-amine (4): In a sealed vial, 1.0 g (5.4 mmol) of 3,4,6-trichloropyridazine (**3**) were suspended in 10 mL of conc. ammonium hydroxide and heated up to 90 °C for 1 hour. The reaction was cooled down to room temperature, 10 mL of water were added and the precipitate was collected by filtration to give title compound as an off white solid (0.58 g, 65% yield).

^{1}H-NMR (400 MHz, DMSO-d$_6$) δ 6.83 (s, 1H, C(5)*H*), 7.16 (bs, 2H, N*H*$_2$);

^{13}C-NMR (100 MHz, DMSO-d$_6$) δ 108.5 (*C*(5)H), 143.7 (C(Ar)), 146.3 (C(Ar)), 154.5 (C(Ar)).

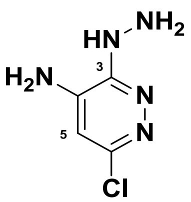

6-Chloro-3-hydrazinylpyridazin-4-amine (5): In a sealed vial, 1.0 g (6.1 mmol) of 3,6-dichloropyridazin-4-amine (**4**) were suspended in 10 mL of hydrazine monohydrate and heated up to 90 °C for 3 hours. The reaction was cooled down to room temperature, poured on ice, and the resulting precipitate was collected by filtration to give title compound as a pale yellow solid (0.48 g, 49% yield).

^{1}H-NMR (400 MHz, DMSO-d$_6$) δ 4.26 (bs, 2H, N*H*$_2$), 6.28 (bs, 2H, N*H*$_2$), 6.39 (s, 1H, C(5)*H*), 7.30 (bs, 1H, N*H*);

^{13}C-NMR (100 MHz, DMSO-d$_6$) δ 104.3 (*C*(5)H), 137.9 (C(Ar)), 147.1 (C(Ar)), 151.1 (C(Ar)).

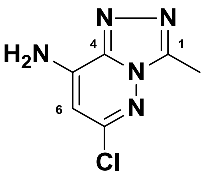

6-Chloro-3-methyl-[1,2,4]triazolo[4,3-*b*]pyridazin-8-amine (6): In a round bottom flask, 3.0 g (19.0 mmol) of 6-chloro-3-hydrazinylpyridazin-4-amine (**5**) were suspended in 25 mL of conc. acetic acid and heated up to 90 °C for 3 hours. The reaction was cooled down to room temperature, poured on ice, and the resulting precipitate was collected by filtration. A solution of sat. sodium carbonate was added to the obtained solid and the resulting suspension stirred for half an hour at room temperature.
The insoluble solid was filtered off to give title compound as an off white solid (2.7 g, 77% yield).

^{1}H-NMR (400 MHz, DMSO-d$_6$) δ 2.59 (s, 3H, C*H*$_3$), 6.12 (s, 1H, C(6)*H*), 7.90 (bs, 2H, N*H*$_2$);

^{13}C-NMR (100 MHz, DMSO-d$_6$) δ 9.9 (*C*H$_3$), 94.0 (*C*(6)H), 139.8 (C(Ar)), 144.3 (C(Ar)), 147.2 (C(Ar)) 149.8 (C(Ar)).

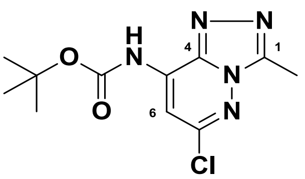

***Tert*-butyl (6-chloro-3-methyl-[1,2,4]triazolo[4,3-*b*]pyridazin-8-yl)carbamate (7):** In a round bottom flask, di-*tert*-butyl dicarbonate (2.85 g, 13.1 mmol) and DMAP (0.13 g, 1.1 mmol) were added to a suspension of 6-chloro-3-methyl-[1,2,4]triazolo[4,3-*b*]pyridazin-8-amine (**6**) (2.0 g, 10.9 mmol) in 40 mL of THF at 0 °C. The reaction was allowed to warm up to room temperature over 3 hours. The solvent was evaporated, the crude oil absorbed on silica and purified by flash chromatography on silica gel (KP-Sil 25 g; 20:80 EtOAc : Cyclohexane) to give title compound as a white solid (2.2 g, 71% yield).

^{1}H-NMR (400 MHz, DMSO-d$_6$) δ 1.51 (s, 9H, 3 x C*H*$_3$), 2.65 (s, 3H, C*H*$_3$), 7.60 (s, 1H, C(6)*H*), 10.83 (bs, 1H, N*H*);

^{13}C-NMR (100 MHz, DMSO-d$_6$) δ 9.9 (*C*H$_3$), 28.2 (3 x *C*H$_3$), 82.4 (*C*(CH$_3$)$_3$), 103.0 (*C*(6)H), 136.7 (C(Ar)), 138.9 (C(Ar)), 147.7 (C(Ar)) 150.0 (C(Ar)), 152.7 (C=O).

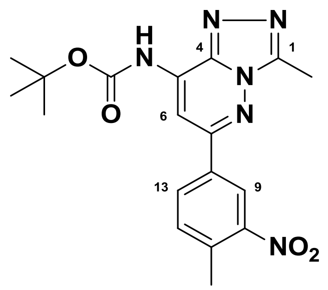

***Tert*-butyl (3-methyl-6-(4-methyl-3-nitrophenyl)-[1,2,4]triazolo[4,3-*b*]pyridazin-8-yl) carbamate (9):** 4-methyl-3-nitrophenylboronic acid (**8**) (1.4 g, 7.0 mmol), $Pd_2(dba)_3$ (0.3 g, 5 mol%), XPhos (0.7 g, 10 mol%) and a solution of potassium phosphate (3.0 g, 14.1 mmol) in 6 mL of water were added to a stirring solution of tert-butyl (6-chloro-3-methyl-[1,2,4]triazolo[4,3-*b*]pyridazin-8-yl)carbamate (2.0 g, 7.0 mmol) in 60 mL of degassed *n*-butanol. The reaction was heated up to 100 °C for 2h. The solvent was evaporated; the reaction was dissolved in EtOAc and filtered through Celite. The crude dark red oil was purified by flash column chromatography on silica gel (KP-Sil 50 g; 0 to 5% MeOH : dichloromethane). The obtained yellow gum was triturated with diethyl ether and filtered to give title compound as yellow solid (2.3 g, 85% yield).

^{1}H-NMR (400 MHz, $CDCl_3$) δ 1.61 (s, 9H, 3 x C*H*$_3$), 2.71 (s, 3H, C*H*$_3$), 2.90 (s, 3H, C*H*$_3$), 7.53 (d, 1H, J_o = 8.0 Hz C(12)*H*), 8.15 (dd, 1H, J_o = 8.0 Hz, J_m = 2.0 Hz, C(13)*H*), 8.22 (s, 1H, C(6)*H*), 8.51 (bs, 1H, N*H*), 8.65 (d, 1H, J_m = 2.0 Hz C(9)*H*);

^{13}C-NMR (100 MHz, $CDCl_3$) δ 5.2 (*C*H$_3$), 15.6 (*C*H$_3$), 23.3 (3 x *C*H$_3$), 78.7 (*C*(CH$_3$)$_3$), 95.3 (*C*(6)H), 118.7 (*C*H(Ar)), 126.5 (*C*H(Ar)), 128.7 (*C*H(Ar)) 129.7 (*C*(Ar)), 129.9 (*C*(Ar)), 131.1 (*C*(Ar)), 134.0 (*C*(Ar)), 143.8 (*C*(Ar)), 145.0 (*C*(Ar)), 146.9 (*C*(Ar)), 148.7 (*C*=O).

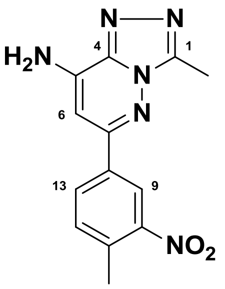

3-methyl-6-(4-methyl-3-nitrophenyl)-[1,2,4]triazolo[4,3-b]pyridazin-8-amine (10): trifluoroacetic acid (8.0 mL) was added to a solution of *tert*-butyl(3-methyl-6-(4-methyl-3-nitrophenyl)-[1,2,4]triazolo[4,3-*b*]pyridazin-8-yl) carbamate (**9**) in dichloromethane (100 mL). The reaction was stirred at room temperature for 2h. The solvent was removed in vacuum and the obtained solid suspended in a saturated solution of sodium carbonate and stirred for 30 minutes. The resulting suspension was filtered to give title compound as light yellow solid (0.74 g, 99% yield).

^{1}H-NMR (400 MHz, DMSO-d$_6$) δ 2.60 (s, 3H, C*H*$_3$), 2.70 (s, 3H, C*H*$_3$), 6.64 (s, 1H, C(6)*H*), 7.62 (bs, 2H, N*H*$_2$), 7.68 (d, 1H, J_o = 8.1 Hz C(12)*H*), 8.19 (dd, 1H, J_o = 8.0 Hz, J_m = 2.0 Hz, C(13)*H*), 8.49 (d, 1H, J_m = 1.9 Hz C(9)*H*);

^{13}C-NMR (100 MHz, DMSO-d$_6$) δ 10.1 (*C*H$_3$), 19.9 (*C*H$_3$), 92.0 (*C*(6)H), 122.8 (*C*(9)H), 131.6 (*C*(13)H), 134.0 (*C*(12)H) 134.8 (*C*(Ar)), 135.6 (*C*(Ar)), 140.3 (*C*(Ar)), 143.7 (*C*(Ar)), 147.5 (*C*(Ar)), 149.7 (*C*(Ar)), 152.4 (*C*(Ar)).

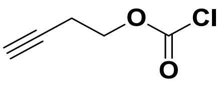

But-3-yn-1-yl carbonochloridate (11): Triphosgene (10.6 g, 35.7 mmol) was dissolved in 20 mL of anhydrous THF and the solution cooled down to 0°C. But-3-yn-1-ol (5.4 mL, 71.3 mmol) was diluted in 10 mL of dry THF and added dropwise over 2h at 0°C. The resultant mixture was warmed up to room temperature and stirred overnight. Anhydrous pyridine (8.6 mL, 107 mmol) was added dropwise to the solution over 1h at 0°C and then the reaction was warmed to room temperature and stirred for a further hour. Precipitated pyridinium hydrochloride salt was removed by filtration and the THF evaporated under vacuum to give title compound as an orange oil (5.8 g, quantitative yield).

^{1}H-NMR (400 MHz, $CDCl_3$) δ 2.09 (t, 1H, J = 2.7 Hz, ≡C*H*), 2.67 (td, 2H, J = 6.8 Hz, J = 2.6 Hz, C*H*$_2$), 4.43 (t, 2H, J = 6.8 Hz, C*H*$_3$).

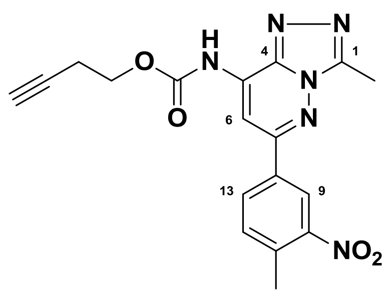

But-3-yn-1-yl (6-(3-nitro-4-methylphenyl)-3-methyl-[1,2,4]triazolo[4,3-*b*]pyridazin-8-yl)carbamate (12): Triethylamine (0.3 mL, 2.1 mmol) was added to a suspension of 3-methyl-6-(4-methyl-3-nitrophenyl)-[1,2,4]triazolo[4,3-*b*]pyridazin-8-amine (0.3 g, 1.05 mmol) in dry dichloromethane (10 mL) at 0 °C. After 30 min but-3-yn-1-yl chloroformate (0.24 mL, 2.1 mmol) was added over 2 hours and the reaction mixture warmed up to room temperature and stirred overnight. This step was repeated until complete consumption of starting material. The reaction was absorbed on silica and purified by flash column chromatography (KP-Sil 25 g, 0 to 5% DCM/MeOH) to give the double carbamate as a brown oil (0.46 g, 0.96 mmol). The double carbamate was redissolved in 10 mL of methanol, potassium carbonate (0.29 g, 2.1 mmol) was added and the reaction stirred for 30 minutes at room temperature. Upon completion, the excess of potassium carbonate was filtered off, the filtrate was absorbed on silica and purified by flash column chromatography (KP-Sil 25 g; 0 to 2% MeOH : dichloromethane) to give title compound as light yellow solid (0.17 g, 43% yield).

^{1}H-NMR (400 MHz, DMSO-d$_6$) δ 2.62 (s, 3H, C*H*$_3$), 2.64 (td, 2H, J_1 = 6.5 Hz, J_2 = 2.5 Hz, C*H*$_2$), 2.77 (s, 3H, C*H*$_3$), 2.94 (t, 1H, J_2 = 2.7 Hz, ≡C*H*), 4.31 (t, 2H, J_1 = 6.6 Hz, C*H*$_2$), 7.73 (d, 1H, J_o = 7.7 Hz, C(12)*H*), 8.15 (s, 1H, C(6)*H*), 8.23 (dd, 1H, J_o = 8.0 Hz, J_m = 2.0 Hz C(13)*H*), 8.54 (d, 1H, J_m = 2.0 Hz, C(9)*H*), 11.15 (bs, 1H, N*H*);

^{13}C-NMR (100 MHz, DMSO-d$_6$) δ 10.0 (*C*H$_3$), 19.0 (*C*H$_2$), 19.9 (C*H*$_3$), 64.0 (C*H*$_2$), 73.3 (≡*C*H), 81.1 (*C*≡CH), 101.3 (*C*(6)H), 123.1 (*C*(9)H), 131.8 (*C*(13)H), 134.3 (*C*(12)H), 134.6 (*C*(Ar)), 135.6 (*C*(Ar)), 135.7 (*C*(Ar)), 139.3 (*C*(Ar)), 148.1 (*C*(Ar)), 149.8 (*C*(Ar)), 152.5 (*C*(Ar)), 154.0 (*C*=O).

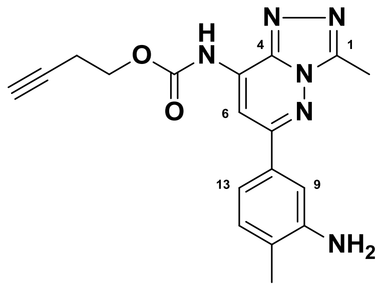

But-3-yn-1-yl (6-(3-amino-4-methylphenyl)-3-methyl-[1,2,4]triazolo[4,3-*b*]pyridazin-8-yl)carbamate (13): In a sealed vial, iron powder (92 mg, 1.6 mmol) and ammonium chloride (354 mg, 6.6 mmol) were added to a solution of butyn-4-yl (3-methyl-6-(4-nitrophenyl)-[1,2,4]triazolo[4,3-*b*]pyridazin-8-yl)carbamate (**15**) (210 mg, 0.5 mmol) in a mixture of IPA (6 mL) and water (3 mL). The reaction was stirred at 90 °C for 1 hour, cooled down to room temperature and evaporated. The crude material was dissolved in methanol and filtered through Celite®. The filtrate was absorbed on silica and purified by flash column chromatography (KP-Ultra 10 g; 0 to 5% MeOH : dichloromethane) to give title compound as light yellow solid (112 mg, 58% yield).

^{1}H-NMR (400 MHz, DMSO-d$_6$) δ 2.13 (s, 3H, C*H*$_3$), 2.62 (td, 2H, J_1 = 6.6, J_2 = 2.7 Hz, C*H*$_2$), 2.73 (s, 3H, C*H*$_3$), 2.94 (t, 1H, J_2 = 2.7 Hz, ≡C*H*), 4.29 (t, 2H, J_1 = 6.6 Hz, C*H*$_2$), 5.21 (bs, 2H, N*H*$_2$), 7.07-7.15 (m, 2H, 2 x C(Ar)*H*), 7.27 (d, 1H, J_m = 1.6 Hz C(9)*H*), 8.07 (s, 1H, C(6)*H*), 10.94 (bs, 1H, N*H*)

^{13}C-NMR (100 MHz, DMSO-d$_6$) δ 10.0 (*C*H$_3$), 17.9 (*C*H$_3$), 19.0 (C*H*$_2$), 63.9 (C*H*$_2$), 73.3 (≡*C*H), 81.1 (*C*≡CH), 101.8 (*C*(6)H), 112.3 (*C*(9)H), 115.2 (*C*(13)H), 124.4 (*C*(Ar)), 131.0 (*C*(12)H), 133.7 (*C*(Ar)), 134.9 (*C*(Ar)), 139.4 (*C*(Ar)), 147.7 (*C*(Ar)), 147.8 (*C*(Ar)), 154.0 (*C*(Ar)), 155.2 (*C*=O).

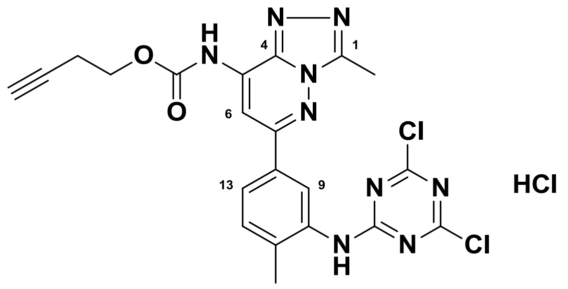

But-3-yn-1-yl (6-(3-((4,6-dichloro-1,3,5-triazin-2-yl) amino)-4-methylphenyl)-3-methyl-[1,2,4] triazolo [4,3-*b*]pyridazin-8-yl) carbamate hydrochloride (BTZ-2): A stirred solution of cyanuric chloride **14** (42.3 mg, 0.23 mmol) in anhydrous tetrahydrofuran (7.5 mL) was cooled down to 0 °C. A suspension of but-3-yn-1-yl (6-(3-amino-4-methylphenyl)-3-methyl-[1,2,4]triazolo[4,3-*b*]pyridazin-8-yl)carbamate (**13**) (67 mg, 0.19 mmol) in 1.8 mL of anhydrous THF was added drop wise (1.2 mL/hour) and the resulting mixture stirred at 0 °C for 2 h. The solvent was evaporated to give title compound as orange solid (77.3 mg, 75% yield).

^{1}H-NMR (400 MHz, DMSO-d$_6$) δ 2.30 (s, 3H, C*H*$_3$), 2.63 (td, 2H, J_1 = 6.6 Hz, J$_2$ = 2.7 Hz, C*H*$_2$), 2.76 (s, 3H, C*H*$_3$), 2.94 (t, 1H, J_2 = 2.7 Hz, ≡C*H*), 4.30 (t, 2H, J_1 = 6.6 Hz, C*H*$_2$), 7.53 (d, 1H, J_o = 8.1 Hz, C(12)*H*), 7.86 (dd, 1H, J_o = 8.0 Hz, J_m = 2.0 Hz C(13)*H*), 7.99 (d, 1H, J_m = 1.9 Hz, C(9)*H*), 8.13 (s, 1H, C(6)*H*), 10.87 (bs, 1H, N*H*), 11.08 (bs, 1H, N*H*), 11.16 (bs, 1H, =N*H*);

^{13}C-NMR (101 MHz, DMSO-d$_6$) δ 9.9 (*C*H$_3$), 18.3 (*C*H$_3$), 19.0 (C*H*$_2$), 64.2 (C*H*$_2$), 73.3 (≡*C*H), 81.1 (*C*≡CH), 103.3 (*C*(6)H), 125.4 (*C*(9)H), 126.1 (*C*(13)H), 132.0 (*C*(12)H), 133.4 (C(Ar)), 135.1 (C(Ar)), 135.9 (*C*(Ar)), 137.2 (*C*(Ar)), 138.8 (*C*(Ar)), 148.0 (*C*(Ar)), 150.4 (*C*(Ar)), 153.8 (*C*=O), 165.6 (*C*(Ar)), 169.5 (*C*(Ar)), 170.2 (*C*(Ar)).*

HRMS Calculated for $C_{21}H_{17}Cl_2N_9O_2$ [M+1]$^+$ = 498.0961 (^{35}Cl), 500.0931 (^{37}Cl); Observed [M+1]$^+$ = 498.1024 (^{35}Cl), 500.1009 (^{37}Cl).

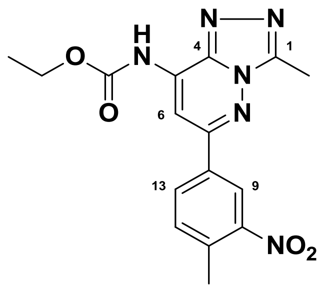

Ethyl (6-(3-nitro-4-methylphenyl)-3-methyl-[1,2,4]triazolo[4,3-*b*]pyridazin-8-yl)carbamate (15): Triethylamine (0.3 mL, 2.1 mmol) was added to a suspension of 3-methyl-6-(4-methyl-3-nitrophenyl)-[1,2,4]triazolo[4,3-*b*]pyridazin-8-amine (**10**) (300 mg, 1.05 mmol) in dry dichloromethane (10 mL) at 0 °C. After 30 min ethyl chloroformate (0.16 mL, 2.1 mmol) was added over 2 hours and the reaction mixture warmed to room temperature. This step was repeated until complete consumption of starting material. The reaction was quenched with 25 mL of water, filtered through a phase separator, absorbed on silica and purified by flash column chromatography (KP-Sil 10 g, 0 to 5% DCM/MeOH) to give a yellow solid. The crude mixture was redissolved in 6 mL of methanol, potassium carbonate (292 mg, xx mmol) was added and the reaction stirred for 30 minutes at room temperature. Upon completion, the excess of potassium carbonate was filtered off; the filtrate was absorbed on silica and purified twice by flash column chromatography (KP-Ultra 10 g; 0 to 2% MeOH : dichloromethane) to give title compound as orange solid (130 mg, 34% yield).

^{1}H-NMR (400 MHz, CDCl$_3$) δ 1.41 (t, 3H, *J* = 7.1 Hz, C*H*$_3$), 2.70 (s, 3H, C*H*$_3$), 2.89 (s, 3H, C*H*$_3$), 4.38 (q, 2H, *J* = 7.1 Hz, C*H*$_2$), 7.53 (d, 1H, J_o = 8.0 Hz, C(12)*H*), 8.13 (dd, 1H, J_o = 8.0 Hz, J_m = 2.0 Hz C(13)*H*), 8.25 (s, 1H, C(6)*H*), 8.64 (d, 1H, *J* = 1.9 Hz, C(9)*H*), 8.83 (bs, 1H, N*H*);

^{13}C-NMR (101 MHz, CDCl$_3$) δ 9.95 (*C*H$_3$), 14.3 (*C*H$_3$), 20.4 (*C*H$_3$), 62.9 (C*H*$_2$), 100.3 (*C*(6)H), 123.4 (*C*(9)H), 131.2 (*C*(13)H), 133.5 (*C*(12)H), 134.3 (*C*(Ar)), 134.5 134.3 (*C*(Ar)), 135.9 (*C*(Ar)), 138.8 (*C*(Ar)), 148.6 (*C*(Ar)), 149.8 (*C*(Ar)), 152.9 (*C*(Ar)), 153.3 (*C*=O).

* Due to the presence of rotamers and the weakness of the resulting carbon signals, the current spectrum is reported as a combination of ^{13}C and APT spectra.

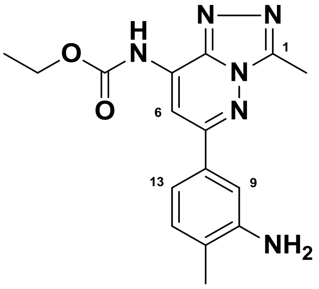

Ethyl (6-(3-amino-4-methylphenyl)-3-methyl-[1,2,4]triazolo[4,3-*b*]pyridazin-8-yl)carbamate (16): In a sealed vial, iron powder (60 mg, 0.05 mmol) and ammonium chloride (225 mg, 4.2 mmol) were added to a solution of ethyl (3-methyl-6-(4-nitrophenyl)-[1,2,4]triazolo[4,3-*b*]pyridazin-8-yl)carbamate (**15**) (125 mg, 0.35 mmol) in a mixture of IPA (3 mL) and water (1.5 mL). The reaction was stirred at 90 °C for 2 hours, cooled down to room temperature and evaporated. The crude material was dissolved in methanol and filtered through Celite®. The filtrate was absorbed on silica and purified by flash column chromatography (KP-Ultra 10 g; 0 to 2% MeOH : dichloromethane) to give title compound as yellow solid (72 mg, 63% yield).

^{1}H-NMR (400 MHz, DMSO-d$_6$) δ 1.30 (t, 3H, J = 7.1 Hz, C*H*$_3$), 2.13 (s, 3H, C*H*$_3$), 2.73 (s, 3H, C*H*$_3$), 4.26 (q, 2H, J = 7.1 Hz, C*H*$_2$), 5.18 (bs, 2H, N*H*$_2$), 7.08 (dd, 1H, J_o = 7.07 Hz, J_m = 1.6 Hz, C(12)*H*), 7.11 (d, 1H, J_o = 7.11 Hz, C(11)*H*), 7.27 (d, 1H, J_m = 1.3 Hz, C(8)*H*), 8.09 (s, 1H, C(5)*H*), 10.78 (bs, 1H, N*H*);

^{13}C-NMR (100 MHz, DMSO-d$_6$) δ 10.0 (*C*H$_3$), 14.8 (*C*H$_3$), 17.9 (*C*H$_3$), 62.1 (*C*H$_2$), 101.4 (*C*(6)H), 112.3 (*C*(9)H), 115.2 (*C*(13)H), 124.4 (*C*(Ar)), 131.0 (*C*(12)H), 133.7 (*C*(Ar)), 135.1 (*C*(Ar)), 139.4 (*C*(Ar)), 147.7 (*C*(Ar)), 147.8 (*C*(Ar)), 154.2 (*C*(Ar)), 155.2 (*C*=O).

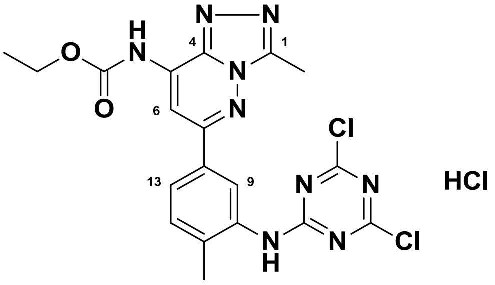

Ethyl (6-(3-((4,6-dichloro-1,3,5-triazin-2-yl)amino)-4-methylphenyl)-3-methyl-[1,2,4]triazolo[4,3-*b*]pyridazin-8-yl)carbamate hydrochloride (2b): A stirred solution of cyanuric chloride (**14**) (45 mg, 0.24 mmol) in anhydrous tetrahydrofuran (3 mL) was cooled down to 0 °C. A suspension of ethyl (6-(3-amino-4-methylphenyl)-3-methyl-[1,2,4]triazolo[4,3-*b*]pyridazin-8-yl)carbamate (**16**) (67 mg, 0.19 mmol) in 1.2 mL of anhydrous THF was added drop wise (1.2 mL/hour) and the resulting mixture stirred at 0 °C for 2 h. The solvent was evaporated to give title compound as orange solid (106 mg, 99% yield).

^{1}H-NMR (400 MHz, DMSO-d$_6$) δ 1.30 (t, 3 H, J = 7.1 Hz, CH$_3$), 2.30 (s, 3H, C*H*$_3$), 2.76 (s, 3H, C*H*$_3$), 4.27 (q, 2H, J = 7.1 Hz, C*H*$_2$), 7.53 (d, 1H, J_o = 8.1 Hz, C(12)*H*), 7.85 (dd, 1H, J_o = 8.0 Hz, J_m = 1.8 Hz C(13)*H*), 7.98 (d, 1H, J_m = 1.5 Hz, C(12)*H*), 8.17 (s, 1H, C(6)*H*), 10.88 (bs, 1H, N*H*), 10.96 (bs, 1H, N*H*), 11.17 (bs, 0.5H, N*H*);

^{13}C-NMR (100 MHz, DMSO-d$_6$) δ 9.96 (*C*H$_3$), 14.74 (*C*H$_3$), 18.23 (*CH*$_3$), 62.25 (C*H*$_2$), 101.94 (*C*(6)H), 125.60 (*C*(9)H), 126.13 (*C*(13)H), 132.01 (*C*(12)H), 133.65 (*C*(Ar)), 135.40 (*C*(Ar)), 135.89 (*C*(Ar)), 137.37 (*C*(Ar)), 148.02 (*C*(Ar)), 150.37 (*C*(Ar)), 154.08 (*C*=O), 154.32 (*C*(Ar)), 165.58 (*C*(Ar)), 169.55 (*C*(Ar)), 170.20 (*C*(Ar));

HRMS Calculated for $C_{19}H_{17}Cl_2N_9O_2$ [M+1]$^+$ = 474.0961 (^{35}Cl), 476.0931 (^{37}Cl); Observed [M+1]$^+$ = 474.1029 (^{35}Cl), 476.0997 (^{37}Cl).

NMR Spectra

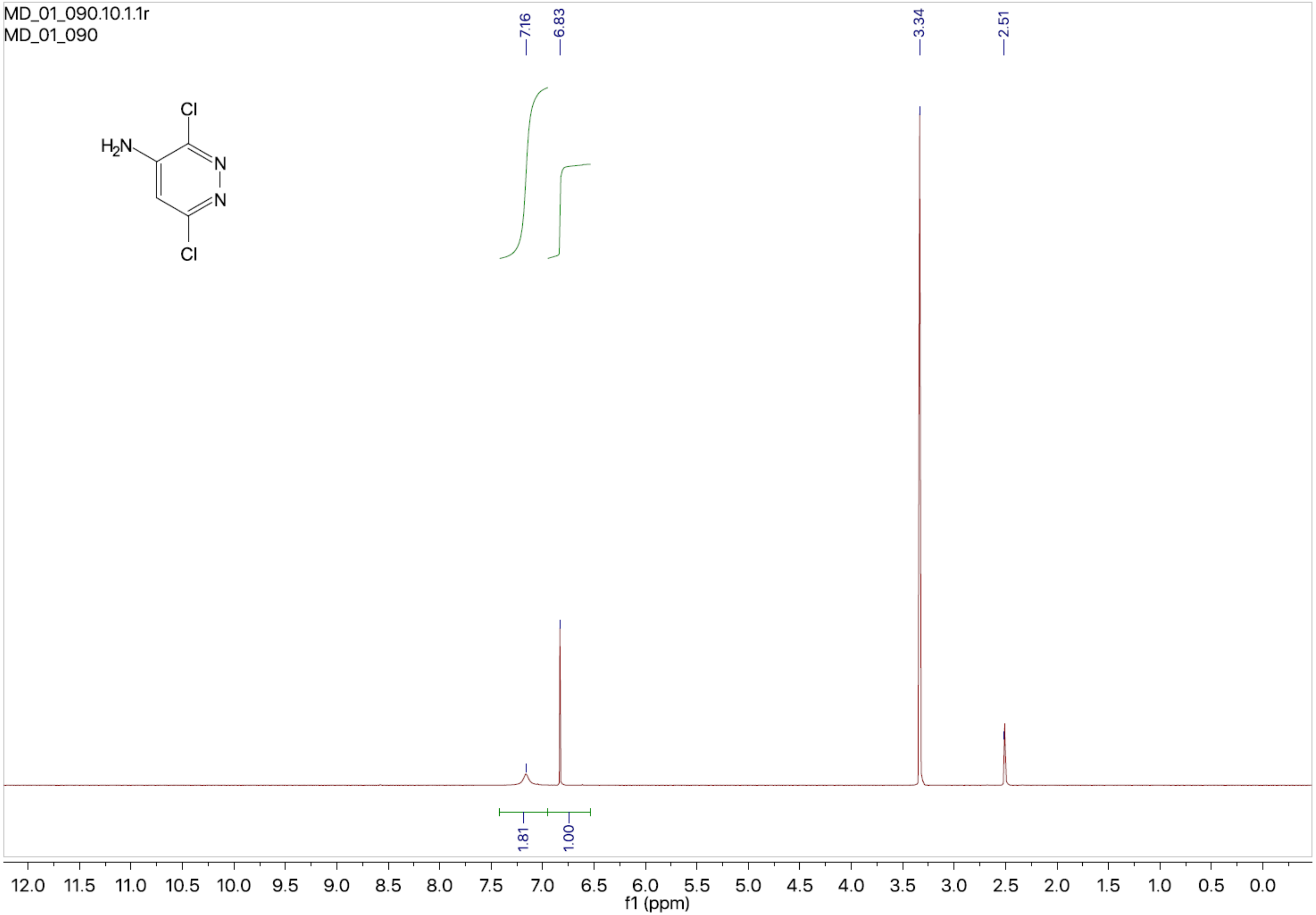
MD_01_090.10.1.1r
MD_01_090
Cl
H_2N
N
N
Cl
7.16
6.83
3.34
2.51
1.81
1.00
12.0 11.5 11.0 10.5 10.0 9.5 9.0 8.5 8.0 7.5 7.0 6.5 6.0 5.5 5.0 4.5 4.0 3.5 3.0 2.5 2.0 1.5 1.0 0.5 0.0
f1 (ppm)

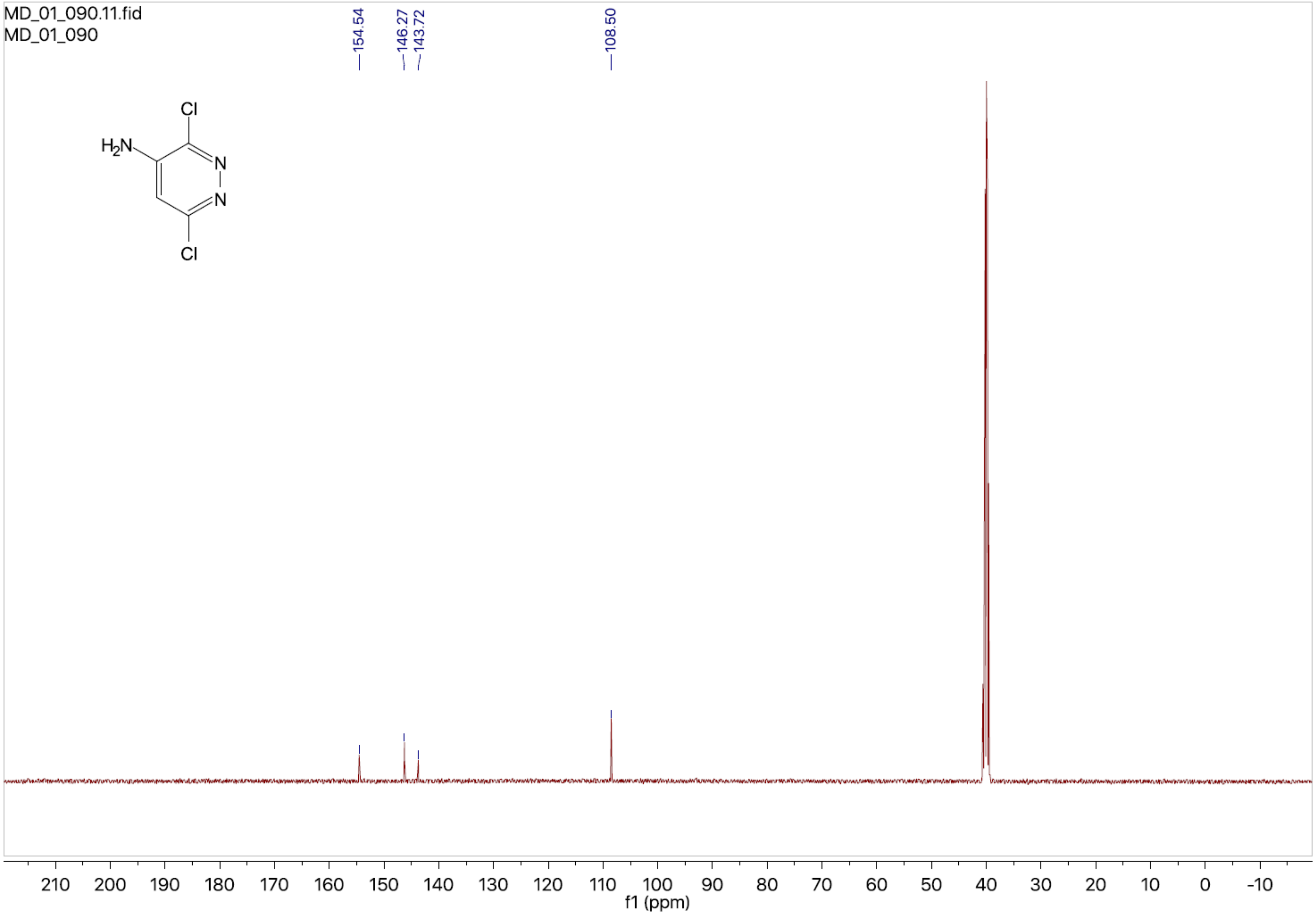

MD_01_090.11.fid
MD_01_090
154.54
146.27
143.72
108.50
Cl
H2N
N
N
Cl
210
200
190
180
170
160
150
140
130
120
110
100
90
80
70
60
50
40
30
20
10
0
-10
f1 (ppm)

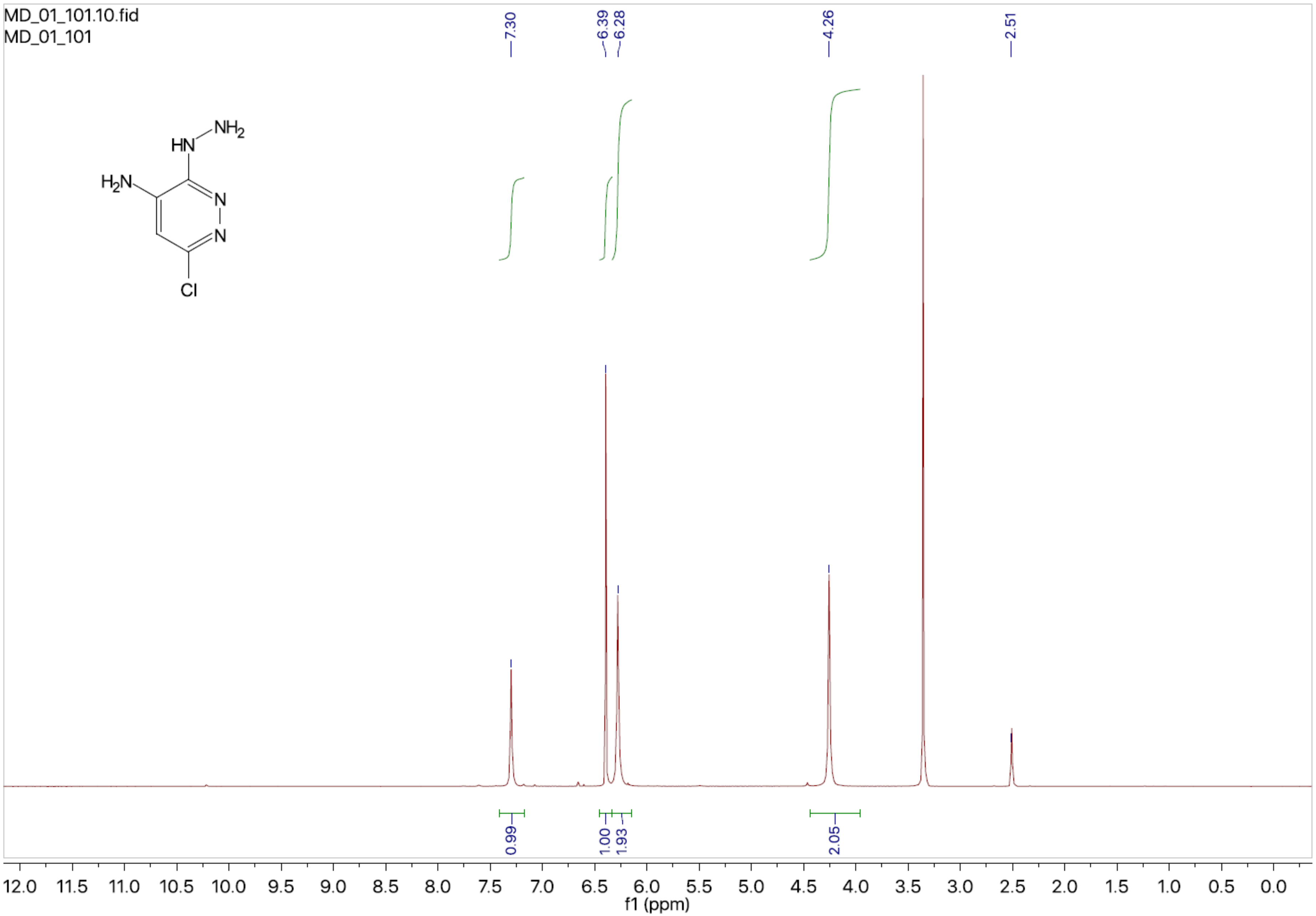
MD_01_101.10.fid
MD_01_101
7.30
6.39
6.28
4.26
2.51
0.99
1.00
1.93
2.05
12.0 11.5 11.0 10.5 10.0 9.5 9.0 8.5 8.0 7.5 7.0 6.5 6.0 5.5 5.0 4.5 4.0 3.5 3.0 2.5 2.0 1.5 1.0 0.5 0.0
f1 (ppm)
HN
NH2
H2N
N
N
Cl

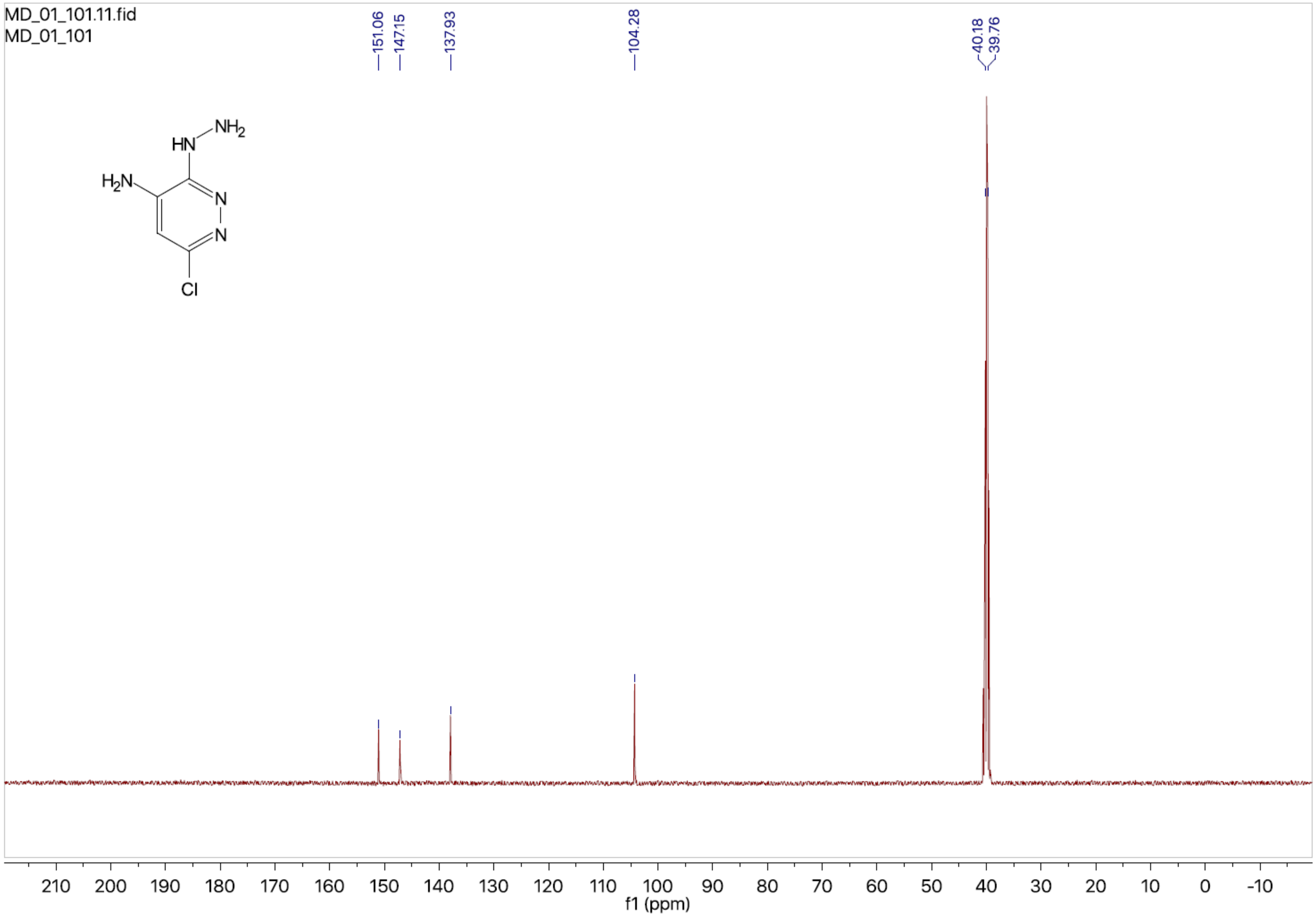
MD_01_101.11.fid
MD_01_101
151.06
147.15
137.93
104.28
40.18
39.76
HN
NH2
H2N
N
N
Cl
210
200
190
180
170
160
150
140
130
120
110
100
90
80
70
60
50
40
30
20
10
0
-10
f1 (ppm)

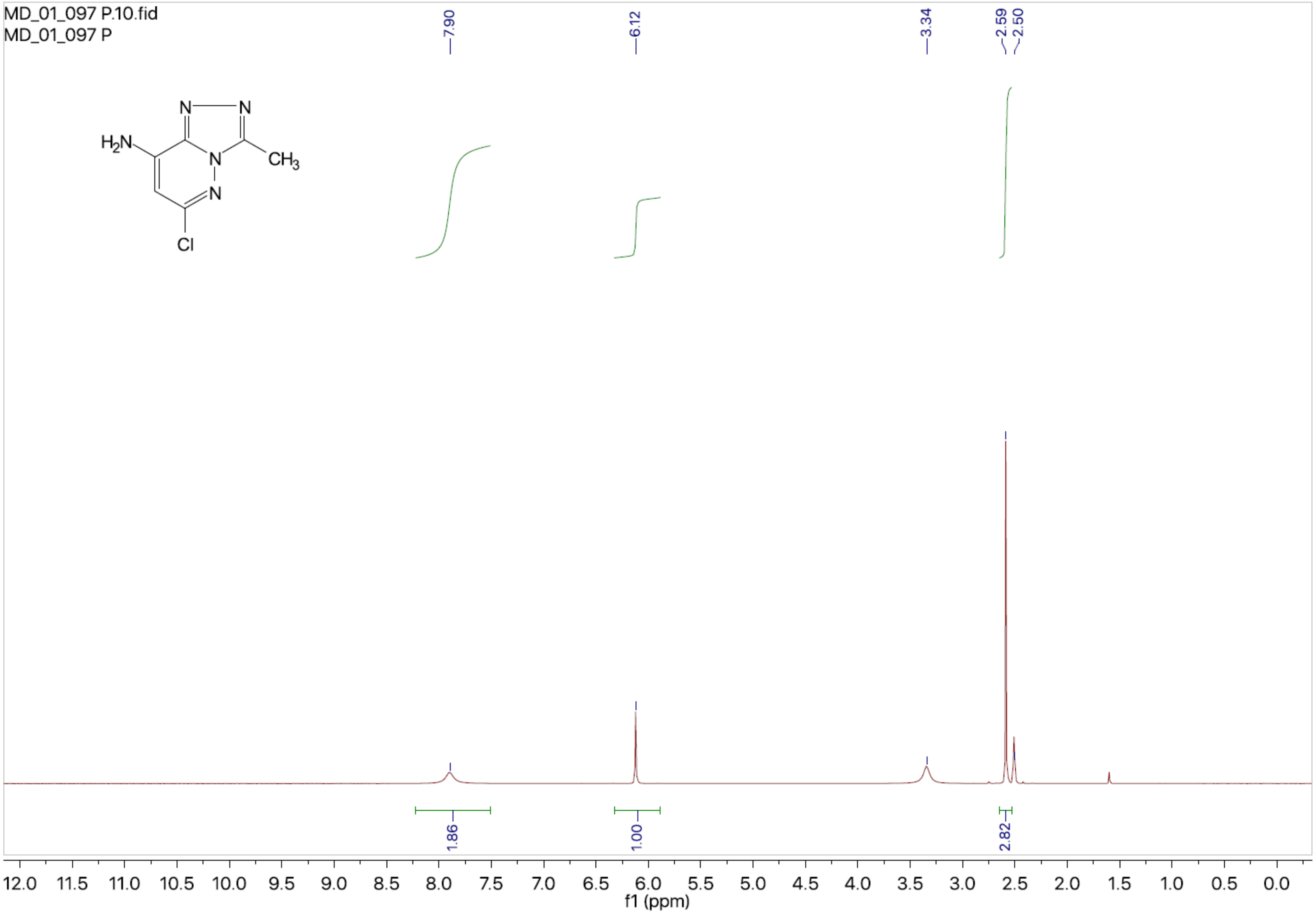
MD_01_097 P.10.fid
MD_01_097 P
7.90
6.12
3.34
2.59
2.50
1.86
1.00
2.82
12.0 11.5 11.0 10.5 10.0 9.5 9.0 8.5 8.0 7.5 7.0 6.5 6.0 5.5 5.0 4.5 4.0 3.5 3.0 2.5 2.0 1.5 1.0 0.5 0.0
f1 (ppm)

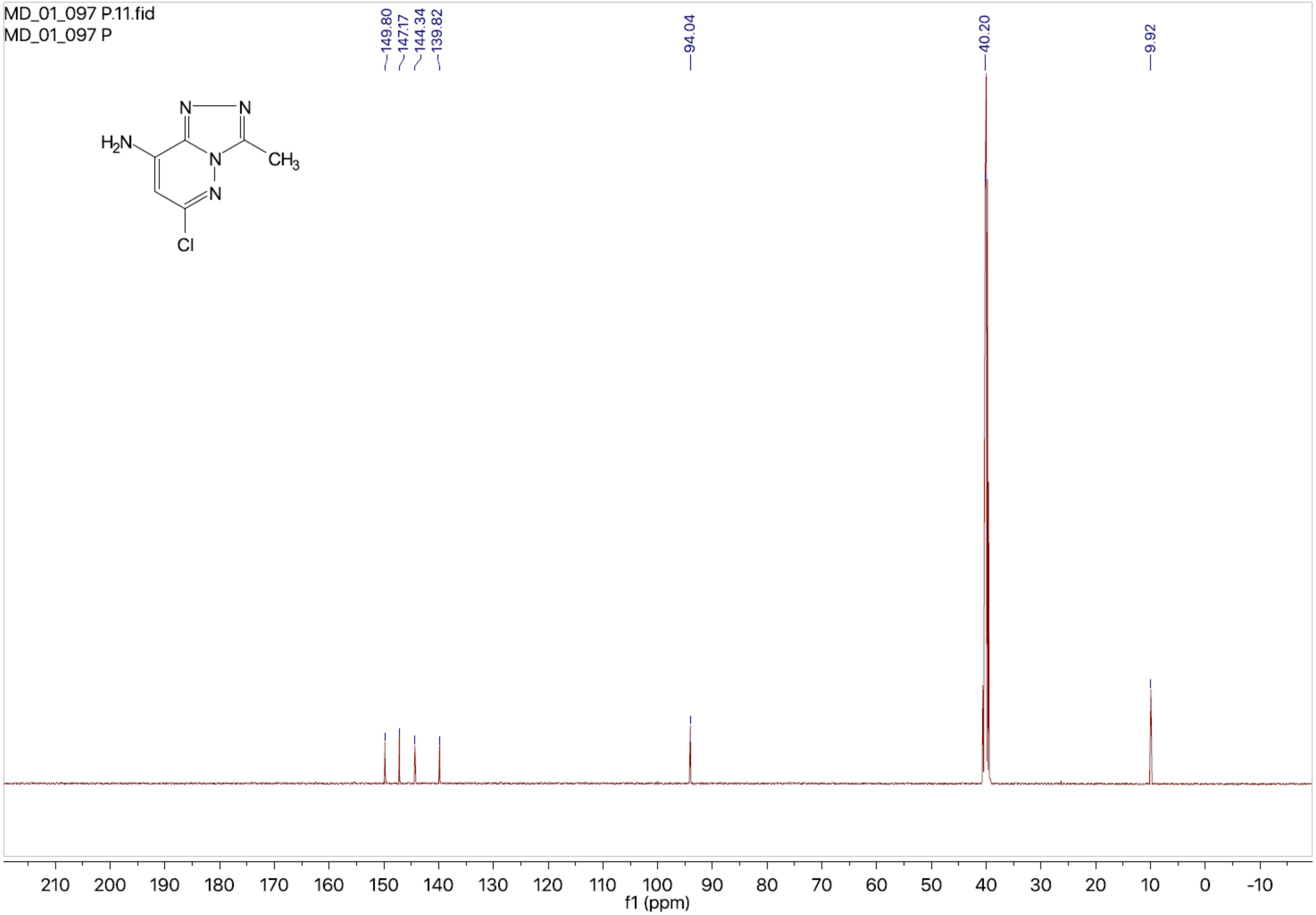

MD_01_097 P.11.fid
MD_01_097 P
149.80
147.17
144.34
139.82
94.04
40.20
9.92
H2N
N
N
N
N
CH3
Cl
210 200 190 180 170 160 150 140 130 120 110 100 90 80 70 60 50 40 30 20 10 0 -10
f1 (ppm)

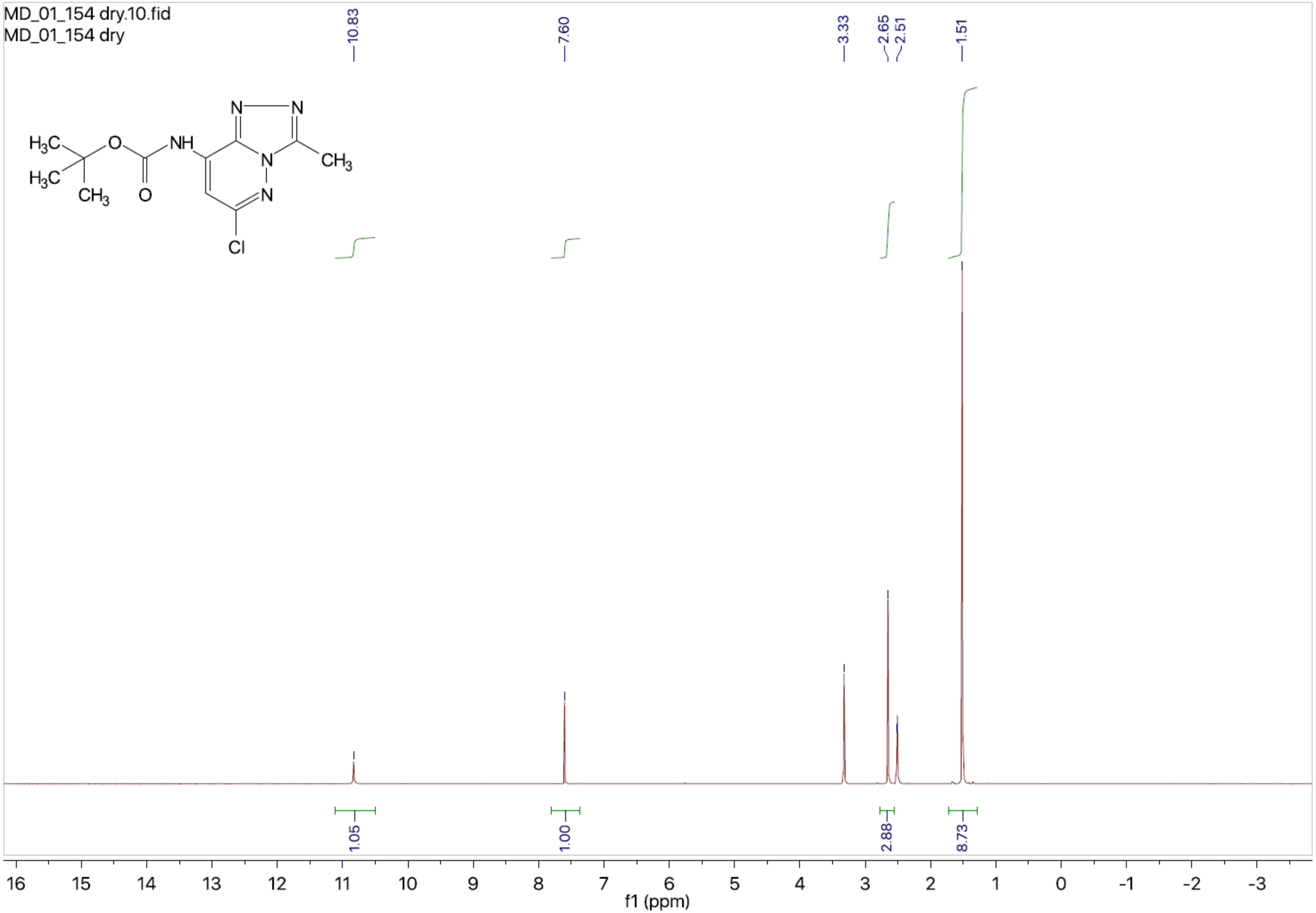
MD_01_154 dry.10.fid
MD_01_154 dry
10.83
7.60
3.33
2.65
2.51
1.51
1.05
1.00
2.88
8.73
f1 (ppm)

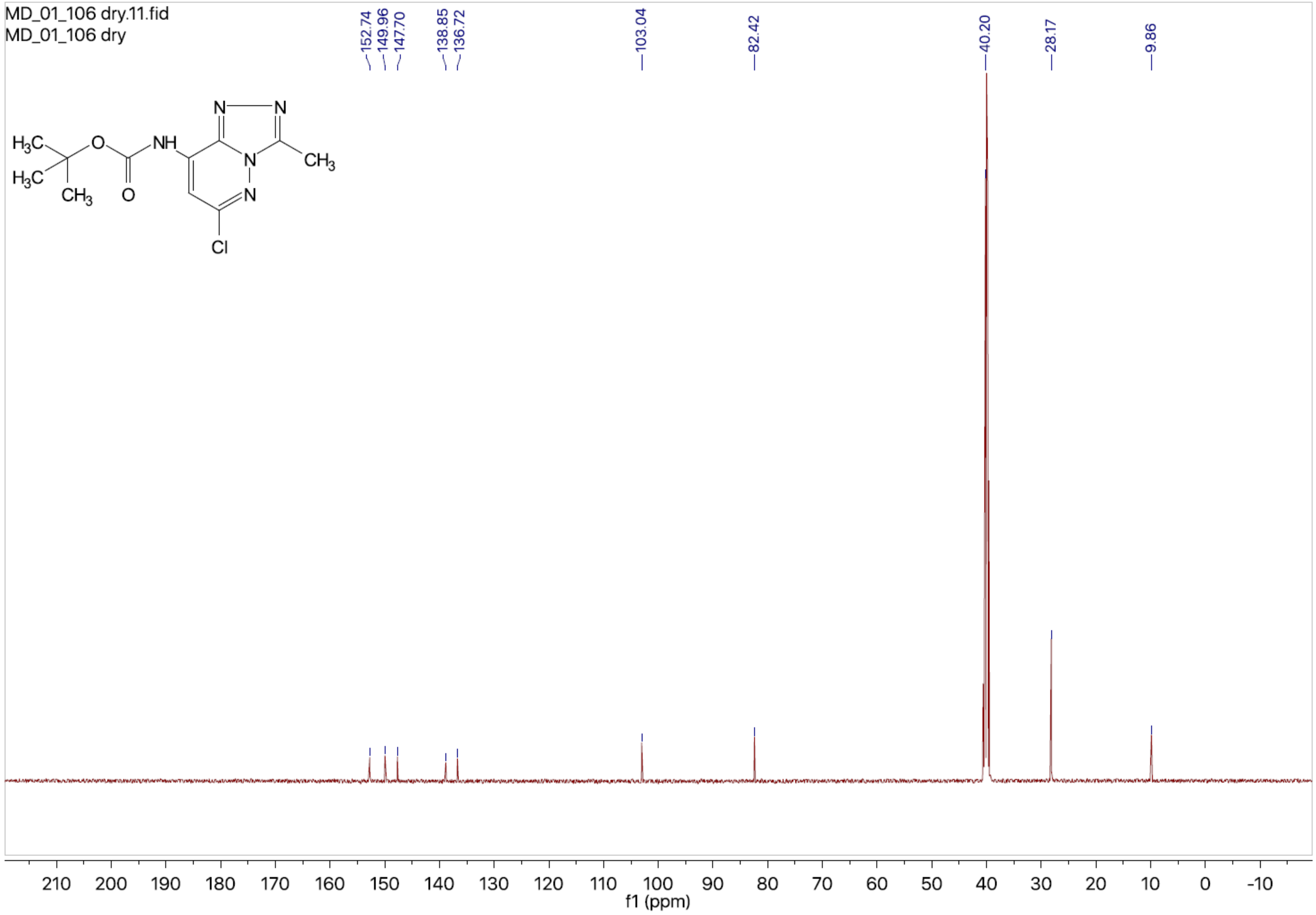
MD_01_106 dry.11.fid
MD_01_106 dry
152.74
149.96
147.70
138.85
136.72
103.04
82.42
40.20
28.17
9.86
H3C
H3C
CH3
O
NH
O
N
N
N
CH3
N
Cl
210 200 190 180 170 160 150 140 130 120 110 100 90 80 70 60 50 40 30 20 10 0 -10
f1 (ppm)

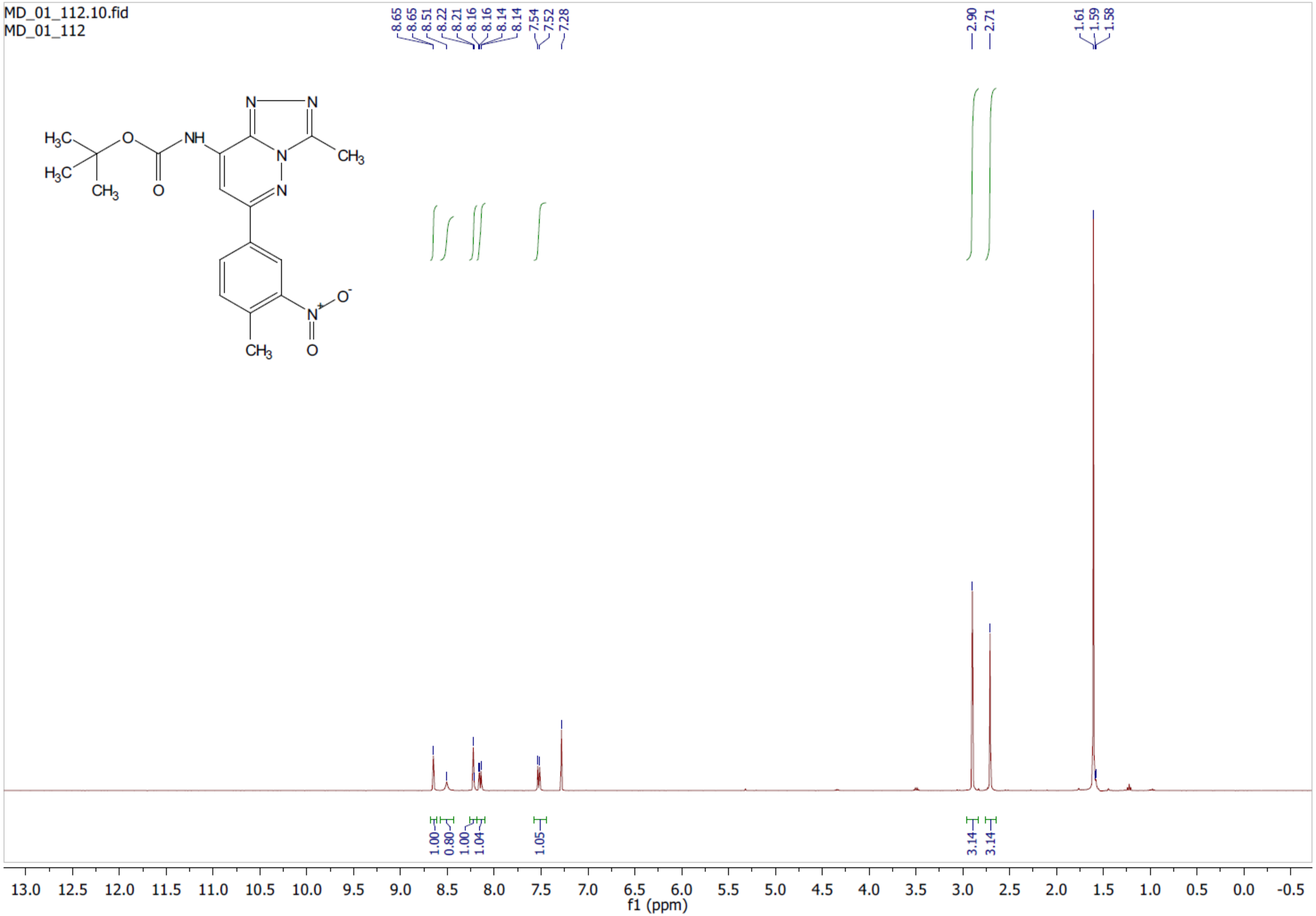

MD_01_112.10.fid
MD_01_112
8.65
8.65
8.51
8.22
8.21
8.16
8.16
8.14
8.14
7.54
7.52
7.28
2.90
2.71
1.61
1.59
1.58
1.00
0.80
1.00
1.04
1.05
3.14
3.14
13.0 12.5 12.0 11.5 11.0 10.5 10.0 9.5 9.0 8.5 8.0 7.5 7.0 6.5 6.0 5.5 5.0 4.5 4.0 3.5 3.0 2.5 2.0 1.5 1.0 0.5 0.0 -0.5
f1 (ppm)

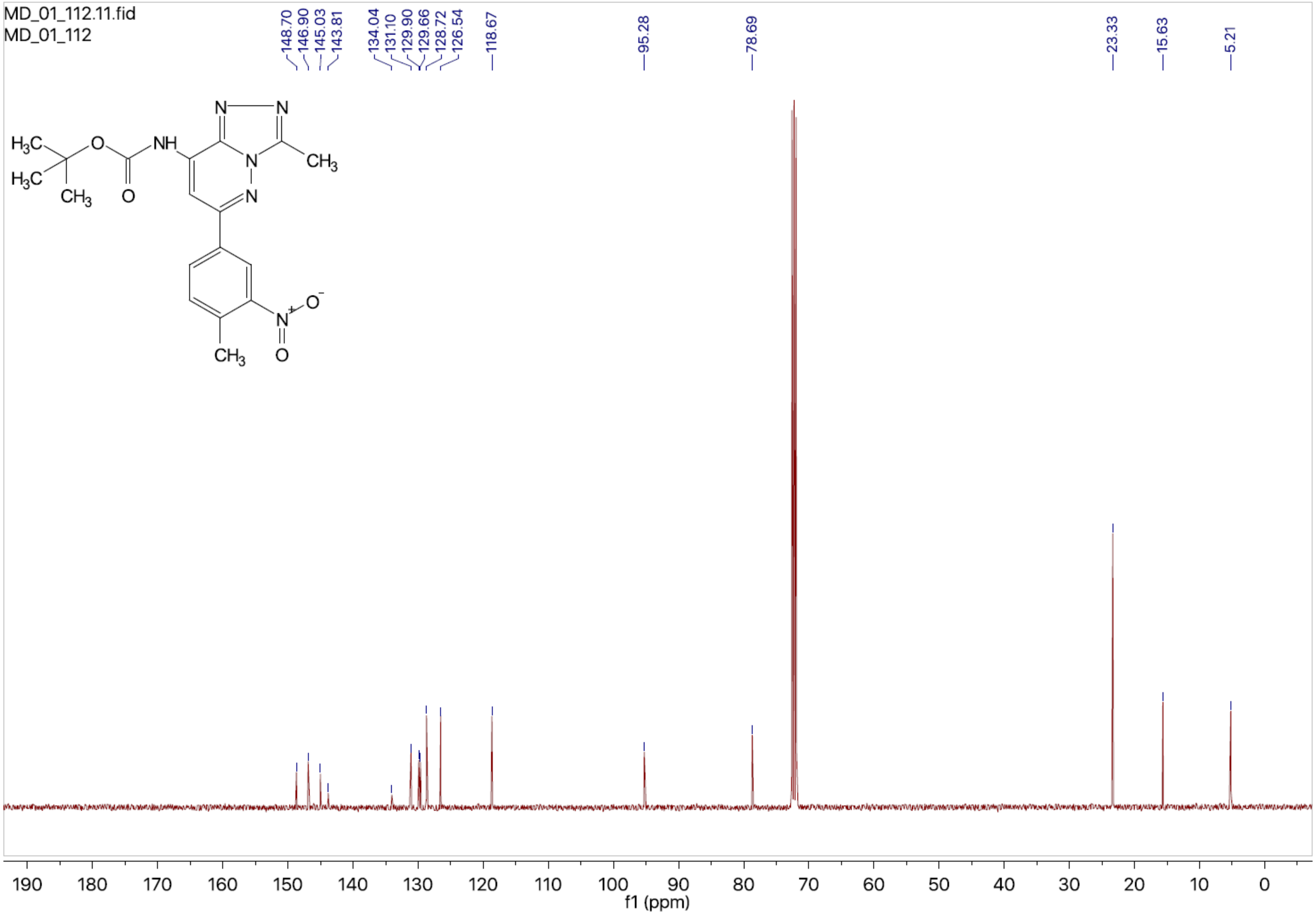
MD_01_112.11.fid
MD_01_112
148.70
146.90
145.03
143.81
134.04
131.10
129.90
129.66
128.72
126.54
118.67
95.28
78.69
23.33
15.63
5.21
190 180 170 160 150 140 130 120 110 100 90 80 70 60 50 40 30 20 10 0
f1 (ppm)

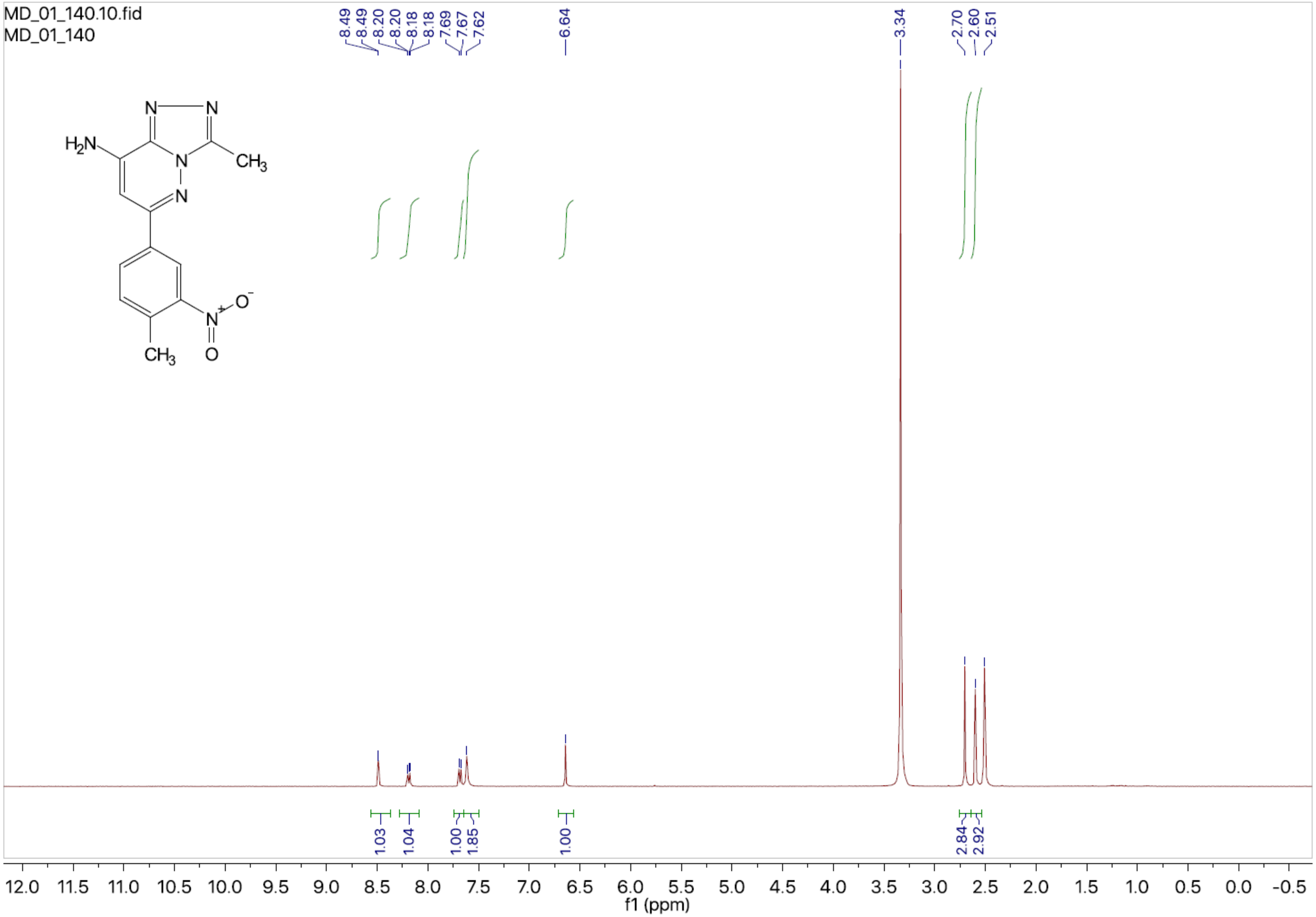

MD_01_140.10.fid
MD_01_140
8.49
8.49
8.20
8.20
8.18
8.18
7.69
7.67
7.62
6.64
3.34
2.70
2.60
2.51
H2N
N
N
N
N
CH3
N+
O-
O
CH3
1.03
1.04
1.00
1.85
1.00
2.84
2.92
12.0 11.5 11.0 10.5 10.0 9.5 9.0 8.5 8.0 7.5 7.0 6.5 6.0 5.5 5.0 4.5 4.0 3.5 3.0 2.5 2.0 1.5 1.0 0.5 0.0 -0.5
f1 (ppm)

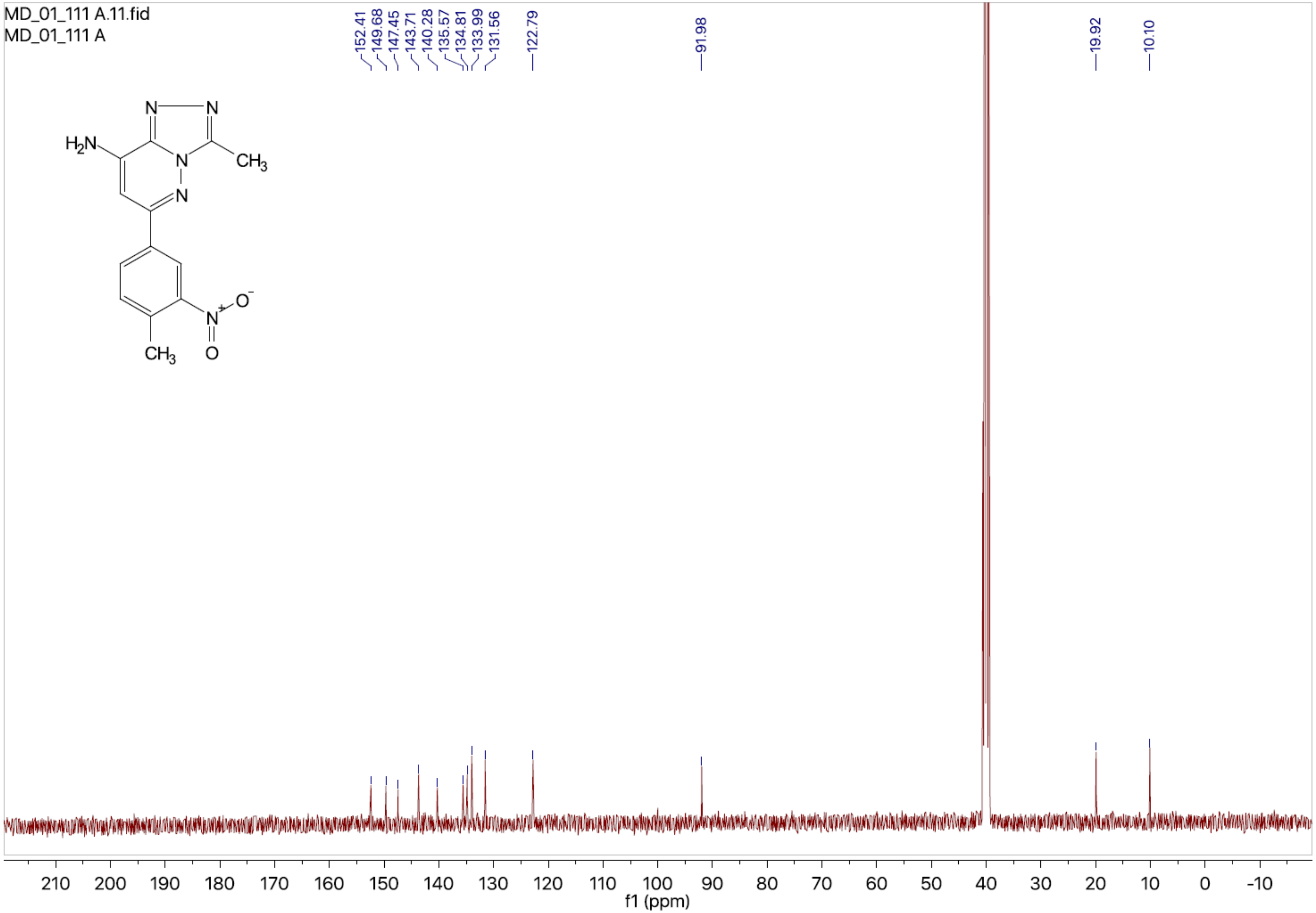
MD_01_111 A.11.fid
MD_01_111 A
152.41
149.68
147.45
143.71
140.28
135.57
134.81
133.99
131.56
122.79
91.98
19.92
10.10
f1 (ppm)

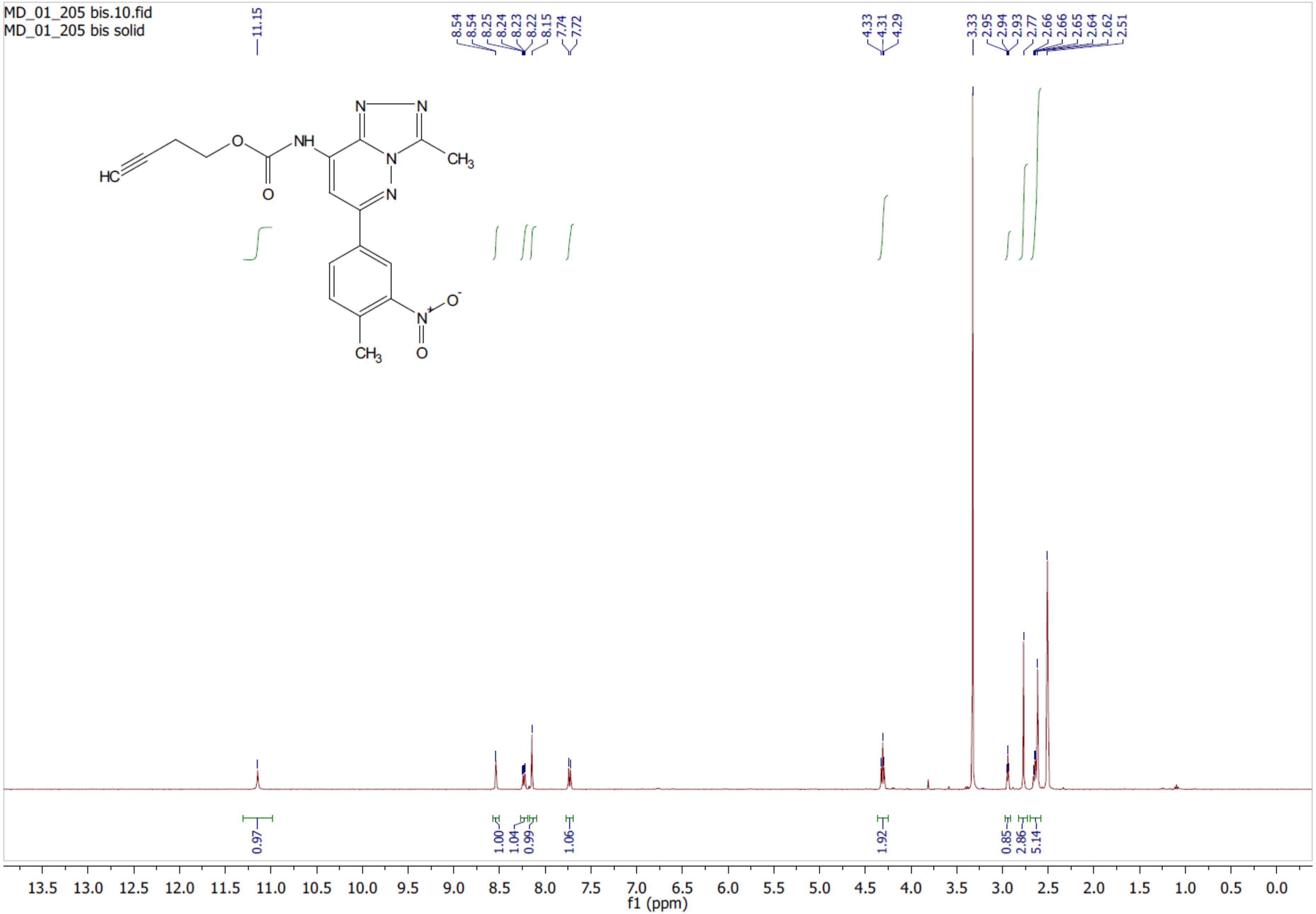

MD_01_205 bis.10.fid
MD_01_205 bis solid
11.15
8.54
8.54
8.25
8.24
8.23
8.22
8.15
7.74
7.72
4.33
4.31
4.29
3.33
2.95
2.94
2.93
2.77
2.66
2.66
2.65
2.64
2.62
2.51
0.97
1.00
1.04
0.99
1.06
1.92
0.85
2.86
5.14
f1 (ppm)

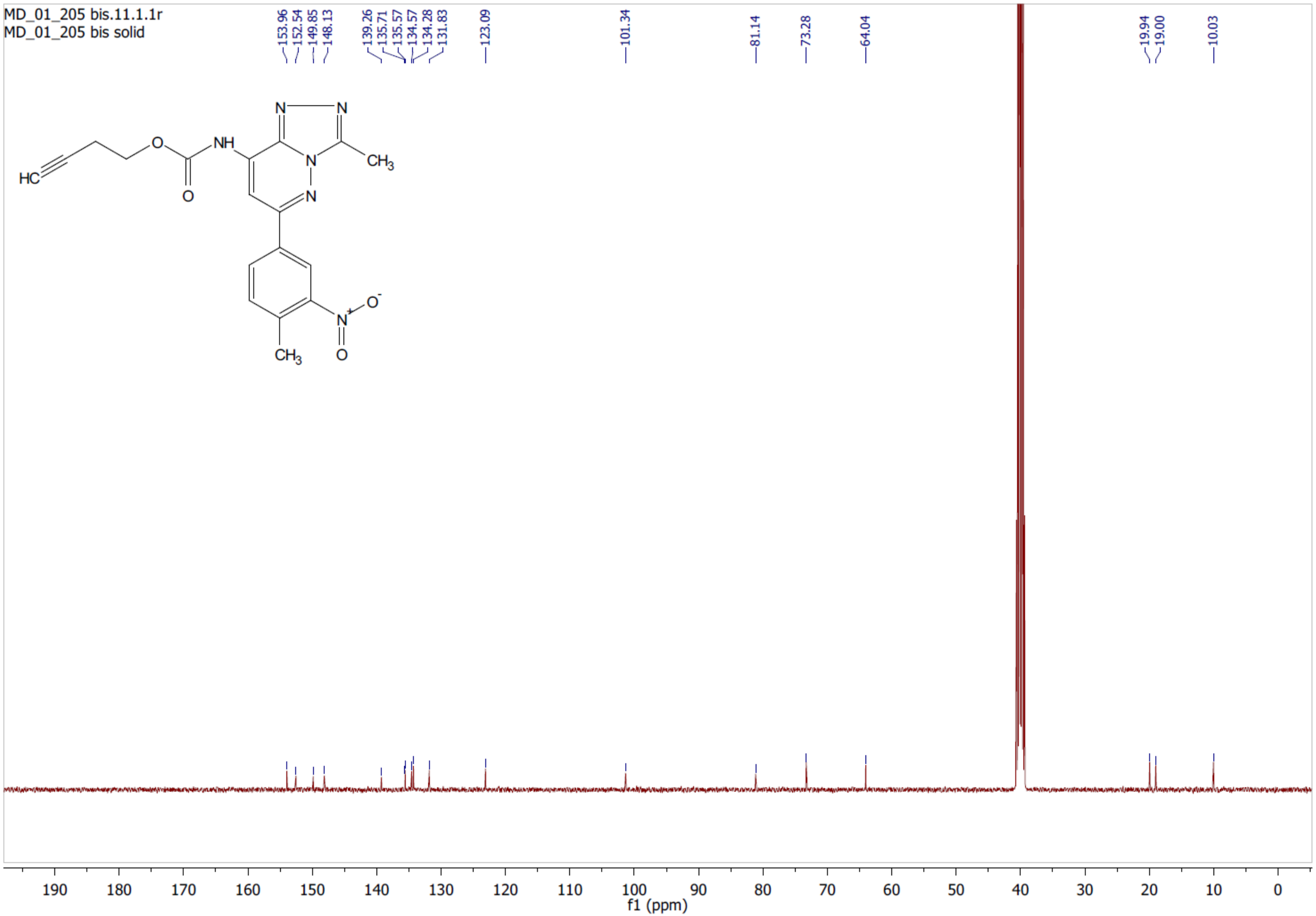
MD_01_205 bis.11.1.1r
MD_01_205 bis solid
153.96
152.54
149.85
148.13
139.26
135.71
135.57
134.57
134.28
131.83
123.09
101.34
81.14
73.28
64.04
19.94
19.00
10.03
HC
O
NH
O
N
N
N
N
CH3
N+
O-
O
CH3
190
180
170
160
150
140
130
120
110
100
90
80
70
60
50
40
30
20
10
0
f1 (ppm)

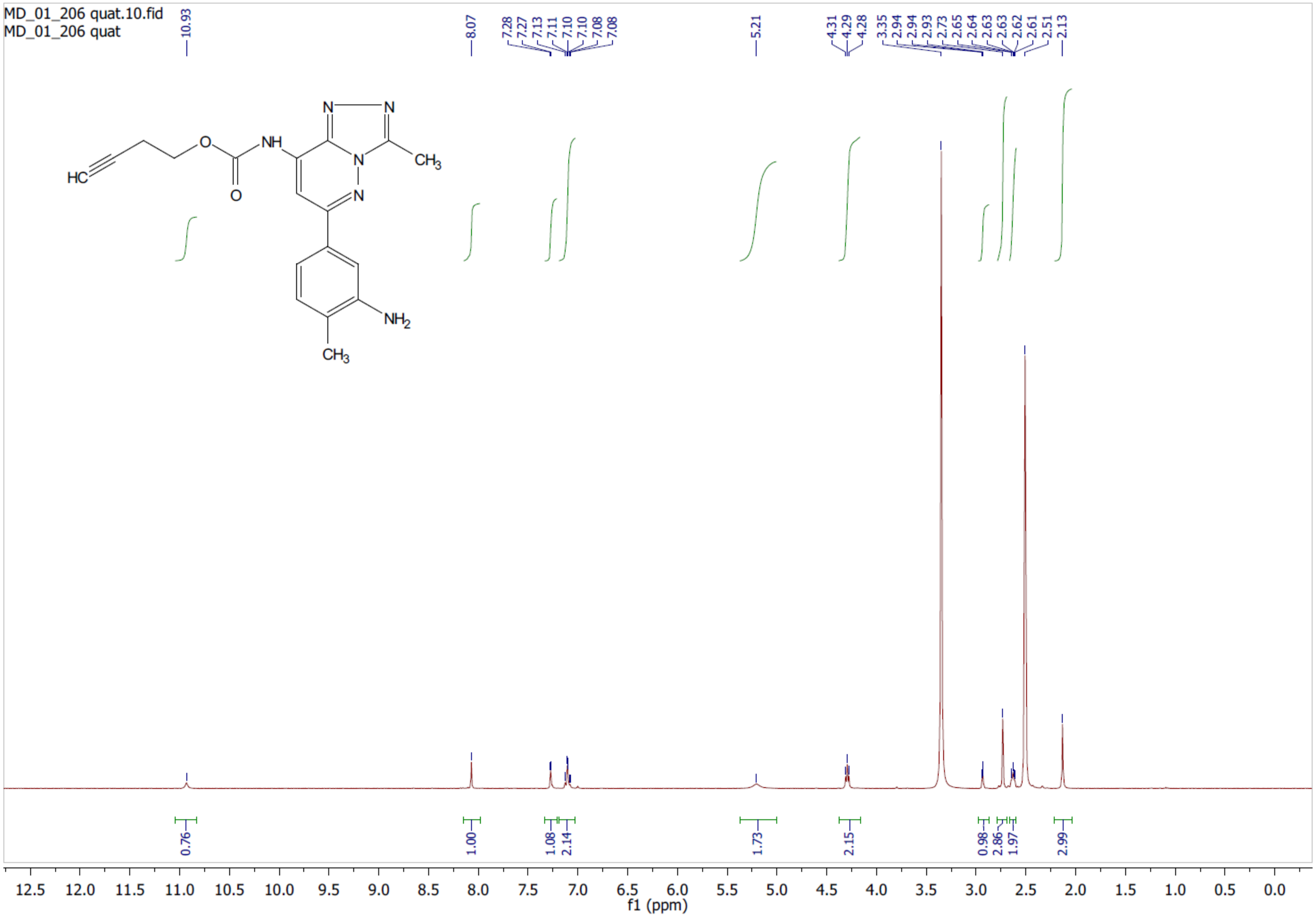
MD_01_206 quat.10.fid
MD_01_206 quat
10.93
8.07
7.28
7.27
7.13
7.11
7.10
7.10
7.08
7.08
5.21
4.31
4.29
4.28
3.35
2.94
2.94
2.93
2.73
2.65
2.64
2.63
2.63
2.62
2.61
2.51
2.13
HC
O
NH
O
N
N
N
N
N
CH3
NH2
CH3
0.76
1.00
1.08
2.14
1.73
2.15
0.98
2.86
1.97
2.99
12.5
12.0
11.5
11.0
10.5
10.0
9.5
9.0
8.5
8.0
7.5
7.0
6.5
6.0
5.5
5.0
4.5
4.0
3.5
3.0
2.5
2.0
1.5
1.0
0.5
0.0
f1 (ppm)

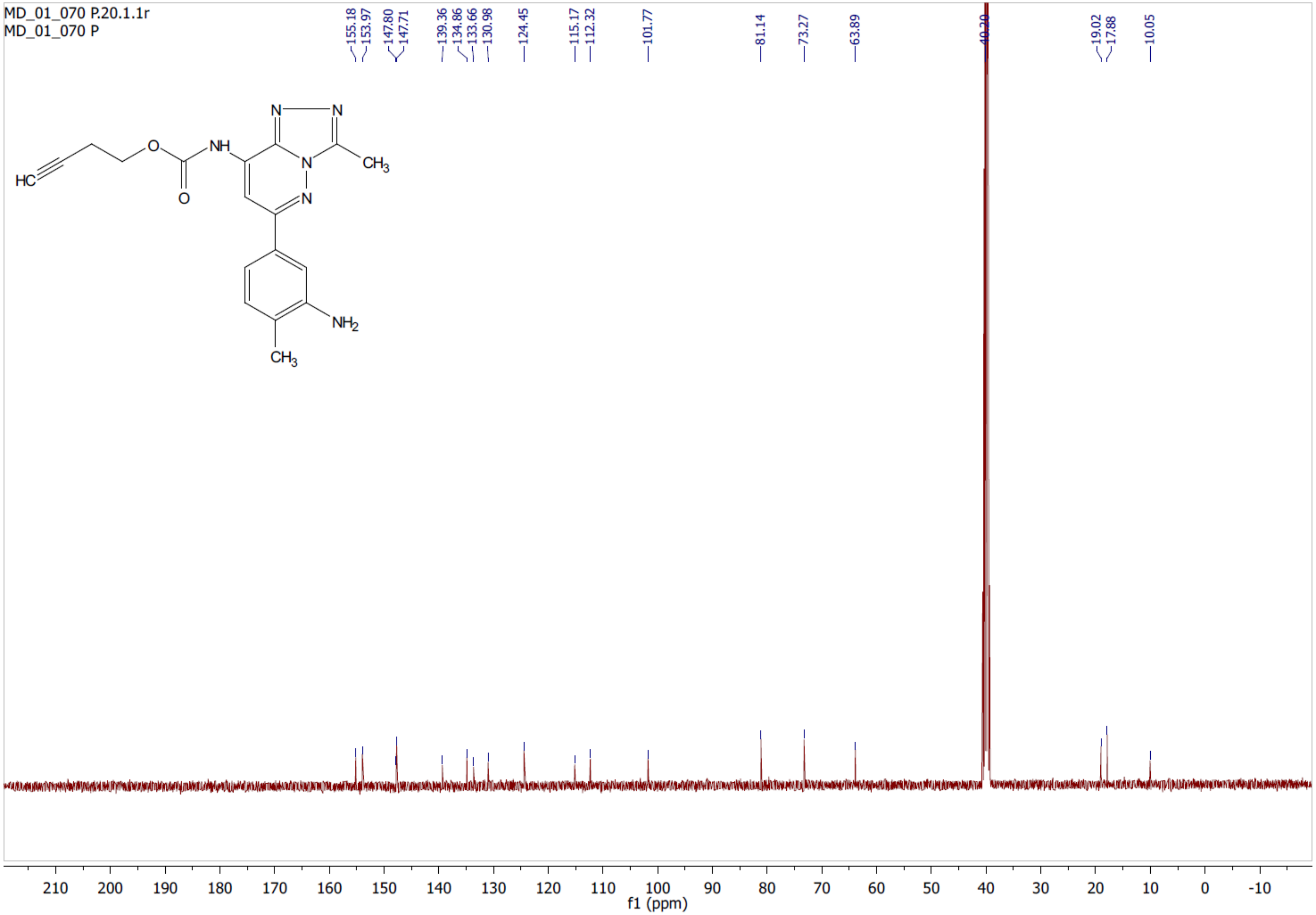
MD_01_070 P.20.1.1r
MD_01_070 P
155.18
153.97
147.80
147.71
139.36
134.86
133.66
130.98
124.45
115.17
112.32
101.77
81.14
73.27
63.89
40.20
19.02
17.88
10.05
f1 (ppm)

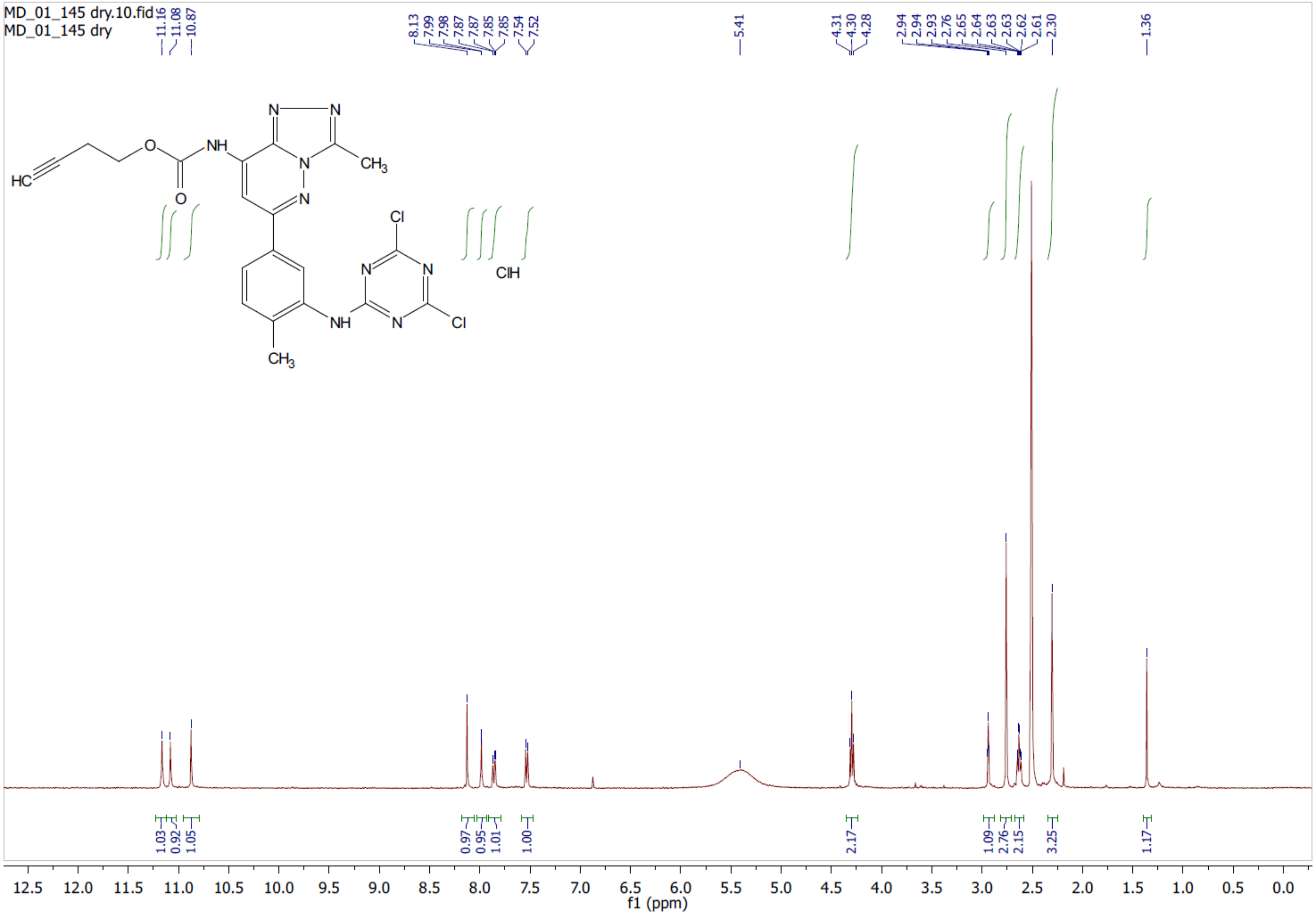
MD_01_145 dry.10.fid
MD_01_145 dry
11.16
11.08
10.87
8.13
7.99
7.98
7.87
7.87
7.85
7.85
7.54
7.52
5.41
4.31
4.30
4.28
2.94
2.94
2.93
2.76
2.65
2.64
2.63
2.63
2.62
2.61
2.30
1.36
HC
O
NH
N
N
N
N
N
CH3
O
Cl
N
N
N
NH
Cl
CH3
ClH
1.03
0.92
1.05
0.97
0.95
1.01
1.00
2.17
1.09
2.76
2.15
3.25
1.17
12.5
12.0
11.5
11.0
10.5
10.0
9.5
9.0
8.5
8.0
7.5
7.0
6.5
6.0
5.5
5.0
4.5
4.0
3.5
3.0
2.5
2.0
1.5
1.0
0.5
0.0
f1 (ppm)

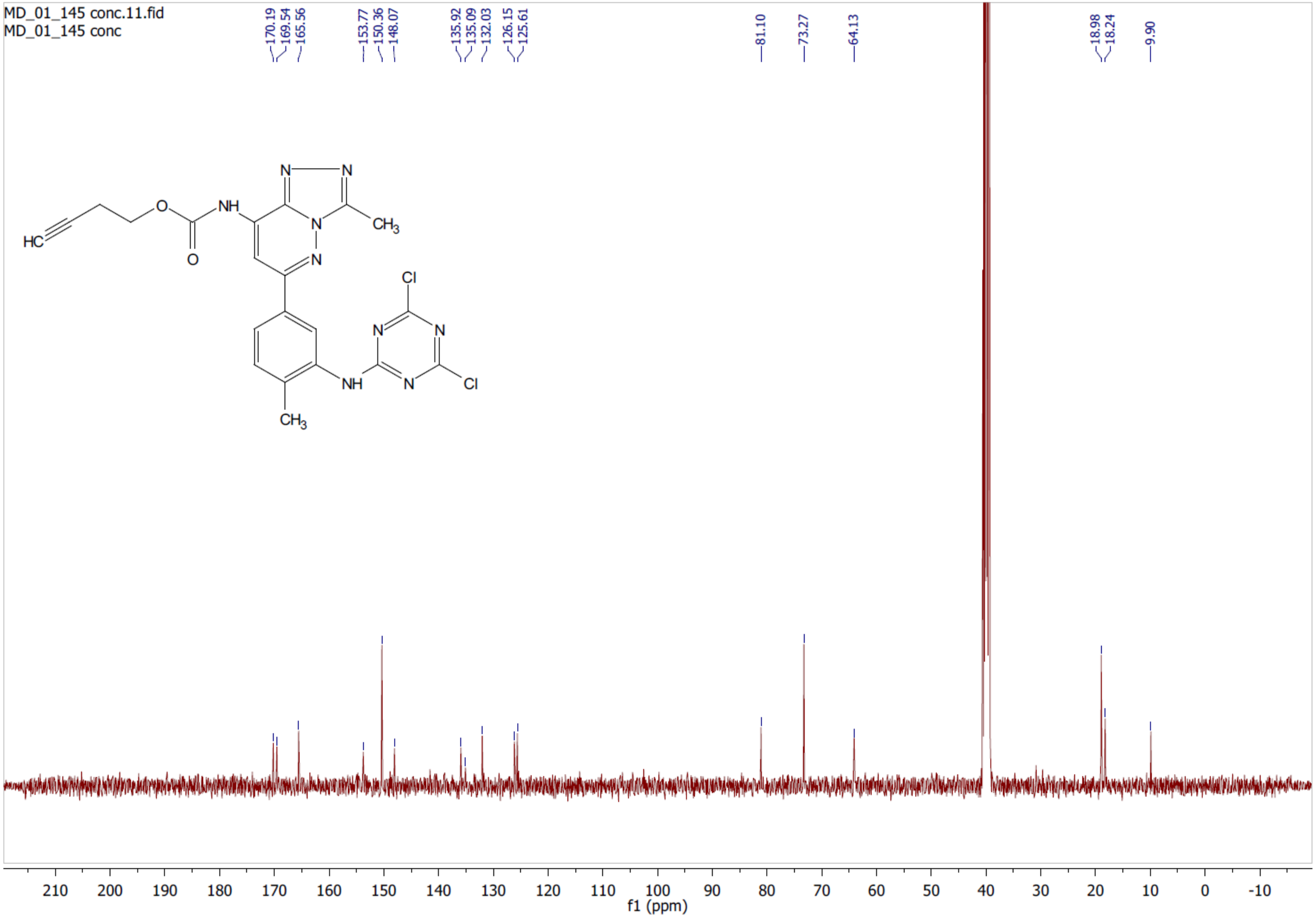

MD_01_145 conc.11.fid
MD_01_145 conc
170.19
169.54
165.56
153.77
150.36
148.07
135.92
135.09
132.03
126.15
125.61
81.10
73.27
64.13
18.98
18.24
9.90
HC
O
NH
N
N
N
N
CH3
O
Cl
N
N
N
NH
Cl
CH3
210 200 190 180 170 160 150 140 130 120 110 100 90 80 70 60 50 40 30 20 10 0 -10
f1 (ppm)

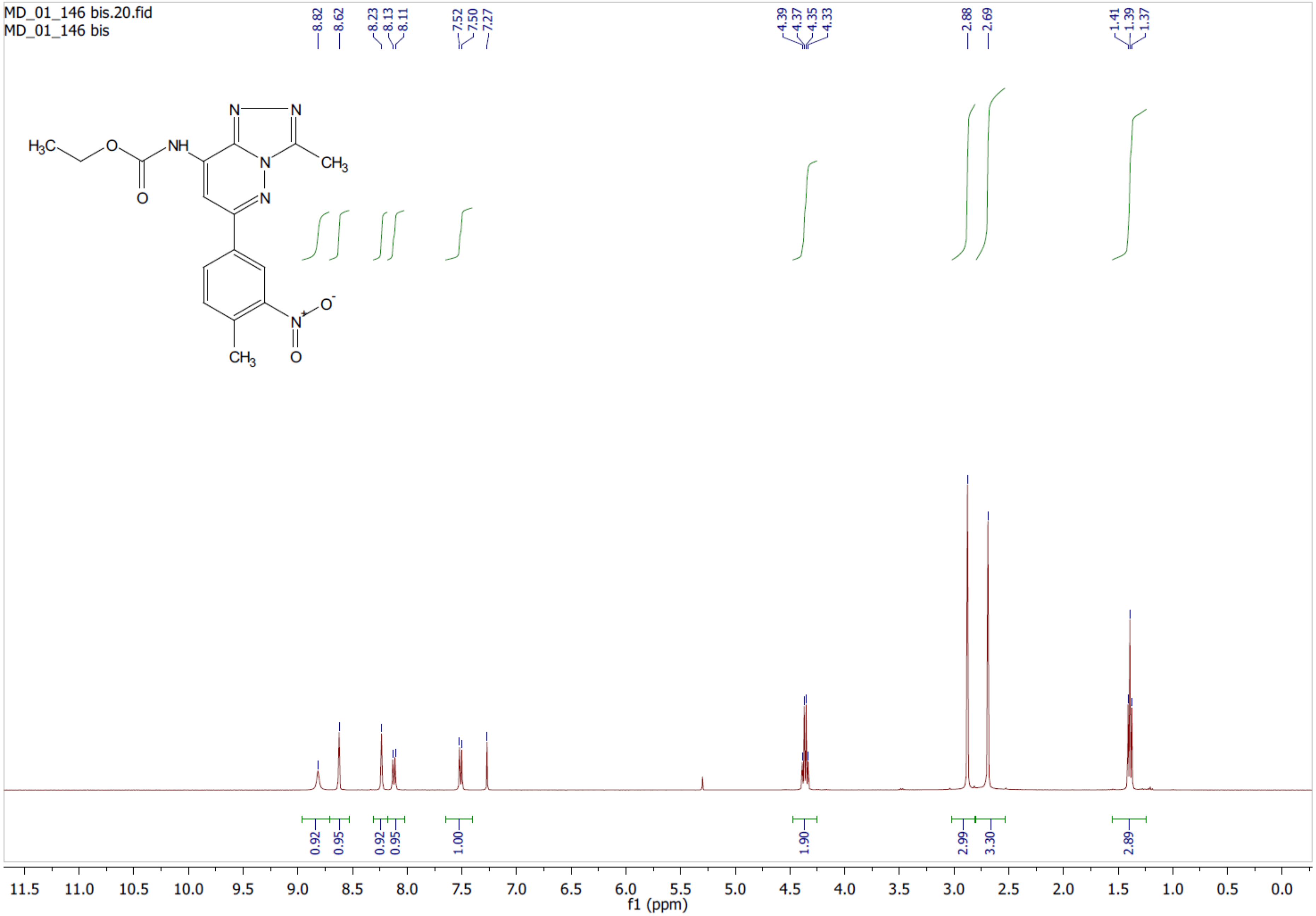
MD_01_146 bis.20.fid
MD_01_146 bis
8.82
8.62
8.23
8.13
8.11
7.52
7.50
7.27
4.39
4.37
4.35
4.33
2.88
2.69
1.41
1.39
1.37
0.92
0.95
0.92
0.95
1.00
1.90
2.99
3.30
2.89
11.5 11.0 10.5 10.0 9.5 9.0 8.5 8.0 7.5 7.0 6.5 6.0 5.5 5.0 4.5 4.0 3.5 3.0 2.5 2.0 1.5 1.0 0.5 0.0
f1 (ppm)

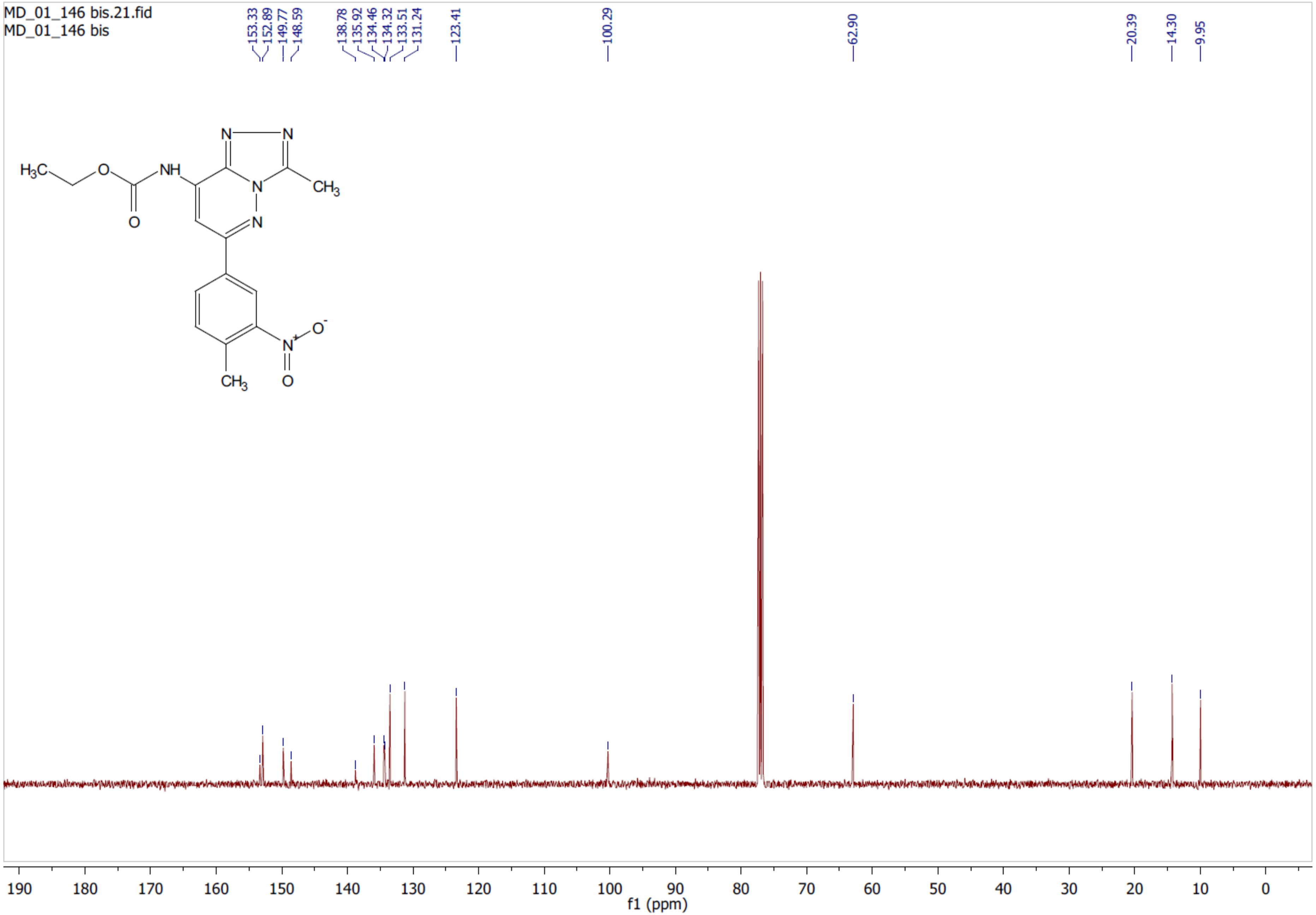

MD_01_146 bis.21.fid
MD_01_146 bis
153.33
152.89
149.77
148.59
138.78
135.92
134.46
134.32
133.51
131.24
123.41
100.29
62.90
20.39
14.30
9.95
f1 (ppm)

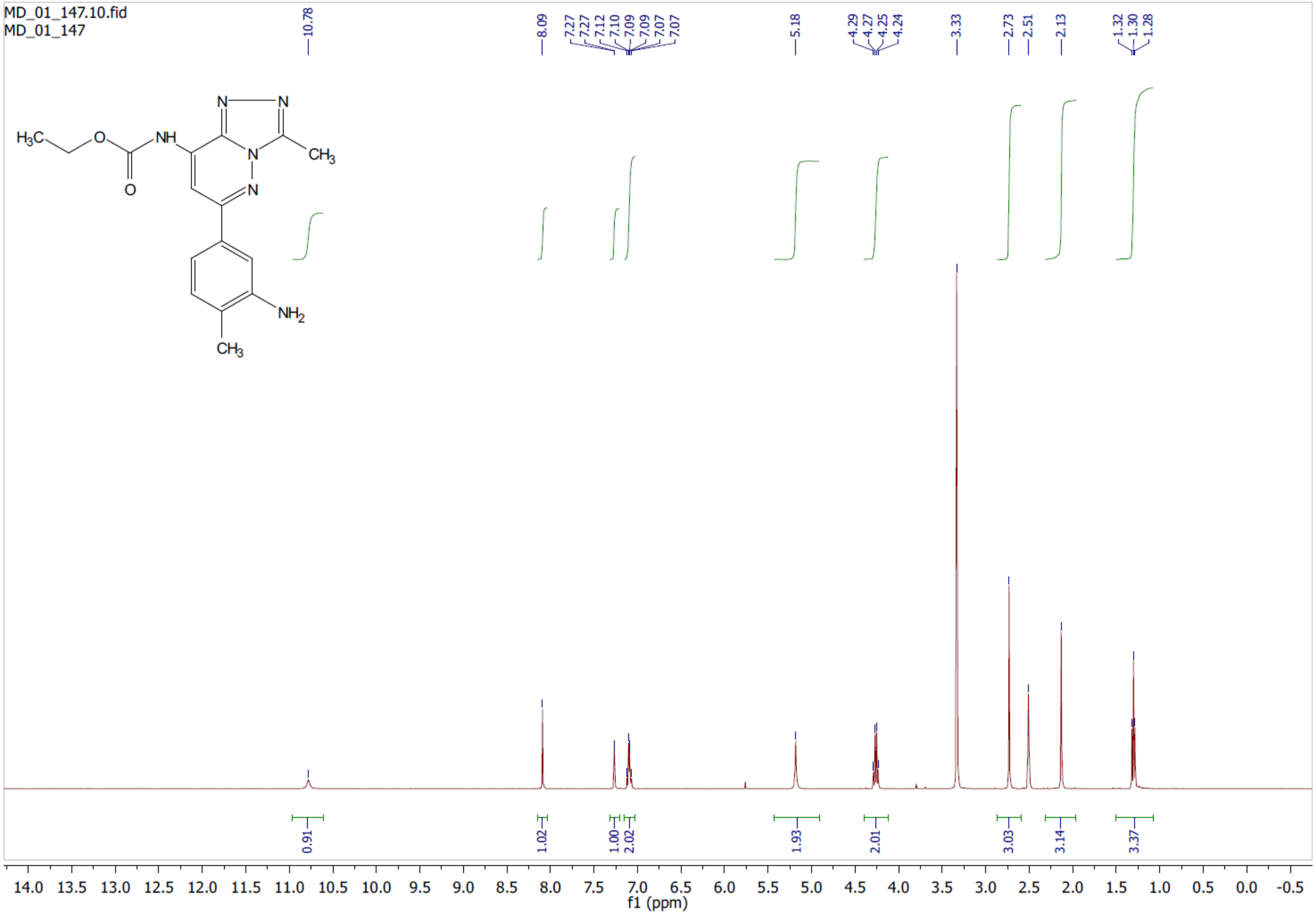
MD_01_147.10.fid
MD_01_147
10.78
8.09
7.27
7.27
7.12
7.10
7.09
7.09
7.07
7.07
5.18
4.29
4.27
4.25
4.24
3.33
2.73
2.51
2.13
1.32
1.30
1.28
0.91
1.02
1.00
2.02
1.93
2.01
3.03
3.14
3.37
f1 (ppm)

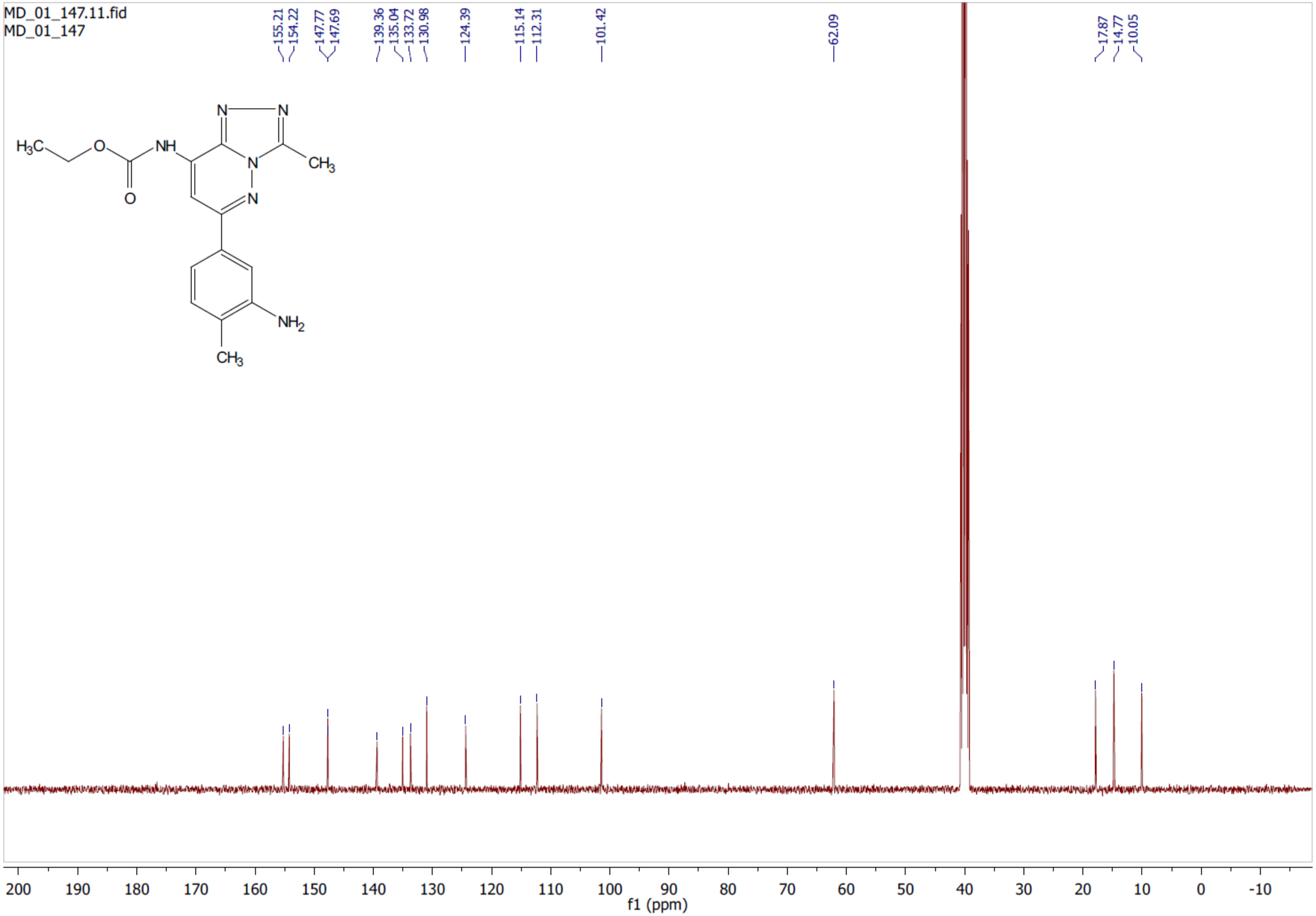
MD_01_147.11.fid
MD_01_147
155.21
154.22
147.77
147.69
139.36
135.04
133.72
130.98
124.39
115.14
112.31
101.42
62.09
17.87
14.77
10.05
H3C
O
NH
O
N
N
N
N
CH3
NH2
CH3
200 190 180 170 160 150 140 130 120 110 100 90 80 70 60 50 40 30 20 10 0 -10
f1 (ppm)

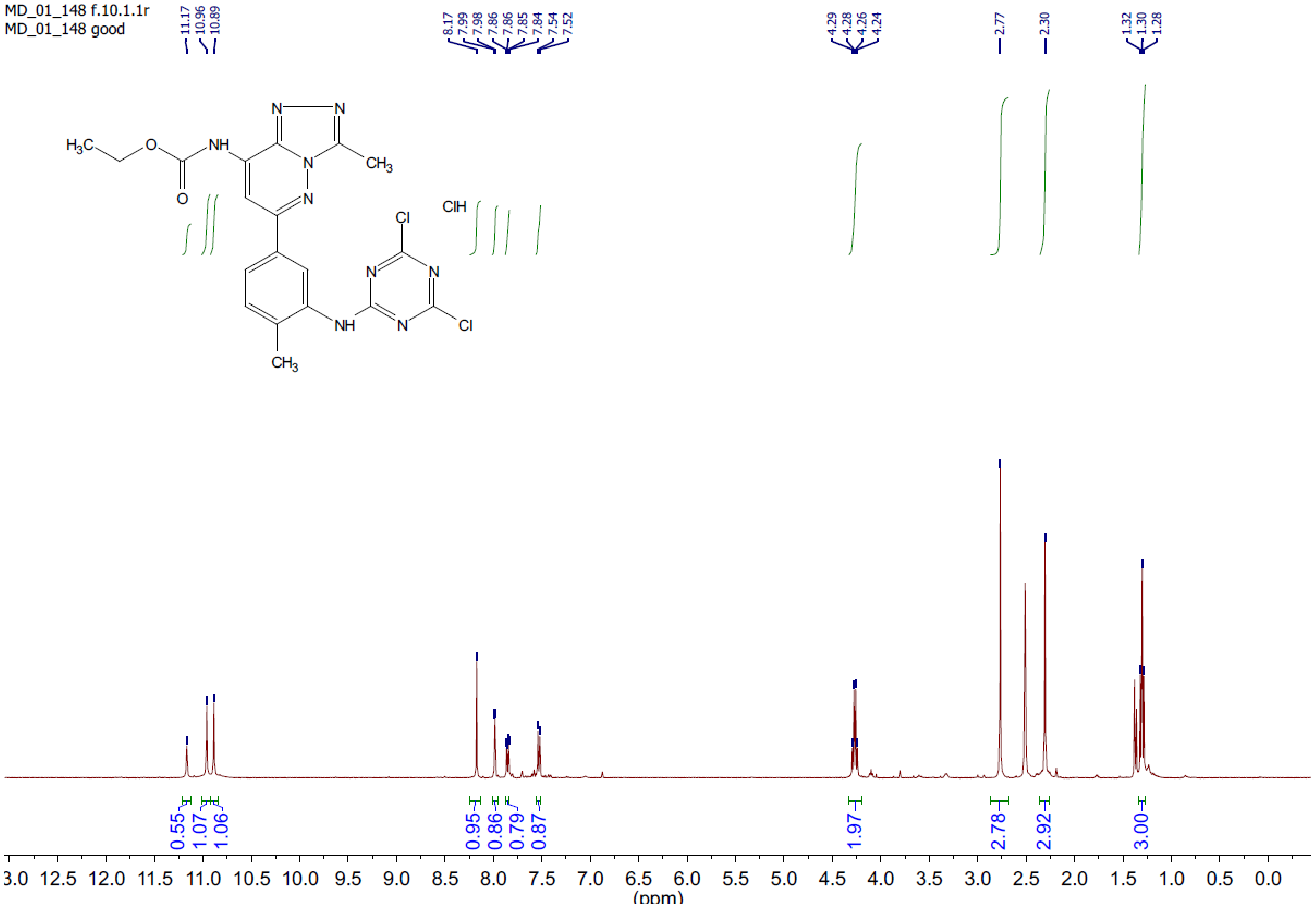

MD_01_148 f.10.1.1r
MD_01_148 good
11.17
10.96
10.89
8.17
7.99
7.98
7.86
7.86
7.85
7.84
7.54
7.52
4.29
4.28
4.26
4.24
2.77
2.30
1.32
1.30
1.28
ClH
0.55
1.07
1.06
0.95
0.86
0.79
0.87
1.97
2.78
2.92
3.00
(ppm)

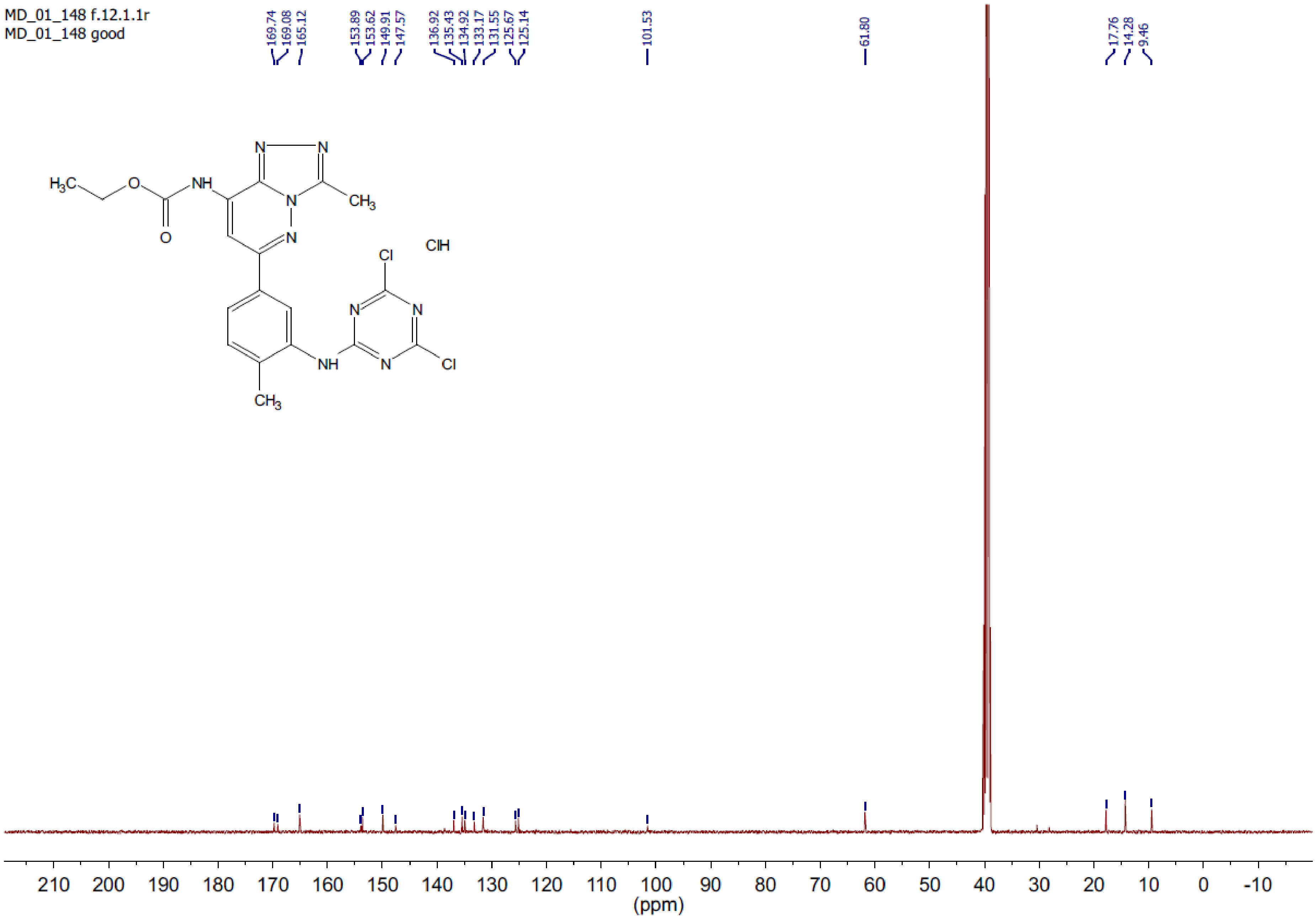
MD_01_148 f.12.1.1r
MD_01_148 good
169.74
169.08
165.12
153.89
153.62
149.91
147.57
136.92
135.43
134.92
133.17
131.55
125.67
125.14
101.53
61.80
17.76
14.28
9.46
ClH
210 200 190 180 170 160 150 140 130 120 110 100 90 80 70 60 50 40 30 20 10 0 -10
(ppm)

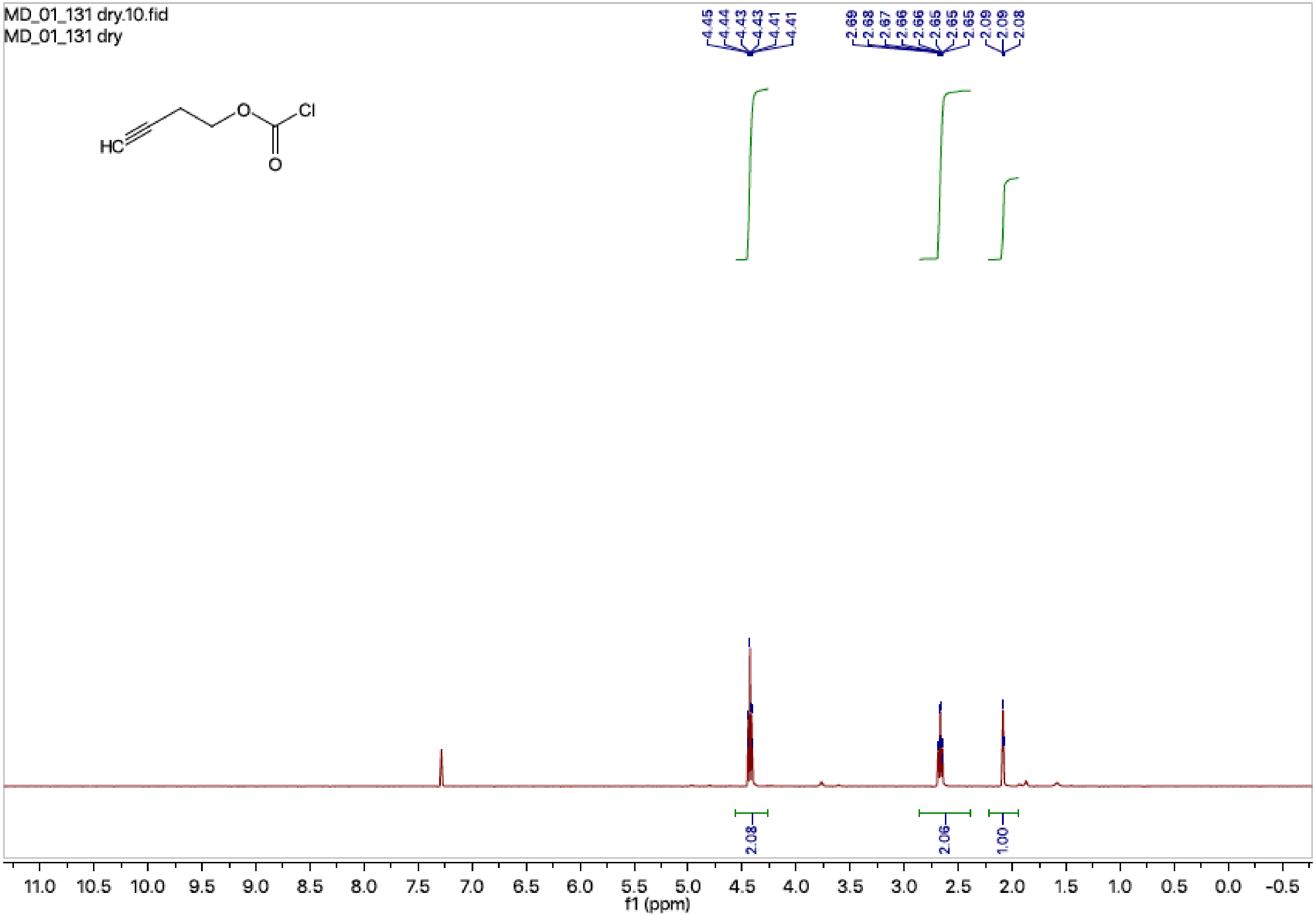

MD_01_131 dry.10.fid
MD_01_131 dry
HC
O
Cl
O
4.45
4.44
4.43
4.43
4.41
4.41
2.69
2.68
2.67
2.66
2.66
2.65
2.65
2.65
2.09
2.09
2.08
2.08
2.06
1.00
11.0 10.5 10.0 9.5 9.0 8.5 8.0 7.5 7.0 6.5 6.0 5.5 5.0 4.5 4.0 3.5 3.0 2.5 2.0 1.5 1.0 0.5 0.0 -0.5
f1 (ppm)

5. Rapidfire Mass Spectrometry

Determining the selectivity of Bromotriazine against BRDs belonging to different families.
Reaction conditions: 30 µM probe, 3 µM recombinant BRD in buffer (20 mM HEPES, 200 mM NaCl). Incubations: 1.5 hours at 37 °C.
Each experiment was conducted in triplicates.

	Deconvoluted Area§							Covalent Modification(%)	
Recombinant Bromodomain	Modified BRD	SEM	MW	Unmodified BRD	SEM	MW	Total Area	Modified BRD	Unmodified BRD
BRD4(1)	1114097	164682	15545.60	2977325	393624	15083.88	4091422	27.2	72.8
BRD4(2)	1446711	100901	15498.52	8496233	695755	15036.63	9942944	14.5	85.5
BRD3(2)	2284981	31519	13746.64	8103269	191522	13284.88	10388250	22.0	78.0
ATAD2A	181088	10751	15892.62	4338485	195511	15430.95	4519573	4.0	96.0
SMARCA2B	0	0		7946558	247416	14358.01	7946558	0	100
PB1(5)	17124	17124	15088.26	5085268	356467	14626.36	5102392	0.3	99.7
CREBBP	228574	62605	14669.46	4738909	250376	14207.89	4967484	4.6	95.4
TIF1a	113262	2750	21839.29	5913572	336762	21378.04	6026833	1.9	98.1
CECR2A	6180803	146286	14171.91	0	0		6180803	100	0
BRD1	82856	9069	15317.31	10037190	399369	14855.44	10120047	0.8	99.2
BRD9	4221995	350698	14689.06	1249599	49637	14227.17	5471594	77.1	22.9
BRPF1B	95674	5896	14149.19	2977325	393624	13687.16	6901717	1.4	98.6
BRD7	2568623	230824	13707.32	426580	48093	13245.22	2995203	85.7	14.2
PCAF	378311	9370	17077.48	3966480	50981	16616.16	4344791	8.7	91.3
TAF1(1)	488203	2930	18074.82	594004	3071	17613.04	1082207	45.1	54.9
TAF1(2)	501616	14676	18793.64	610873	20235	18331.54	1112489	45.1	54.9
TAF1L(1)	201753	19454	17238.57	1679168	149522	16777.20	1880921	10.7	89.3
TAF1L(2)	398490	44659	18462.01	1411734	144781	18000.13	1810223	22.0	78.0
GCN5L2A	0	0		1517711	716125	15975.39	1517711	0	100

§Deconvolued Areas are reported as average values of three replicates.

5.1 Recombinant Bromodomains Sequences

Bromodomains were produced and purified as described previously.[6]

Recombinant Bromodomain	Amino acid sequence	MW
BRD4(1)	SMNPPPPETSNPNKPKRQTNQLQYLLRVVLKTLWKHQFAWPFQQPVDAVKLNLPDYYKIIKTPMDMGTIKKRLENNYYWNAQECIQ DFNTMFTNCYIYNKPGDDIVLMAEALEKLFLQKINELPTEE	**15083**
BRD4(2)	SMKDVPDSQQHPAPEKSSKVSEQLKCCSGILKEMFAKKHAAYAWPFYKPVDVEALGLHDYCDIIKHPMDMSTIKSKLEAREYRDAQEF GADVRLMFSNCYKYNPPDHEVVAMARKLQDVFEMRFAKMPDE	**15036**
BRD3(2)	SMGKLSEHLRYCDSILREMLSKKHAAYAWPFYKPVDAEALELHDYHDIIKHPMDLSTVKRKMDGREYPDAQGFAADVRLMFSNCYKY NPPDHEVVAMARKLQDVFEMRFAKMP	**13284**
ATAD2A	SMQEEDTFRELRIFLRNVTHRLAIDKRFRVFTKPVDPDEVPDYVTVIKQPMDLSSVISKIDLHKYLTVKDYLRDIDLICSNALEYNPDRDPG DRLIRHRACALRDTAYAIIKEELDEDFEQLCEEIQESR	**15430**
SMARCA2B	SMAEKLSPNPPKLTKQMNAIIDTVINYKDSSGRQLSEVFIQLPSRKELPEYYELIRKPVDFKKIKERIRNHKYRSLGDLEKDVMLLCHNAQT FNLEGSQIYEDSIVLQSVFKSARQKIAKEEE	**14357**
PB1(5)	SMSGISPKKSKYMTPMQQKLNEVYEAVKNYTDKRGRRLSAIFLRLPSRSELPDYYLTIKKPMDMEKIRSHMMANKYQDIDSMVEDFV MMFNNACTYNEPESLIYKDALVLHKVLLETRRDLEGD	**14626**
CREBBP	SMRKKIFKPEELRQALMPTLEALYRQDPESLPFRQPVDPQLLGIPDYFDIVKNPMDLSTIKRKLDTGQYQEPWQYVDDVWLMFNNAW LYNRKTSRVYKFCSKLAEVFEQEIDPVMQSLG	**14207**
TIF1a	SMNEDWCAVCQNGGELLCCEKCPKVFHLSCHVPTLTNFPSGEWICTFCRDLSKPEVEYDCDAPSHNSEKKKTEGLVKLTPIDKRKCERLL LFLYCHEMSLAFQDPVPLTVPDYYKIIKNPMDLSTIKKRLQEDYSMYSKPEDFVADFRLIFQNCAEFNEPDSEVANAGIKLENYFEELLKNL YPSSKGGYGLNDIFEAQKIEWHE	**21377**
CECR2A	SMTKDLFELDDDFTAMYKVLDVVKAHKDSWPFLEPVDESYAPNYYQIIKAPMDISSMEKKLNGGLYCTKEEFVNDMKTMFRNCRKYN GESSEYTKMSDNLERCFHRAMMKHFPGED	**13708**
BRD1	SMRLTPLTVLLRSVLDQLQDKDPARIFAQPVSLKEVPDYLDHIKHPMDFATMRKRLEAQGYKNLHEFEEDFDLIIDNCMKYNARDTVFY RAAVRLRDQGGVVLRQARREVDSIGLEEASGMHLPERPS	**14855**
BRD9	SMLKLSAENESTPIQQLLEHFLRQLQRKDPHGFFAFPVTDAIAPGYSMIIKHPMDFGTMKDKIVANEYKSVTEFKADFKLMCDNAMTY NRPDTVYYKLAKKILHAGFKMMSKERLLALKRSMS	**14227**
BRD9 His tag	MHHHHHHSSGVDLGTENLYFQSMLKLSAENESTPIQQLLEHFLRQLQRKDPHGFFAFPVTDAIAPGYSMIIKHPMDFGTMKDKIVANE YKSVTEFKADFKLMCDNAMTYNRPDTVYYKLAKKILHAGFKMMSKERLLALKRSMS	**16692**
BRPF1B	SMEMQLTPFLILLRKTLEQLQEKDTGNIFSEPVPLSEVPDYLDHIKKPMDFFTMKQNLEAYRYLNFDDFEEDFNLIVSNCLKYNAKDTIFY RAAVRLREQGGAVLRQARRQAEKMG	**13687**

BRD7	SMEEVEQTPLQEALNQLMRQLQRKDPSAFFSFPVTDFIAPGYSMIIKHPMDFSTMKEKIKNNDYQSIEELKDNFKLMCTNAMIYNKPETIYYKAAKKLLHSGMKILSQERIQS	**13245**
PCAF	MHHHHHHSSGVDLGTENLYFQSMGKEKSKEPRDPDQLYSTLKSILQQVKSHQSAWPFMEPVKRTEAPGYYEVIRFPMDLKTMSERLKNRYYVSKKLFMADLQRVFTNCKEYNPPESEYYKCANILEKFFFSKIKEAGLID	**16616**
TAF1(1)	MHHHHHHSSGVDLGTENLYFQSMRRTDPMVTLSSILESIINDMRDLPNTYPFHTPVNAKVVKDYYKIITRPMDLQTLRENVRKRLYPSREEFREHLELIVKNSATYNGPKHSLTQISQSMLDLCDEKLKEKEDKLARLEKAINPLLDDDD	**17613**
TAF1(2)	MHHHHHHSSGVDLGTENLYFQSMDDDQVAFSFILDNIVTQKMMAVPDSWPFHHPVNKKFVPDYYKVIVNPMDLETIRKNISKHKYQSRESFLDDVNLILANSVKYNGPESQYTKTAQEIVNVCYQTLTEYDEHLTQLEKDICTAKEAALEEAELESLD	**18331.5**
TAF1L(1)	MHHHHHHSSGVDLGTENLYFQSMVTLSSILESIINDMRDLPNTHPFHTPVNAKVVKDYYKIITRPMDLQTLRENVRKCLYPSREEFREHLELIVKNSATYNGPKHSLTQISQSMLDLCDEKLKEKEDKLARLEKAINPLLDDDD	**16777**
TAF1L(2)	MHHHHHHSSGVDLGTENLYFQSMQVAFSFILDNIVTQKMMAVPDSWPFHHPVNKKFVPDYYKMIVNPVDLETIRKNISKHKYQSRESFLDDVNLILANSVKYNGPESQYTKTAQEIVNICYQTITEYDEHLTQLEKDICTAKEAALEEAELESLD	**18000**
GCN5L2A	MHHHHHHSSGVDLGTENLYFQSMELKDPDQLYTTLKNLLAQIKSHPSAWPFMEPVKKSEAPDYYEVIRFPIDLKTMTERLRSRYYVTRKLFVADLQRVIANCREYNPPDSEYCRCASALEKFFYFKLKEGGLIDK	**15975**

(6) Filippakopoulos, P.; Picaud, S.; Mangos, M.; Keates, T.; Lambert, J. P.; Barsyte-Lovejoy, D.; Felletar, I.; Volkmer, R.; Muller, S.; Pawson, T.; Gingras, A. C.; Arrowsmith, C. H.; Knapp, S., Histone recognition and large-scale structural analysis of the human bromodomain family. *Cell* **2012,** *149* (1), 214-231.

5.2 Kinetic Experiments:

Experimental conditions: 100 µM probe was incubated with 1 µM bromodomain in 20 mM HEPES and 200 mM NaCl at 37 ºC. At given time points (0, 5, 10, 20, 30, 60, 90 and 120 minutes), the solution was sampled (75 µL), purified through a quick solid phase extraction on C4 cartridge and analysed by Rapidfire 360^{TM} MS interfaced with a QTOF. Blank experiments were Values are reported as absolute area of each mass peak.

5.2.1 BTZ 100 µM and BRD4(1) 1 µM

	Deconvoluted Area§						Covalent Modification(%)	
Time (min)	Modified BRD*	SEM	Unmodified BRD	SEM	Total Area	Control	Modified BRD	Unmodified BRD
0	36526.8	1766.3	2604013	76928.3	2640539.4	3188982	1.4	98.6
5	117009.3	4426.6	2181887	27340.2	2298896.3	3386286	5.1	94.9
10	160816.3	6195.6	1817258	71211.5	1978074.3	3274334	8.1	91.9
20	210887.3	4166.6	1658156	15019.7	1869043.3	2809483	11.3	88.7
30	338807.3	26646.0	1425869	52546.8	1764676.7	3199240	19.2	80.8
60	419799.0	15185.4	1160203	13123.3	1580002.3	3426590	26.6	73.4
120	457494.0	21260.8	898539.7	32427.7	1356033.7	3407567	33.7	66.3

§Values are reported as the average of three replicates.

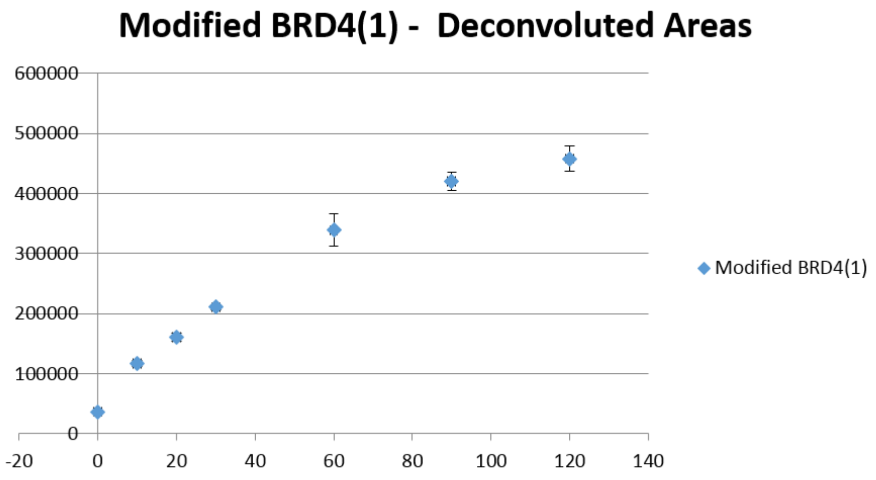

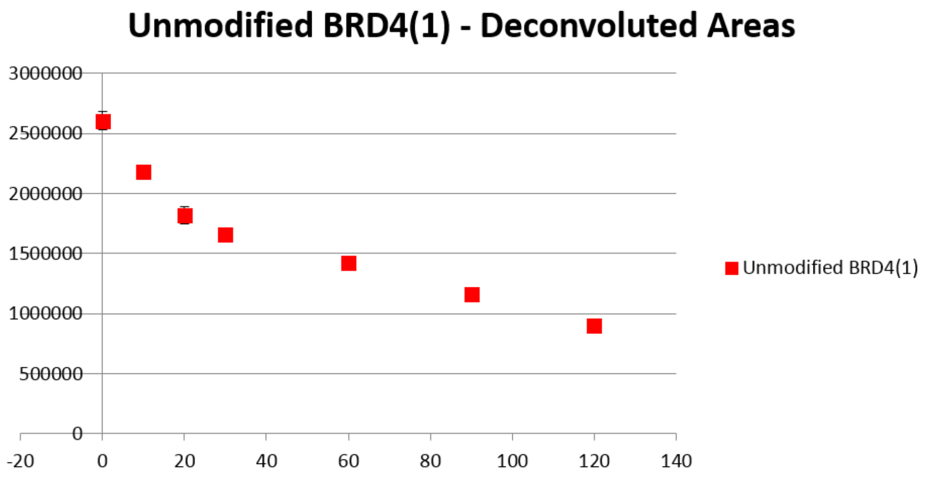

Area values were converted into concentrations (µM) by assuming a direct proportionality between deconvoluted areas and concentrations. The average of deconvoluted areas registered during the control experiment is referred as the concentration of BRD at t=0 ($[A_0]$ = 1 µM) as it doesn't change during the experiment and it takes into account experimental errors. The concentration of unmodified bromodomain at different time points was measured with reference to this value, and so it was the natural logarithm of the ratio between the two (Ln ($[A]/[A_0]$)).

	**Deconvoluted Area**			**Ln ($[A]/[A_0]$)**		
**Time (min)**	**Unmodified BRD**			**Unmodified BRD**		
0	2456753	2639044	2716241	-14.09	-14.02	-13.99
5	2217267	2128091	2200303	-14.195	-14.24	-14.20
10	1922726	1847413	1681635	-14.34	-14.378	-14.47
20	1660671	1630975	1682822	-14.48	-14.50	-14.47
30	1326604	1445614	1505390	-14.71	-14.62	-14.58
60	1155392	1184954	1140264	-14.85	-14.82	-14.86
120	900107	841606	953906	-15.10	-15.16	-15.04

**Kapp (M^{-1} s^{-1})**			**SEM**
0.0001284	0.0001387	0.0001343	2.98385E-06

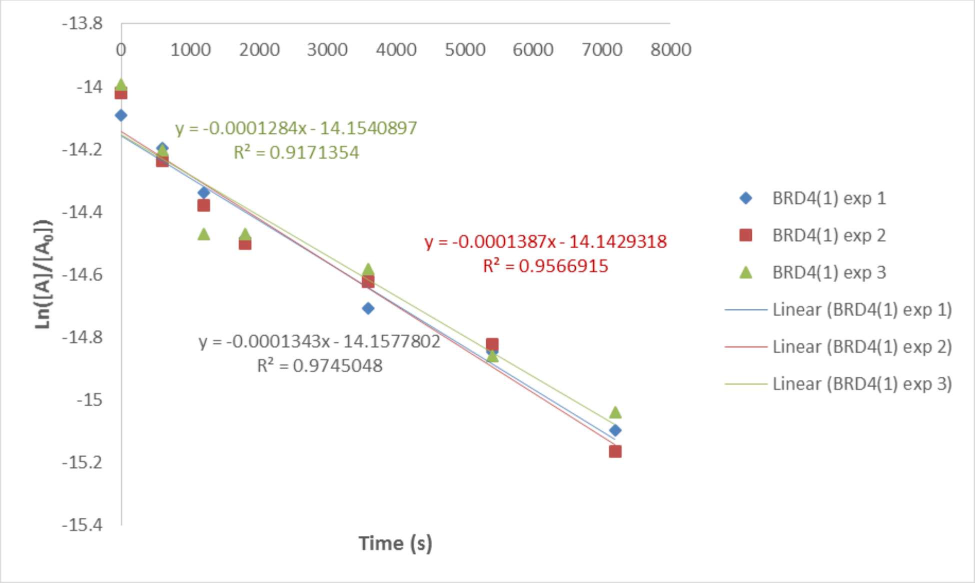

5.2.2 BTZ 100 µM and BRD4(2) 1 µM

	Deconvoluted Area[§]						Covalent Modification(%)	
Time (min)	Modified BRD[*]	SEM	Unmodified BRD	SEM	Total Area	Control	Modified BRD	Unmodified BRD
0	53233.5	3911.545	5085796	333588.2	5139029.8	5406608	1.04	98.95
5	193647.3	20775.18	3719936	447929.2	3913583.7	5328213	4.95	95.05
10	249258.7	32747.87	3191125	449595.5	3440383.7	4786069	7.24	92.75
20	299186.0	35343.81	2839272	361532.2	3138458.0	4779674	9.53	90.47
30	358939.7	42893.1	2264607	298442.5	2623547.0	5076476	13.68	86.32
60	337781.7	42835.41	1679708	222426.4	2017489.7	4923649	16.74	83.26
120	331434.0	50953.44	1362977	212741.9	1694410.7	4860158	19.56	80.44

[§]Values are reported as the average of three replicates.

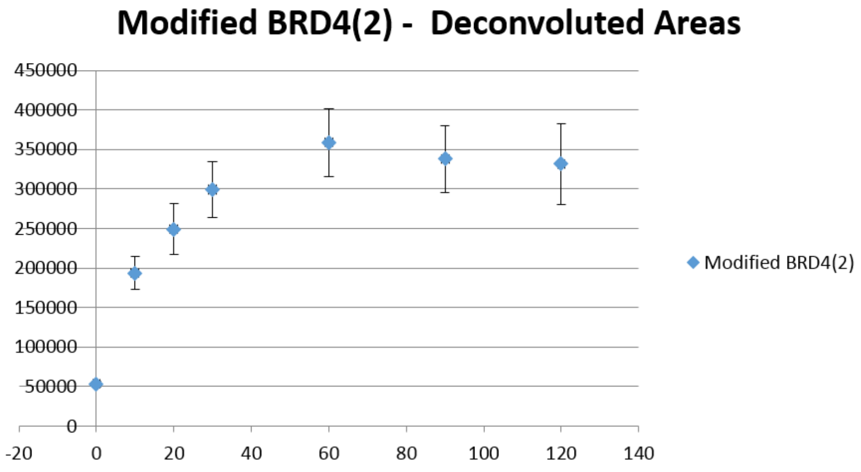

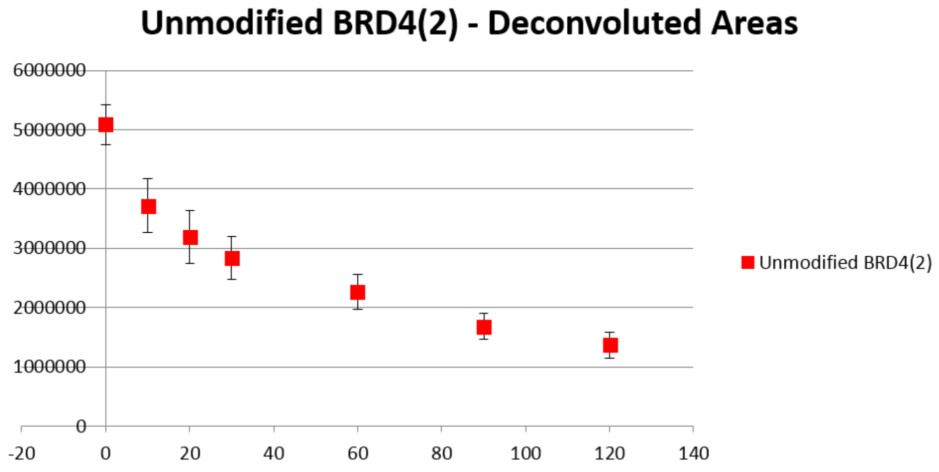

Area values were converted into concentrations (µM) by assuming a direct proportionality between deconvoluted areas and concentrations. The average of deconvoluted areas registered during the control experiment is referred as the concentration of BRD at t=0 ($[A_0]$ = 1 µM) as it doesn't change during the experiment and it takes into account experimental errors. The concentration of unmodified bromodomain at different time points was measured with reference to this value, and so it was the natural logarithm of the ratio between the two (Ln ($[A]/[A_0]$)).

	Deconvoluted Area			Ln ($[A]/[A_0]$)			
**Time (min)**	**Unmodified BRD**			**Unmodified BRD**			
0	5432000	4418781	4114486	-13.7372	-13.9437	-14.015	
5	4610894	3193417	3355498	-13.9011	-14.2684	-14.2189	
10	4090104	2758541	2724730	-14.021	-14.4148	-14.4272	
20	3558287	2545943	2413586	-14.1603	-14.495	-14.5484	
30	2860773	1991896	1941153	-14.3784	-14.7404	-14.7663	
60	2123826	1479783	1435515	-14.6763	-15.0376	-15.068	
120	1788455	1148355	1152120	-14.8482	-15.2912	-15.2879	
				**Kapp (M^{-1} s^{-1})**			**SEM**
				0.000151	0.000167	0.000169	5.696E-06

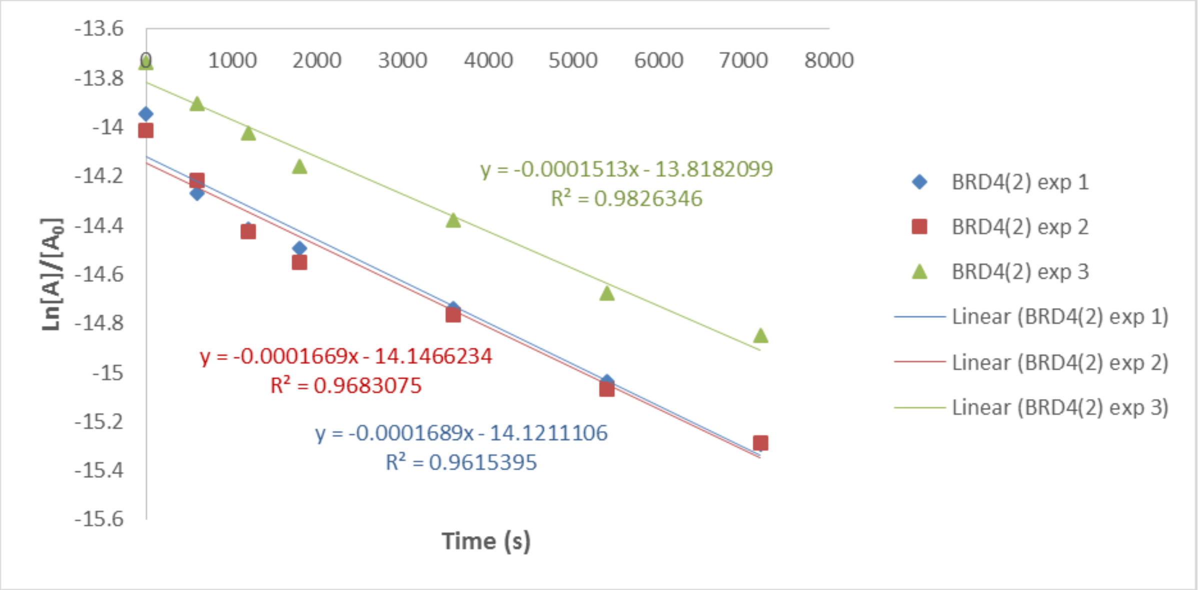

5.2.3 BTZ 100 µM and BRD3(2) 1 µM

	Deconvoluted Area§						Covalent Modification(%)	
Time (min)	Modified BRD*	SEM	Unmodified BRD	SEM	Total Area	Control	Modified BRD	Unmodified BRD
0	81254.25	1999.449	6671653	233733	6752907.25	8153064	1.22	98.78
5	294984	6141.152	5597932	320074.5	5892916	7884086	5.00	94.99
10	448070	19321.28	4858693	305006.1	5306762.667	7118667	8.44	91.56
20	586922.7	24900.61	4694408	172933.8	5281330.667	7423659	11.11	88.89
30	950629	32019.02	4156861	154473.5	5107489.667	7379543	18.61	81.39
60	1251461	28693.38	3598428	106468.9	4849889.333	7395855	25.80	74.20
120	1469713	62007.74	3196837	134724.5	4666550	7445230	31.49	68.51

§Values are reported as the average of three replicates.

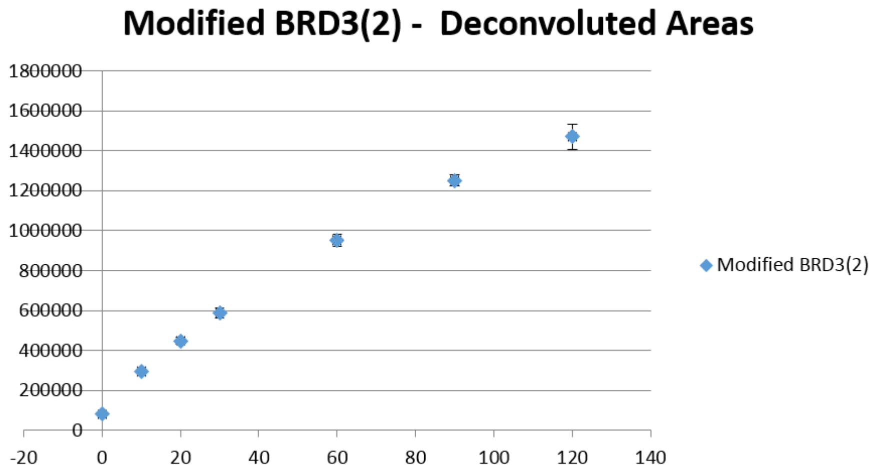

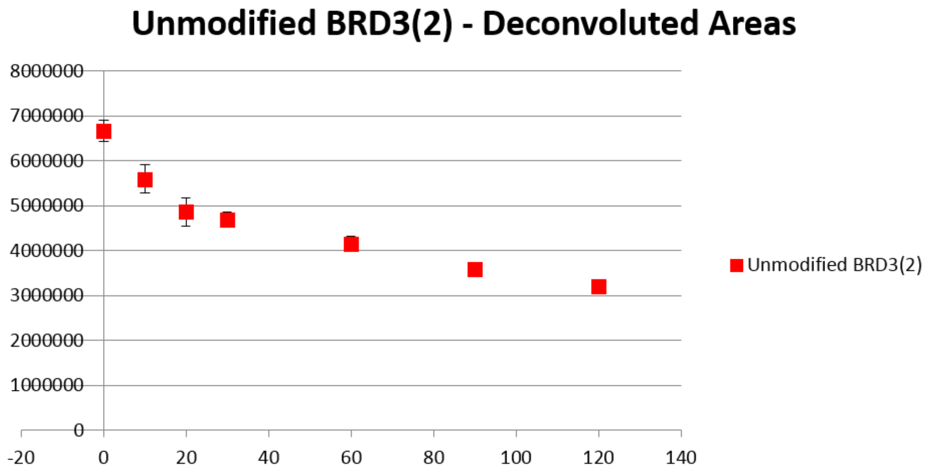

Area values were converted into concentrations (µM) by assuming a direct proportionality between deconvoluted areas and concentrations. The average of deconvoluted areas registered during the control experiment is referred as the concentration of BRD at t=0 ($[A_0]$ = 1 µM) as it doesn't change during the experiment and it takes into account experimental errors. The concentration of unmodified bromodomain at different time points was measured with reference to this value, and so it was the natural logarithm of the ratio between the two (Ln ($[A]/[A_0]$)).

	Deconvoluted Area			Ln ($[A]/[A_0]$)			
**Time (min)**	**Unmodified BRD**			**Unmodified BRD**			
0	7069980	6684374	6260605	-13.88	-13.94	-14.01	
5	6191364	5509115	5093317	-14.01	-14.13	-14.22	
10	5448994	4696750	4430334	-14.14	-14.29	-14.43	
20	5038357	4490928	4553939	-14.22	-14.33	-14.55	
30	4416571	4171916	3882095	-14.35	-14.41	-14.77	
60	3776372	3610742	3408170	-14.51	-14.55	-15.07	
120	3433840	3189349	2967321	-14.60	-14.68	-15.29	
				**Kapp (M^{-1} s^{-1})**			**SEM**
				0.0000894	0.0000876	0.0000945	2.0664E-06

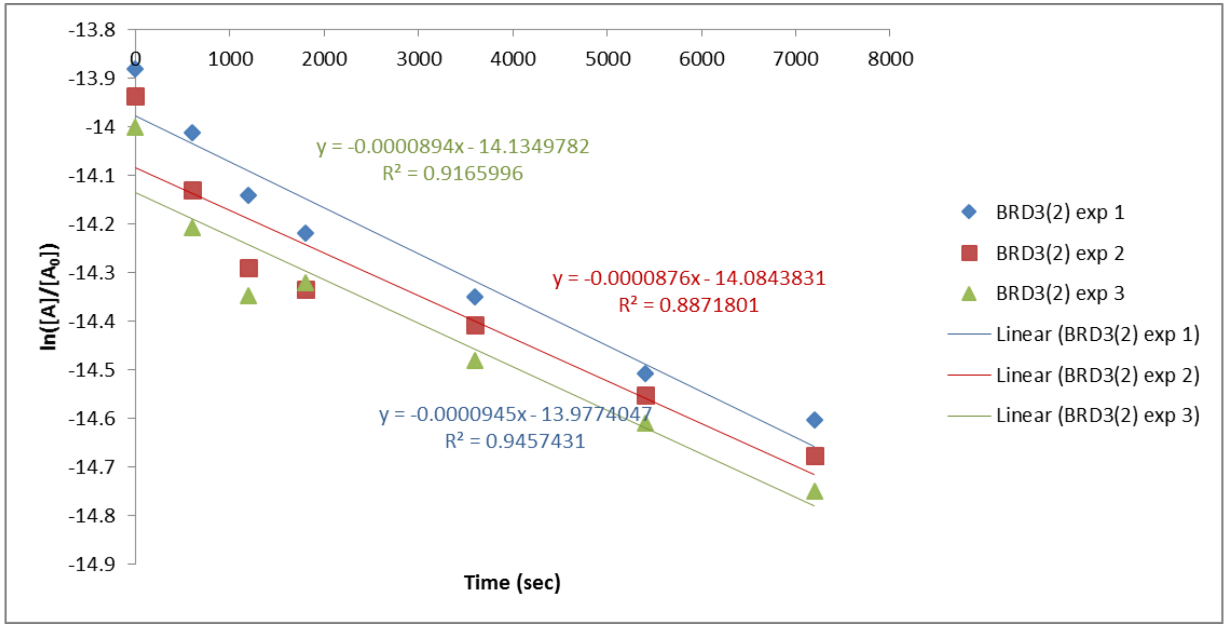

5.2.4 BTZ 100 µM and BRD2(1) 1 µM

	Deconvoluted Area§						Covalent Modification(%)	
Time (min)	Modified BRD*	SEM	Unmodified BRD	SEM	Total Area	Control	Modified BRD	Unmodified BRD
0	81815.33	23270.16	938788	50315.13	1020603.333	-	1.22	98.78
5	132810	1247.993	718016.3	5128.345	850826.33	1240639	5.00	94.99
10	165098.3	14815.35	557304	28132.34	722402.33	1938300	8.44	91.56
20	192926.7	7941.703	479827	6970.478	672753.67	1656247	11.11	88.89
30	230945.7	1777.656	308839	11339.18	539784.67	1661703	18.61	81.39
60	264628	7012.734	220126.3	11230.46	484754.33	1769332	25.80	74.20
120	283767	18529.09	136103.3	9030.167	419870.33	-	31.49	68.51

§Values are reported as the average of three replicates.

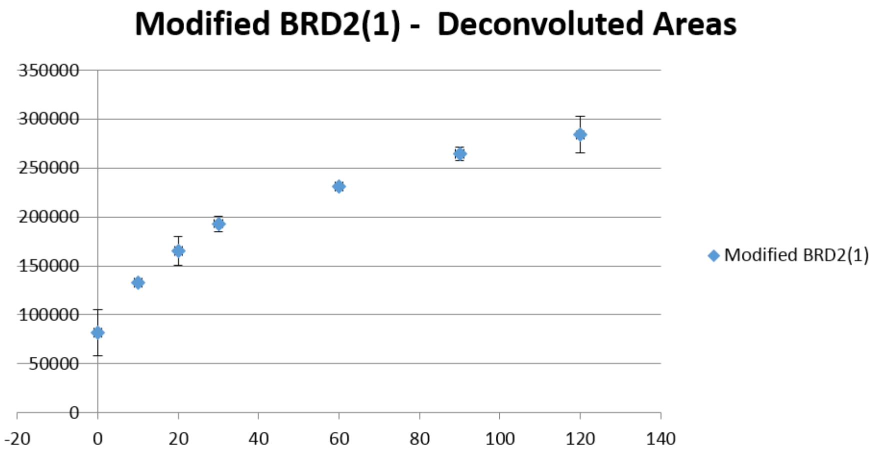

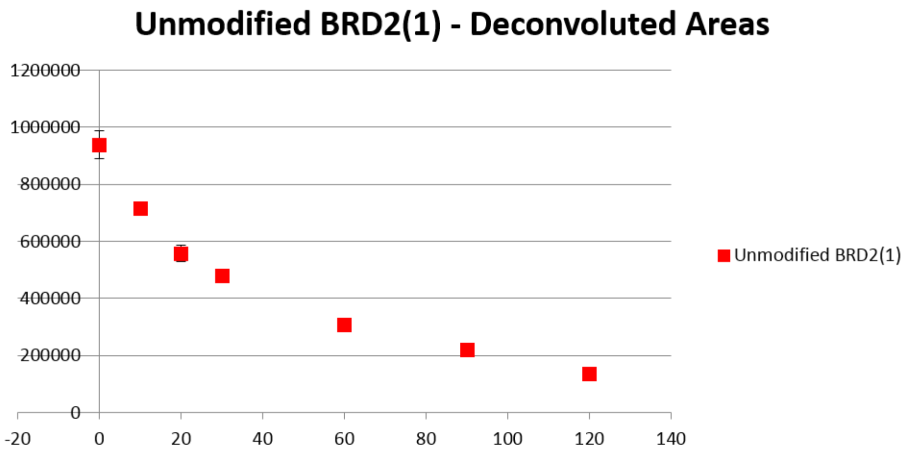

Area values were converted into concentrations (µM) by assuming a direct proportionality between deconvoluted areas and concentrations. The average of deconvoluted areas registered during the control experiment is referred as the concentration of BRD at t=0 ($[A_0]$ = 1 µM) as it doesn't change during the experiment and it takes into account experimental errors. The concentration of unmodified bromodomain at different time points was measured with reference to this value, and so it was the natural logarithm of the ratio between the two (Ln ($[A]/[A_0]$)).

	Deconvoluted Area			Ln ($[A]/[A_0]$)			
Time (min)	Unmodified BRD			Unmodified BRD			
0	-	-	-	-	-	-	
5	728101	711354	714594	-14.64	-14.66	-14.65	
10	547093	514492	610327	-14.92	-14.98	-14.81	
20	467964	479417	492100	-15.08	-15.05	-15.03	
30	316684	323344	286489	-15.47	-15.45	-15.57	
60	242515	207373	210491	-15.73	-15.89	-15.88	
120	152873	133525	121912	-16.20	-16.33	-16.42	
					Kapp (M^{-1} s^{-1})		SEM
				0.0002400	0.0002193	0.0002628	1.25622E-05

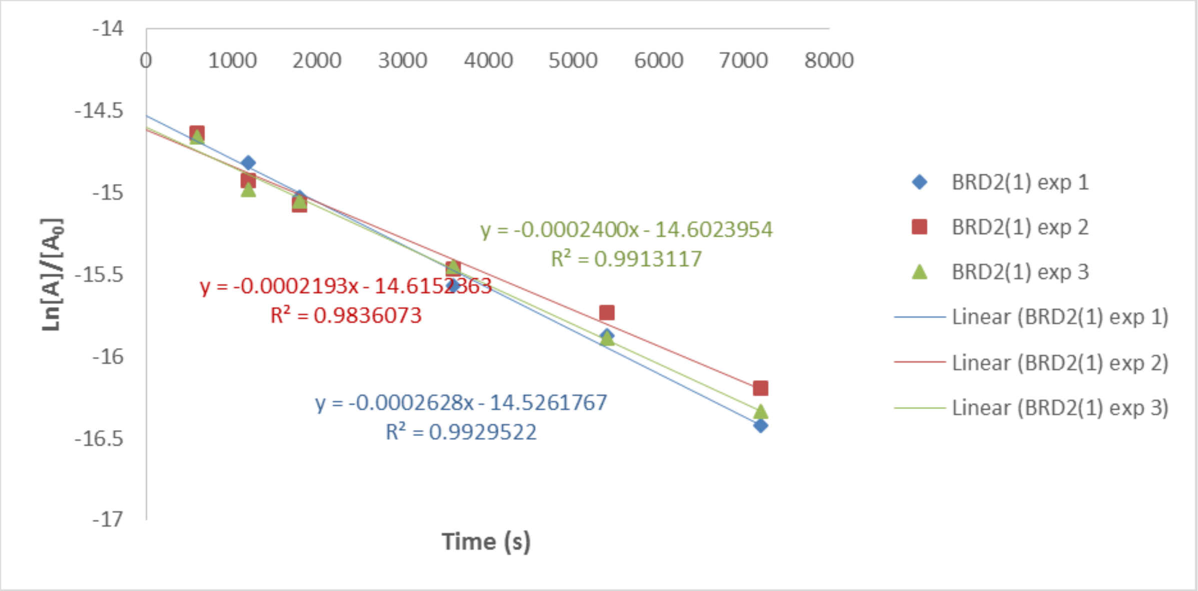

5.2.5 BTZ 100 µM and BRD9 1 µM

	Deconvoluted Area[§]						Covalent Modification(%)	
Time (min)	Modified BRD[*]	SEM	Unmodified BRD	SEM	Total Area	Control	Modified BRD	Unmodified BRD
0	143217.25	701.114	2768218	205044	2911435.58	4212540	5.17	94.83
5	451175.00	12569.0	1676612	160157.4	2127787.33	3937281	21.20	78.80
10	599059.33	28274.96	1152637	128901.6	1751696.33	3754996	34.20	65.80
20	720230.67	46715.20	922279.7	98933.23	1642510.33	3796729	43.85	56.15
30	1021967.0	63061.07	491559.7	69587.42	1513526.67	4207068	67.52	32.48
60	911932.33	60404.46	239683.3	35682.94	1151615.67	3953821	79.19	20.81
120	813650.67	57026.19	131675.7	15728.55	945326.33	3814969	86.07	13.93

[§]Values are reported as the average of three replicates.

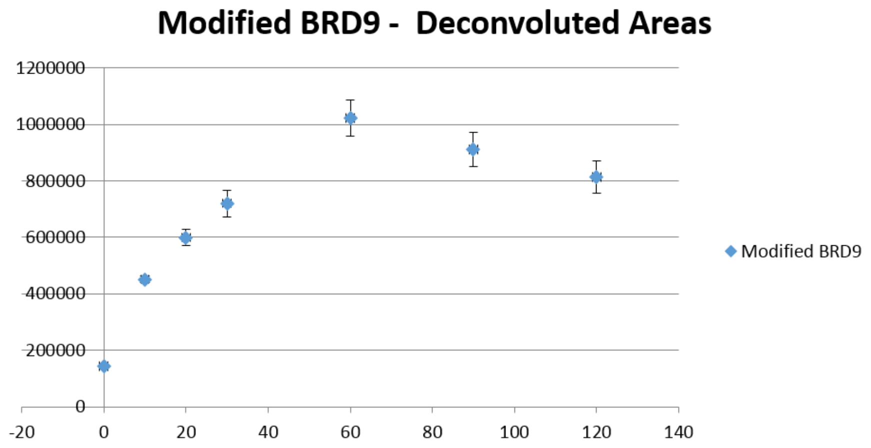

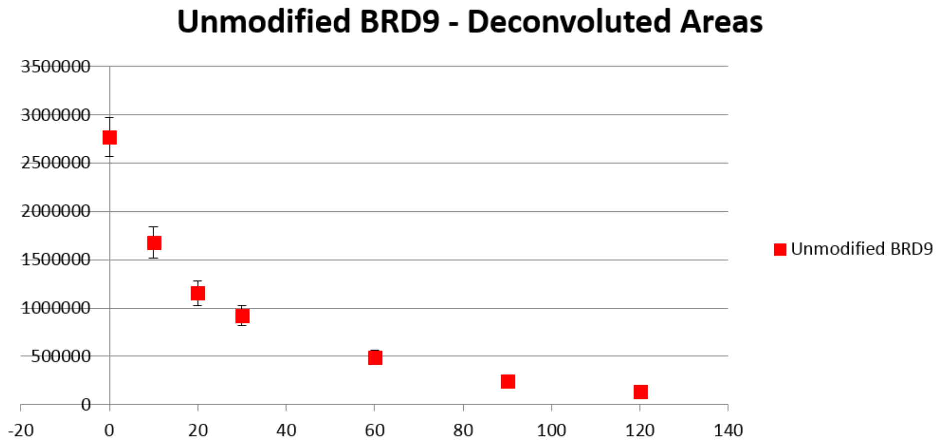

Area values were converted into concentrations (μM) by assuming a direct proportionality between deconvoluted areas and concentrations. The average of deconvoluted areas registered during the control experiment is referred as the concentration of BRD at t=0 ($[A_0]$ = 1 μM) as it doesn't change during the experiment and it takes into account experimental errors. The concentration of unmodified bromodomain at different time points was measured with reference to this value, and so it was the natural logarithm of the ratio between the two (Ln ($[A]/[A_0]$)).

	**Deconvoluted Area**			**Ln ($[A]/[A_0]$)**		
**Time (min)**	**Unmodified BRD**			**Unmodified BRD**		
0	3170817	2634483	2499355	-14.04	-14.22	-14.27
5	1991469	1570176	1468192	-14.50	-14.74	-14.80
10	1390391	1120083	947437	-14.86	-15.08	-15.24
20	1118867	843439	804533	-15.08	-15.36	-15.41
30	630328	431381	412970	-15.65	-16.03	-16.07
60	295894	249658	173498	-16.41	-16.58	-16.94
120	151423	143008	100596	-17.078	-17.13	-17.49

**Kapp (M^{-1} s^{-1})**			**SEM**
0.0003832	0.0003998	0.0004271	1.27977E-05

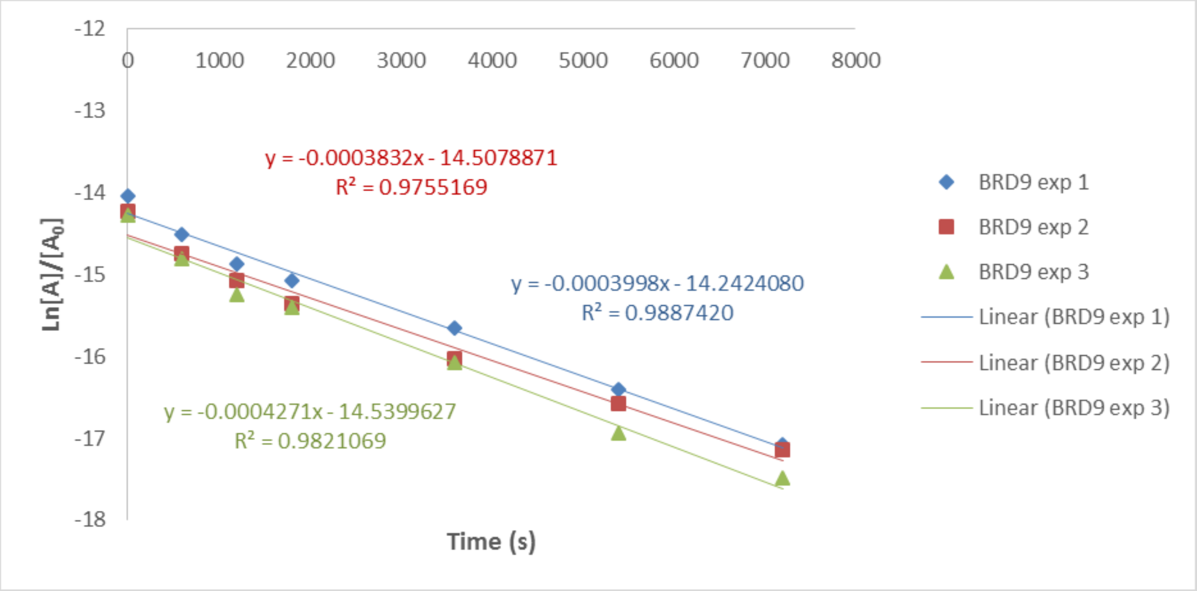

5.2.6 BTZ 100 µM and CECR2 1 µM

	Deconvoluted Area§						Covalent Modification(%)	
Time (min)	Modified BRD*	SEM	Unmodified BRD	SEM	Total Area	Control	Modified BRD	Unmodified BRD
0	291816.3	28245.24	2880204	128957	3172020.25	4754567	10.13	89.87
5	1117459	25024.41	709792.3	56518.17	1827251.667	3098162	61.16	38.84
10	1340677	35573.99	323764	23707.48	1664440.667	2811445	80.55	19.45
20	1353722	63716.98	149059.3	14036.28	1502781.667	2679638	90.08	9.92
30	1396766	64443.29	25539	4567.453	1422304.667	2870240	98.20	1.80
60	1366313	14702.47	0	0	1366312.667	2988766	100	0
120	1295672	36321.66	0	0	1295672.333	2886016	100	0

§Values are reported as the average of three replicates.

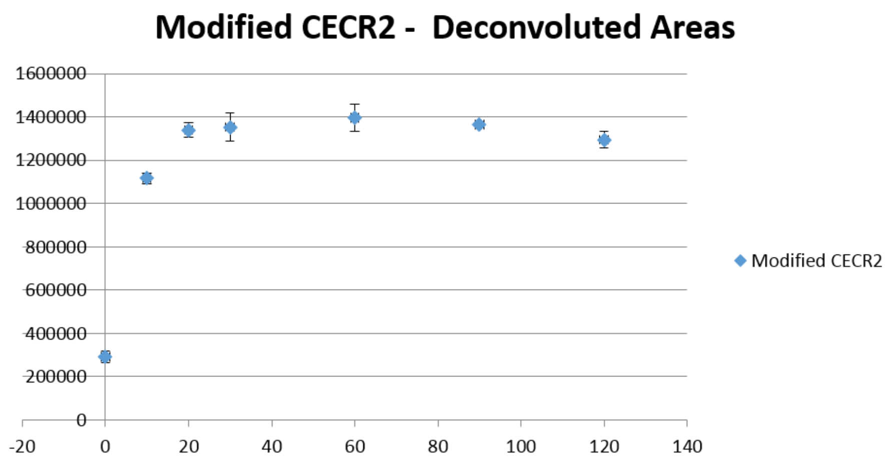

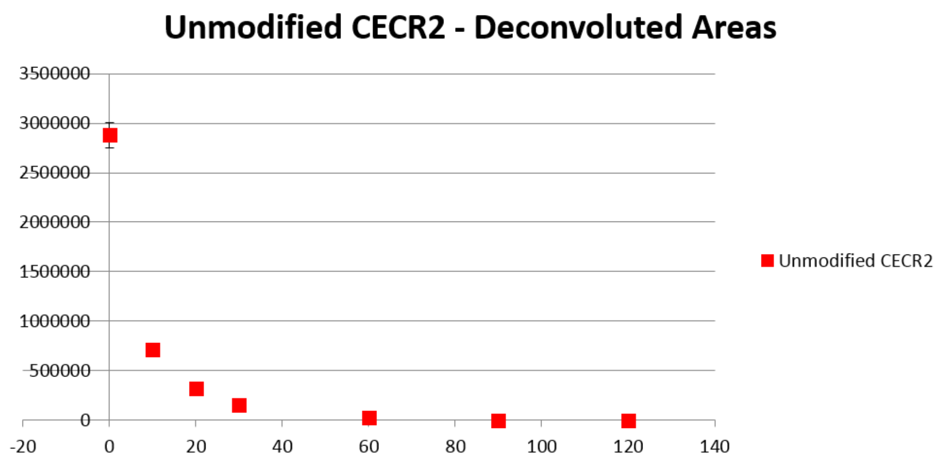

Area values were converted into concentrations (µM) by assuming a direct proportionality between deconvoluted areas and concentrations. The average of deconvoluted areas registered during the control experiment is referred as the concentration of BRD at t=0 ($[A_0]$ = 1 µM) as it doesn't change during the experiment and it takes into account experimental errors. The concentration of unmodified bromodomain at different time points was measured with reference to this value, and so it was the natural logarithm of the ratio between the two (Ln ($[A]/[A_0]$)).

	**Deconvoluted Area**			**Ln ($[A]/[A_0]$)**		
**Time (min)**	**Unmodified BRD**			**Unmodified BRD**		
0	2654396	3101033	2885183	-13.99	-13.83	-13.91
5	817756	684807	626814	-15.17	-15.34	-15.43
10	369702	310964	290626	-15.96	-16.13	-16.20
20	177112	135948	134118	-16.70	-16.96	-16.97
30	34575	22182	19860	-18.33	-18.77	-18.88
60	0	0	0	-	-	-
120	0	0	0	-	-	-

**Kapp (M^{-1} s^{-1})**			**SEM**
0.0011647	0.0013071	0.001316	4.90174E-05

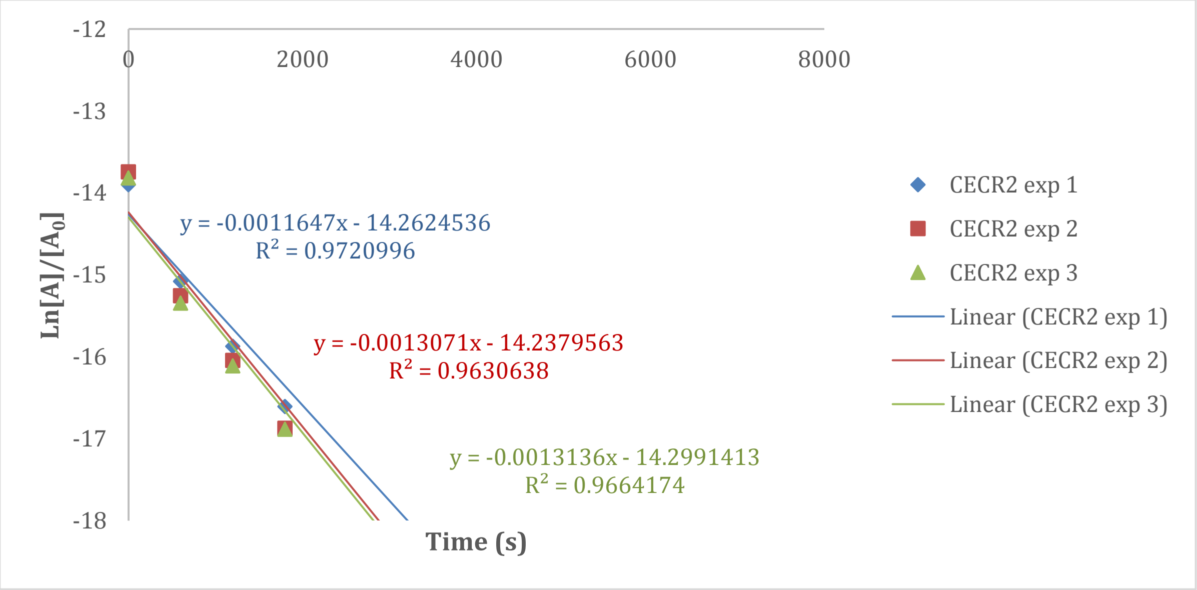

6. LC-MS/MS Analysis for Peptide Identification

For the reaction of recombinant BRD proteins with probe, the following conditions were used: 30 μM probe was incubated for 1.5 hours at 37 ºC in the presence of 3 μM recombinant BRD (total amount of protein around 4 μg), in a buffer containing 20 mM HEPES and 200 mM NaCl, pH 7.4. For the reactions using BRD4(1) and BTZ **2**, 100 μM probe was incubated for 1.5 hours at 37 ºC in the presence of 5 μM recombinant BRD4. Excess of probe material was removed by filtering the sample through a Micro Bio-Spin® column (Bio-Rad). For in-solution digestions, trypsin from bovine pancreas (1 μg/μL in 0.1 HCl, Promega) was reconstituted in 800 μL of 50 mM ammonium bicarbonate, and 4 μL of this solution were added to 100 μL of the 3 μM solution of BRD (+ probe). The solution was incubated overnight at 37 ºC. After the digestion was complete, the obtained peptides were purified by Sep-PAK C-18 solid-phase extraction according to the manufacturer's instructions (Waters) and concentrated under vacuum and kept at -20 ºC until LC-MS/MS analysis as described previously (Fischer & Kessler, *Proteomics*, 2015).[16] In brief, dried samples were reconstituted in 0.1% TFA in 1% Acetonitrile and analysed on Q Exactive mass spectrometer (Thermo) coupled with a Dionex Ultimate 3000 UPLC (Thermo). Samples were desalted online (PepMAP C18, 300µm x 5mm, 5 µm particle, Thermo) for 1 minute at a flow rate of 20 µl/min and separated on a nEASY column (PepMAP C18, 75 µm x 500 mm, 2 µm particle, Thermo) over 60 Minutes using a gradient of 2%-35% Acetonitrile in 5% DMSO/0.1% Formic acid at 250nl/min. Survey scans were acquired at a resolution of 70,000 @ 200m/z and the 15 most abundant precursors were selected for HCD fragmentation. Alternatively, samples were analysed using an Orbitrap Velos mass spectrometer (Thermo) coupled to a nAquity UPLC system (Waters). Briefly, samples were desalted online (Symmetry C18 column (180 µm x 20mm, 5 µm particle, Waters) for 5 minutes at a flow rate of 5µl/min, followed by separation on a BEH C18 column (75 µm × 250 mm, 1.7 µm particle, Waters) over 60 minutes using a gradient of 2%-40% Acetonitrile in 0.1% Formic acid at 250nl/min. Survey scans were acquired in the Orbitrap at a resolution of 60.000 @ 400m/z and the 20 most abundant precursors were selected for CID fragmentation. MS data were analysed using PEAKS software (version 7.5) as described.[17]

Figure Legend: ***Mapping BRD-probe crosslinking sites by MS/MS*. A**) (top left panel) MS/MS spectrum of the BRD4(1) derived tryptic peptide 77-99 containing K91 that was found to be modified by BTZ (**2**) (+462.12 Da). Matching fragment ions are indicated in red (y-ions) and blue (b-ions). (top right panel) sequence coverage indicating the detection of a tryptic peptide containing modified K91 (in blue lines) as identified by PEAKS (red box). (lower left panel) Calculated MS/MS fragment ions (black) that match the experimentally observed y-ions (red) and b-ions (blue). (Lower right panel) BRD4(1) structure indicating a direct interaction between K91 and BTZ (**2**). **B**) (top left panel) MS/MS spectrum of the BRD3(2) derived tryptic peptide 327-346 containing K336 that was found to be modified by BTZ (**2**) (+462.12 Da). Matching fragment ions are indicated in red (y-ions) and blue (b-ions). (top right panel) sequence coverage indicating the detection of a tryptic peptide containing modified K336 (in blue lines) as identified by PEAKS (red box). (lower left panel) Calculated MS/MS fragment ions (black) that match the experimentally observed y-ions (red) and b-ions (blue). (Lower right panel) BRD3(2) structure indicating a direct interaction between K336 and BTZ (**2**).

(16) Fischer, R. & Kessler, B. M. Gel-aided sample preparation (GASP)—A simplified method for gel-assisted proteomic sample generation from protein extracts and intact cells. *Proteomics* **2015**, *15* (7), 1224-1229.
(17) Davis, S.; Charles, P. D.; He, L.; Mowlds, P.; Kessler, B. M & Fischer, R. Expanding Proteome Coverage with CHarge Ordered Parallel Ion aNalysis (CHOPIN) Combined with Broad Specificity Proteolysis. *J. Proteome Res.* **2017**, *16*, 1288-1299.

6.1 BTZ & BRD4(1) – Lysine 91

A ▪ BRD4(1) - recombinant

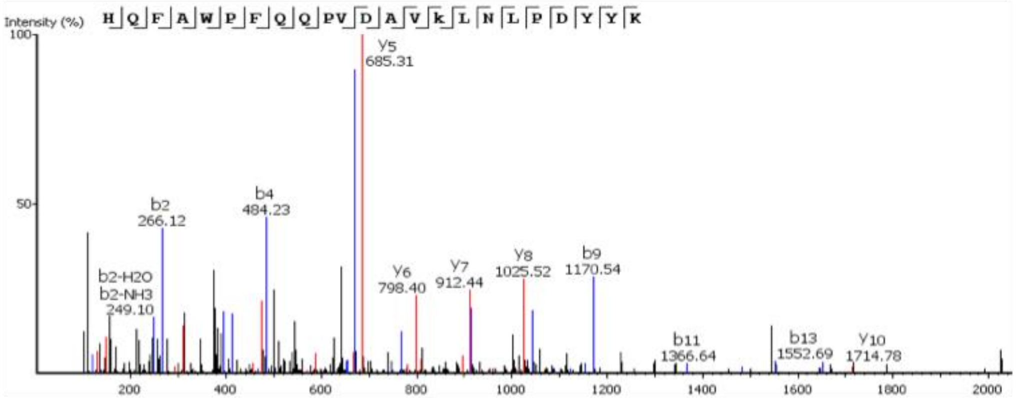

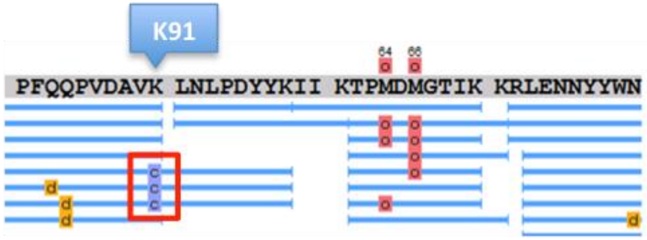

▪ **QE MS/MS BRD4(1) 5 uM – BTZ 100 uM**

#	b	b-H2O	b-NH3	b (2+)	Seq	y	y-H2O	y-NH3	y (2+)	#
1	138.07	120.08	121.04	69.53	H					23
2	266.12	248.11	249.10	133.56	Q	3129.48	3111.47	3112.45	1565.24	22
3	413.19	395.18	396.18	207.10	F	3001.42	2983.41	2984.39	1501.21	21
4	484.23	466.22	467.20	242.62	A	2854.35	2836.34	2837.33	1427.68	20
5	670.30	652.30	653.28	335.66	W	2783.32	2765.31	2766.29	1392.16	19
6	767.35	749.35	750.31	384.18	P	2597.24	2579.23	2580.21	1299.12	18
7	914.43	896.41	897.41	457.72	F	2500.18	2482.17	2483.16	1250.59	17
8	1042.48	1024.48	1025.46	521.74	Q	2353.12	2335.10	2336.09	1177.06	16
9	1170.54	1152.54	1153.50	585.77	Q	2225.06	2207.05	2208.03	1113.03	15
10	1267.60	1249.59	1250.57	634.30	P	2097.00	2078.99	2079.97	1049.00	14
11	1366.64	1348.66	1349.64	683.83	V	1999.95	1981.93	1982.92	1000.47	13
12	1481.68	1463.69	1464.67	741.35	D	1900.88	1882.87	1883.85	950.94	12
13	1552.69	1534.72	1535.71	776.87	A	1785.85	1767.84	1768.82	893.43	11
14	1651.78	1633.79	1634.78	826.40	V	1714.78	1696.80	1697.79	857.91	10
15	2242.02	2224.01	2224.99	1121.50	K(+462.12)	1615.74	1597.73	1598.72	808.36	9
16	2355.10	2337.09	2338.07	1178.05	L	1025.52	1007.52	1008.50	513.27	8
17	2469.14	2451.13	2452.12	1235.07	N	912.44	894.44	895.41	456.72	7
18	2582.23	2564.22	2565.20	1291.61	L	798.40	780.38	781.40	399.70	6
19	2679.28	2661.27	2662.25	1340.14	P	685.31	667.30	668.29	343.16	5
20	2794.31	2776.30	2777.28	1397.65	D	588.26	570.25	571.24	294.63	4
21	2957.37	2939.36	2940.34	1479.19	Y	473.24	455.23	456.23	237.12	3
22	3120.43	3102.42	3103.41	1560.72	Y	310.17	292.16	293.15	155.59	2
23					K	147.11	129.10	130.09	74.06	1

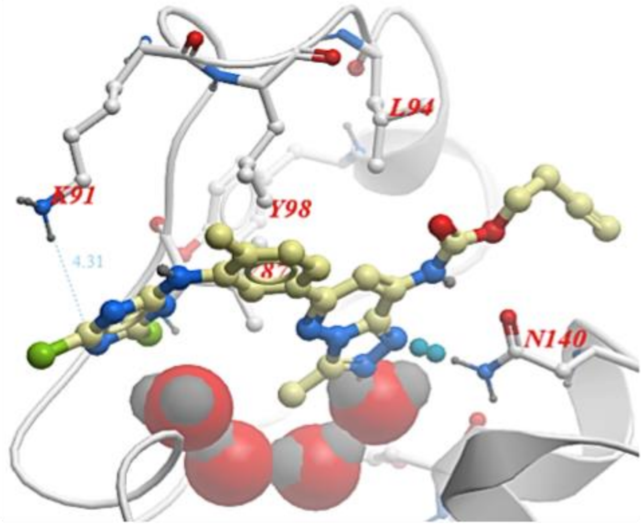

6.2 BTZ & BRD3(2) – Lysine 336

B

- **BRD3(2) - recombinant**

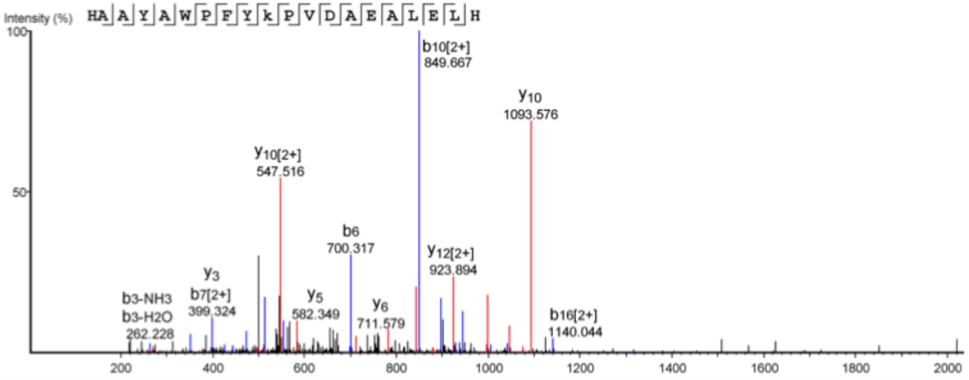

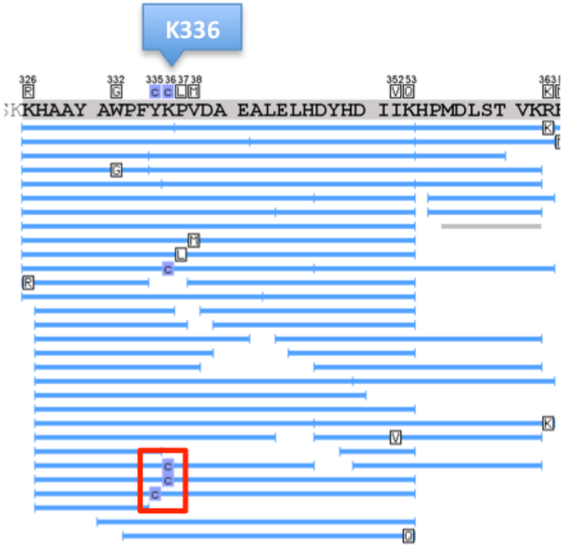

- **Orbitrap MS/MS BRD3(2) 3 uM – BTZ 30 uM**

#	Immonium	b	b-H2O	b-NH3	b (2+)	Seq	y	y-H2O	y-NH3	y (2+)	#
1	110.072	138.067	120.056	121.040	69.533	H					20
2	44.050	209.247	191.093	192.077	105.052	A	2653.205	2635.195	2636.178	1327.103	19
3	44.050	280.141	262.228	263.324	140.570	A	2582.168	2564.157	2565.141	1291.584	18
4	136.076	443.323	425.308	426.177	222.102	Y	2511.131	2493.120	2494.104	1256.065	17
5	44.050	514.256	496.377	497.214	257.621	A	2348.068	2330.057	2331.041	1174.534	16
6	159.092	700.317	682.310	683.294	350.596	W	2277.031	2259.020	2260.003	1139.015	15
7	70.066	797.373	779.562	780.346	399.324	P	2090.951	2072.941	2073.924	1045.525	14
8	120.081	944.508	926.431	927.412	472.693	F	1993.898	1975.888	1976.871	997.429	13
9	136.076	1107.814	1089.495	1090.914	554.604	Y	1846.830	1828.820	1829.803	923.894	12
10	563.227	1697.719	1679.709	1680.693	849.667	K(+462.12)	1683.767	1665.756	1666.740	842.692	11
11	70.066	1794.772	1776.762	1777.745	897.433	P	1093.576	1075.709	1076.792	547.516	10
12	72.081	1893.841	1875.830	1876.814	947.420	V	996.671	978.543	979.473	498.750	9
13	88.040	2008.868	1990.857	1991.841	1004.748	D	897.433	879.281	880.064	449.216	8
14	44.050	2079.905	2061.894	2062.878	1040.669	A	782.396	764.877	765.377	391.702	7
15	102.055	2208.947	2190.937	2191.920	1104.974	E	711.579	693.433	694.340	356.422	6
16	44.050	2279.984	2261.974	2262.957	1140.044	A	582.349	564.362	565.585	291.772	5
17	86.097	2393.069	2375.058	2376.042	1197.034	L	511.353	493.157	494.335	256.234	4
18	102.055	2522.111	2504.101	2505.084	1261.556	E	398.145	380.237	381.176	199.602	3
19	86.097	2635.195	2617.185	2618.168	1317.614	L	269.121	251.202	252.134	135.080	2
20	110.072					H	156.077	138.066	139.050	78.538	1

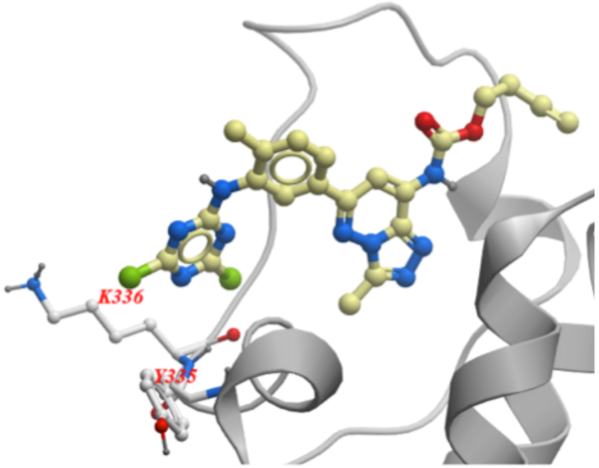

Supplement: Supplementary file 1 — Supplementary [file ANIE-58-1007-s001.pdf]
